# Assembling Di- and Polynuclear Cu(I) Complexes with Rigid Thioxanthone-Based Ligands: Structures, Reactivity, and Photoluminescence

**DOI:** 10.1021/acs.inorgchem.4c03819

**Published:** 2024-12-16

**Authors:** Mohammad Zafar, Vasudevan Subramaniyan, Kamal Uddin Ansari, Hadar Yakir, David Danovich, Yuri Tulchinsky

**Affiliations:** ‡ Institute of Chemistry, Hebrew University of Jerusalem, Jerusalem 9190401, Israel

## Abstract

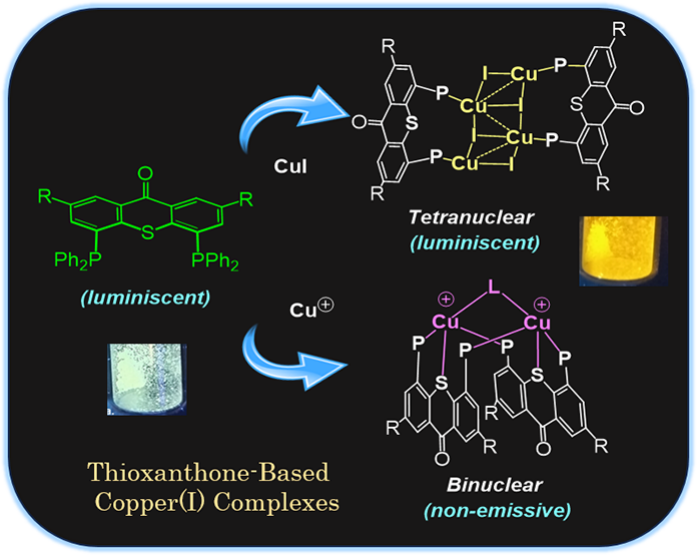

Thioxanthone (TX) molecules and their derivatives are well-known photoactive compounds. Yet, there exist only a handful of luminescent systems combining TX with transition metals. Recently, we reported a TX-based PSP pincer ligand (**L1**) that appears as a promising platform for filling this niche. Herein, we demonstrate that with Cu(I) this ligand exclusively assembles into dimeric structures with either di- or polynuclear Cu(I) cores. With cationic Cu(I) precursors, complexes featuring solvent-bridged bis-cationic cores were obtained. These coordinatively unsaturated bimetallic systems showed surprisingly facile activation of the chloroform C–Cl bonds, suggesting a possible metal–metal cooperation. The reaction of **L1** with binary Cu(I) halides afforded dimeric complexes with polynuclear [CuX]_*n*_ (*n* = 3 or 4) cores. With X = Br or I, emissive complexes containing stairstep [CuX]_4_ clusters were obtained. Emission lifetimes in the microsecond range measured for these complexes were indicative of a triplet emission (phosphorescence), which according to our time-dependent density functional theory study originates from a halide-metal-to-ligand charge transfer between the [CuX]_4_ cluster and the TX backbone of **L1**. Finally, the distinctive polynucleating behavior of **L1** toward Cu(I) was also showcased by a comparison to another PSP ligand with a diaryl thioether backbone (**L2**), which formed only mononuclear pincer-type complexes, lacking any unusual reactivity or photoluminescence.

## Introduction

Well-defined bimetallic complexes, featuring two metal centers in close proximity to each other, have recently gained significant attention for their potential to promote unique molecular transformations through metal–metal cooperation (MMC).^1−3^ Such dinuclear complexes might offer various advantages over their mononuclear counterparts with respect to catalysis,^1,4−6^ including cooperative substrate activation,^7−9^ the ability to store multiple redox equivalents,^10^ and the possibility of mediating multielectron processes.^11^

**Figure 1:**
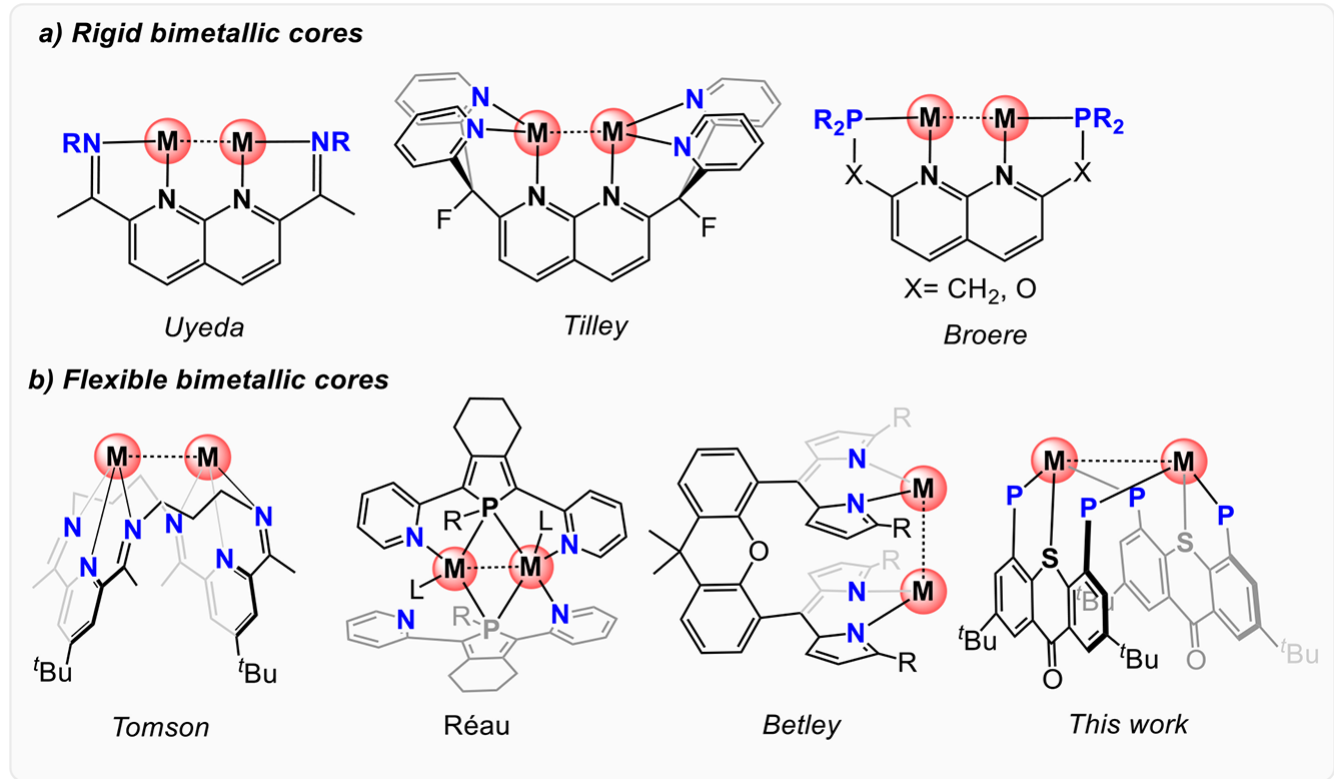
Rigid (a) and flexible (b) structural motifs capable of binding two metals in close proximity to each other.

Ligand platforms capable of stabilizing such bimetallic cores are relatively limited, especially those leading to systems where the two metals are not only found in close proximity but also feature open coordination sites necessary for catalytic activity.^11,12^ Rigid dinucleating ligands based on 1,8-naphthyridine, represent one of the most popular scaffolds for constructing such homo-^13−15^ and heterobimetallic^16−18^ complexes capable of unique reactivity due to cooperative substrate activation^19−21^ (Figure 1a). For instance, Uyeda’s group showed that redox-active 1,8-naphthyridinediimine (NDI) can stabilize M–M-bonded dinickel complexes in five successive oxidation states.^10^ Those systems proved capable of catalyzing a plethora of challenging transformations, such as alkene cyclopropanation,^12^ alkyne trimerizations,^19^ and the unprecedented [2 + 1] and [4 + 1] vinylidene cycloadditions,^7^ none of which can be accomplished with mononuclear Ni complexes. Other examples of MMC involved the use of flexible dinucleating scaffolds (Figure 1b), such as a macrocyclic ligand first introduced by Drew and Nelson^20^ and later revisited by Tomson,^21^ comprising two pyridiyldiimine pincer units tethered together by propylene linkers. In addition to being redox-active, this scaffold is also highly flexible, allowing a facile interchange between open stairstep-shaped and folded conformations, which, in turn, strongly affects the intermetallic distance in its complexes with diiron, dicobalt, dinickel, dicopper, and disilver cores, as well as tricopper clusters.^21−25^ This ligand plasticity rendering it highly adaptable to various oxidation and spin states of the two metals, and the extent of their bonding, was proven crucial for achieving remarkable reactivity of these complexes in a cooperative activation of small molecules, such as MeCN, N_3_^–^, and N_2_. Furthermore, few examples of μ_2_- and μ_3_-bridging pnictogen-based polynuclear complexes (Pn = P, Sb, Bi) have been previously reported.^26,27^ For instances, the Réau group reported dinucleating phosphole-based tridentate ligands capable of stabilizing homo- and heterobimetallic complexes.^28−30^

Among the catalytically active homobimetallic systems of dinucleating ligands, a special place is reserved for the dinuclear Cu(I) cores. For instance, bis-cationic dicopper complexes^13,31^ supported by the pyridine-functionalized 1,8-napthyridine scaffolds reported by Tilley et al. were capable of promoting aryl transfer^32^ and alkyne–azide cycloaddition reactions.^33^ In parallel, Broere et al. have focused on related dicopper(I) complexes of diphosphine-functionalized 1,8-naphthyridine-based ligands (PNNP) for cooperative H_2_ activation^9^ and formate reduction.^34^ In addition to catalysis, dicopper(I) cores were shown to be capable of trapping and stabilizing various reactive species,^35^ with the remarkably stable μ-nitrenoid dicopper complex of a xanthene-derived dinucleating ligand prepared by Betley et al.^36^ being one of the recent examples.

**Figure 2:**
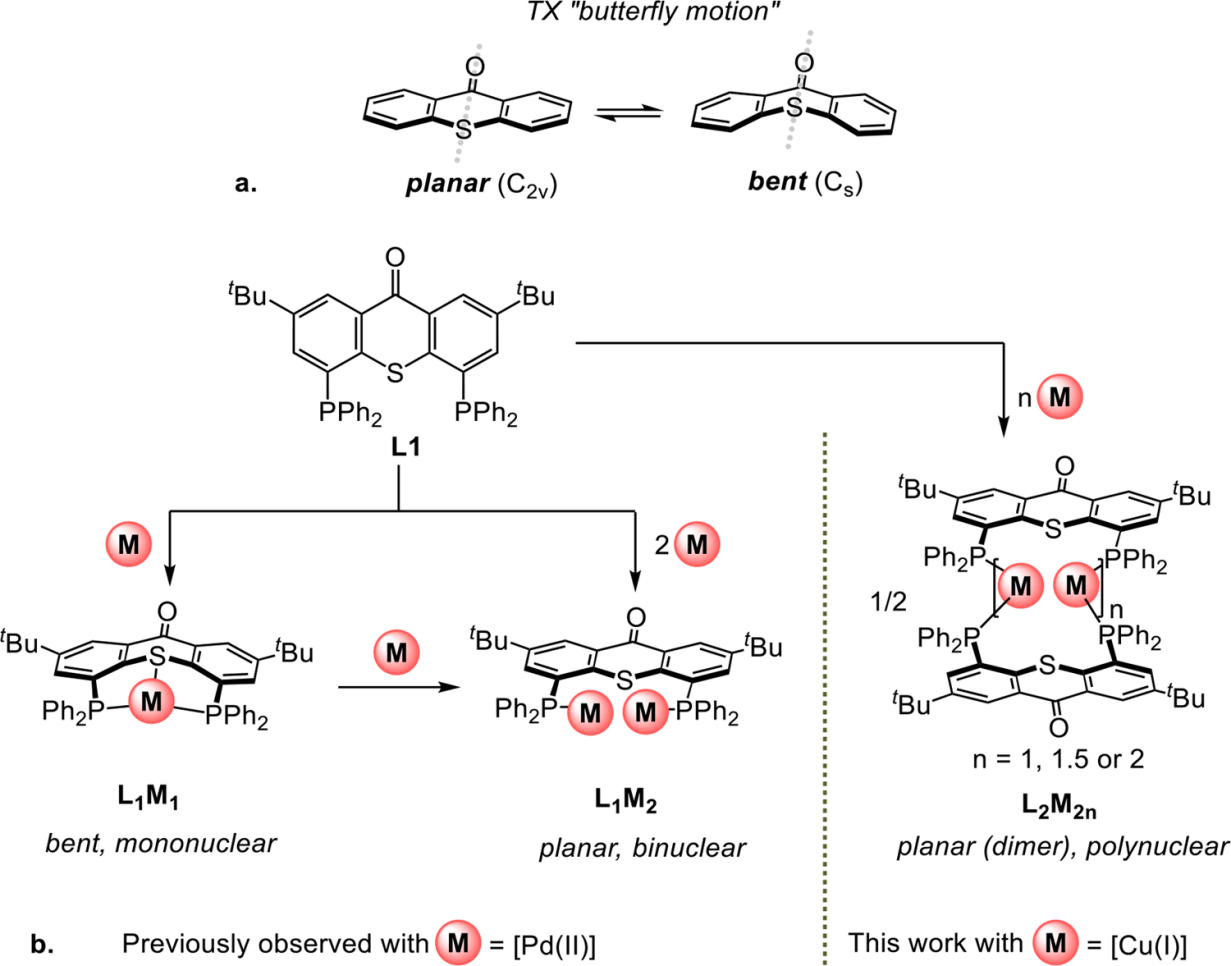
Butterfly-like motion of TX (a) and coordination modes of a TX-based PSP ligand with various metals (b).

Recently, we reported a novel pincer-type PSP ligand featuring a thioxanthone (TX) backbone (**L1**) and provided an experimental and theoretical study of its coordinative behavior.^37^ The parent TX molecule is known to perform a “butterfly motion”,^38^ i.e., interchanging between planar (*C*_2*v*_) and bent (*C*_*s*_) conformations by folding along its S–C=O axis (Figure 2a). Accordingly, ligand **L1** was found to be capable of exhibiting both planar and bent coordination modes, depending on the nuclearity of the resulting complexes (Figure 2b).^37^ This occurs because the distance between its two phosphine donors (ca. 5.4 Å) in the planar conformation is too big for chelating a single metal center but is just right for coordinating a bimetallic core. Our calculations indicated that, due to the high internal ligand strain (ILS) within **L1**, its planar conformation is energetically preferable over the bent one. Indeed, in contrast to the thioxanthene-based structural analogue (with a lower ILS), which maintained a mononuclear κ^3^-P,S,P pincer coordination mode even in the presence of excess (PdCl_2_) precursor, ligand **L1** formed a binuclear κ^2^-P,P complex featuring a (μ-Cl)_2_-bridged dipalladium core.^37^ We therefore wondered whether this pronounced preference of ligand **L1** toward the formation of binuclear complexes can also be leveraged for the stabilization of related dicopper(I) systems, potentially useful for cooperative bimetallic catalysis.

Besides the purely structural aspects, the TX-based backbone of ligand **L1** opens yet another fascinating exploration avenue because TX and its derivatives are well-known for their unique photophysical properties. Those molecules have found extensive applications as triplet sensitizers^39,40^ or photoinitiators in organic synthesis^41−43^ and light-harvesting elements in luminescent materials^44,45^ due to the relatively small energy gap (∼0.3 eV) between their lowest singlet and triplet excited states (S_1_ and T_1_, respectively), which enhances efficient intersystem crossing.^46^ Unfortunately, phosphorescence of numerous organic TX derivatives is often suppressed by the competing rapid nonradiative decay processes, precluding full realization of these compounds as efficient triplet emitters. Surprisingly, unlike purely organic TX derivatives, there have been relatively few photophysical studies on mixed TX/transition metal (TM) systems. In several cases, TX moieties have been covalently appended to emissive TM and lanthanide complexes, serving as antenna chromophores.^47^ In addition, σ-metalation of a TX molecule with a Pt(II) fragment was shown to significantly enhance its phosphorescence, compared to the parent TX molecule.^48^ Yet, to the best of our knowledge, **L1** represents so far the only example of a chelating ligand with a TX-based backbone and therefore offers a perfect opportunity to study radiative charge-transfer interactions between TX and emissive metal fragments. Here once again dinuclear Cu(I) systems come into mind in light of the multiple examples of luminescent Cu(I) halide dimers,^49−51^ along with higher [CuX]_*n*_ clusters.^52−54^ In addition to the possibility of stabilizing such luminescent Cu(I) halide dimers or higher aggregates by **L1**, the rigid nature of the TX backbone itself is also expected to suppress radiationless deactivation pathways, leading to higher quantum yields,^55^ as was shown for some luminescent Cu(I) complexes.

In this work, we show that, unlike the monomeric mono- and binuclear complexes that we previously observed in the Pd(II) system, Cu(I) halide **L1** exclusively forms dimeric complexes containing solvent-bridged dinuclear [Cu_2_]^2+^ cores or tri- and tetranuclear copper halide clusters. The bis-cationic dicopper core exhibited highly Lewis acidic character, being capable of chloride ion abstraction from chloroform, and therefore might prove useful for small-molecule activation via cooperative bimetallic catalysis. In addition, we provide experimental and theoretical evidence of radiative charge-transfer interactions involving the TX backbone of ligand **L1** and luminescent copper halide clusters that it stabilizes, with one of the resulting complexes exhibiting a considerable room temperature phosphorescence (Φ = 10%). This paper summarizes our endeavors in studying the structural and photophysical properties of these novel compounds.

## Results and Discussion

### Synthesis of Cu(I) Halide Complexes **1**–**3** with Tri- and Tetranuclear Cores

**Scheme 1:**
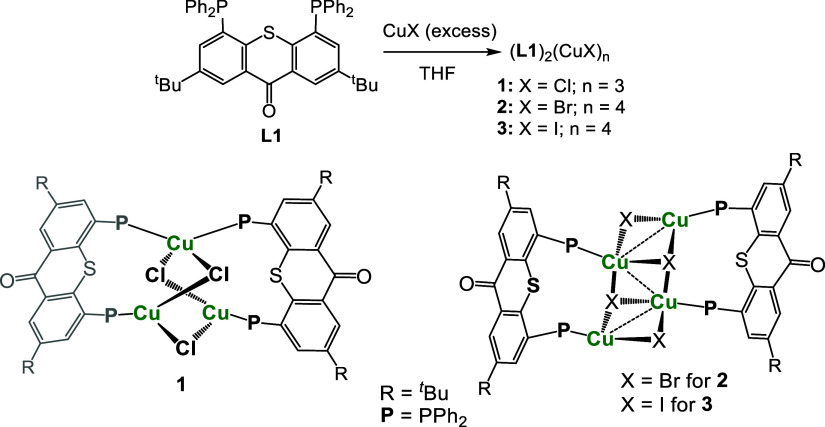
Synthesis of Cu(I) Halide Complexes **1**–**3** with Tri- and Tetranuclear Cores

We began our investigation by exploring the reactions of ligand **L1** with Cu(I) halides (Scheme 1). The addition of this ligand to a stirred suspension of 1 equiv of CuX (X = Cl, Br, I) resulted in a complex mixture of products, according to multiple signals observed in ^31^P NMR of the resulting solutions. However, the addition of a second 1 equiv of CuX resulted in a clean formation of single coordination products ([(**L1**)_2_Cu_3_(μ_1_-Cl)_3_] (**1**), [(**L1**)_2_Cu_4_(μ_3_-Br)_2_(μ-Br)_2_] (**2**), or [(**L1**)_2_Cu_4_(μ_3_-I)_2_(μ-I)_2_] (**3**), respectively), showing a single broad peak at about δ ≈ −12 ppm (*W*_1/2_ = 206–230 Hz), which is slightly downfield relative to the free ligand, resonating as a sharp singlet at δ = −15.4 ppm. Such a broadening of ^13^P NMR signals is commonly observed in Cu(I) phosphine complexes, which arises due to a relatively slow quadrupolar relaxation of the ^63^Cu and ^65^Cu nuclei (both being *I* = ^3^/_2_ isotopes).^56^ The ^1^H NMR spectra of those complexes showed a single set of signals corresponding to the chemically equivalent phenyl rings, indicating no bending of the TX backbone of **L1**, thus maintaining its *C*_2*v*_ symmetry. The carbonyl stretching frequencies of 1634 cm^–1^ (in **1** and **2**) or 1637 cm^–1^ (in **3**) were very close to that of the free ligand **L1** (1633 cm^–1^), further confirming the planarity of its TX backbone in these complexes (previously we have shown that TX bending manifests in a ∼50 cm^–1^ blue shift of its carbonyl stretching due a loss of conjugation with the aryl rings).^37^ An additional piece of structural information could be extracted from the ^13^C NMR spectra. Specifically, the signals of the phenyl *ipso*-carbons appeared as doublets (^1^*J*_P–C_ = 14.9 Hz) rather than virtual triplets, which is typical for most PXP (X = C, N, O, S) pincer systems.^37,57−59^ We already pointed out the *ipso*-carbon signal multiplicity as a fingerprint feature allowing one to distinguish κ^3^-P,S,P from other coordination modes of our PSP ligands in solution (see the Supporting Information of ref (37) for a detailed explanation).

**Figure 3:**
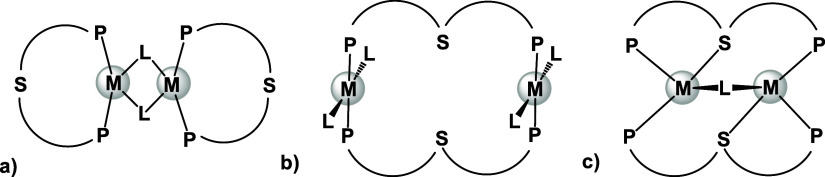
Possible coordination modes of a ligand **L1** in its dimeric complexes: κ^2^-P,P (a), μ_2_-P,P (b), and μ_2_-(κ^2^-P,S-κ^1^-P) (c).

Yet, no molecular masses corresponding to the expected dinuclear complexes analogous to that observed with Pd(II) [i.e., **L1**(CuX)_2_] could be detected by electrospray ionization high-resolution mass spectrometry (ESI-HRMS). Instead, we observed the molecular masses of *m*/*z* 1547.3077, 1593.2618, and 1638.2572 for compounds **1**, **2**, and **3**, respectively, which were consistent with the general formula of [(**L1**)_2_Cu_2_X]^+^ and suggested the presence of dimeric structures. In our previous work with PSP ligands, we already encountered two types of dimers, in which the PSP ligands adopted either a chelating κ^2^-P,P or a bridging μ_2_-P,P coordination mode^60^ (Figure 3a,b, respectively).

**Figure 4:**
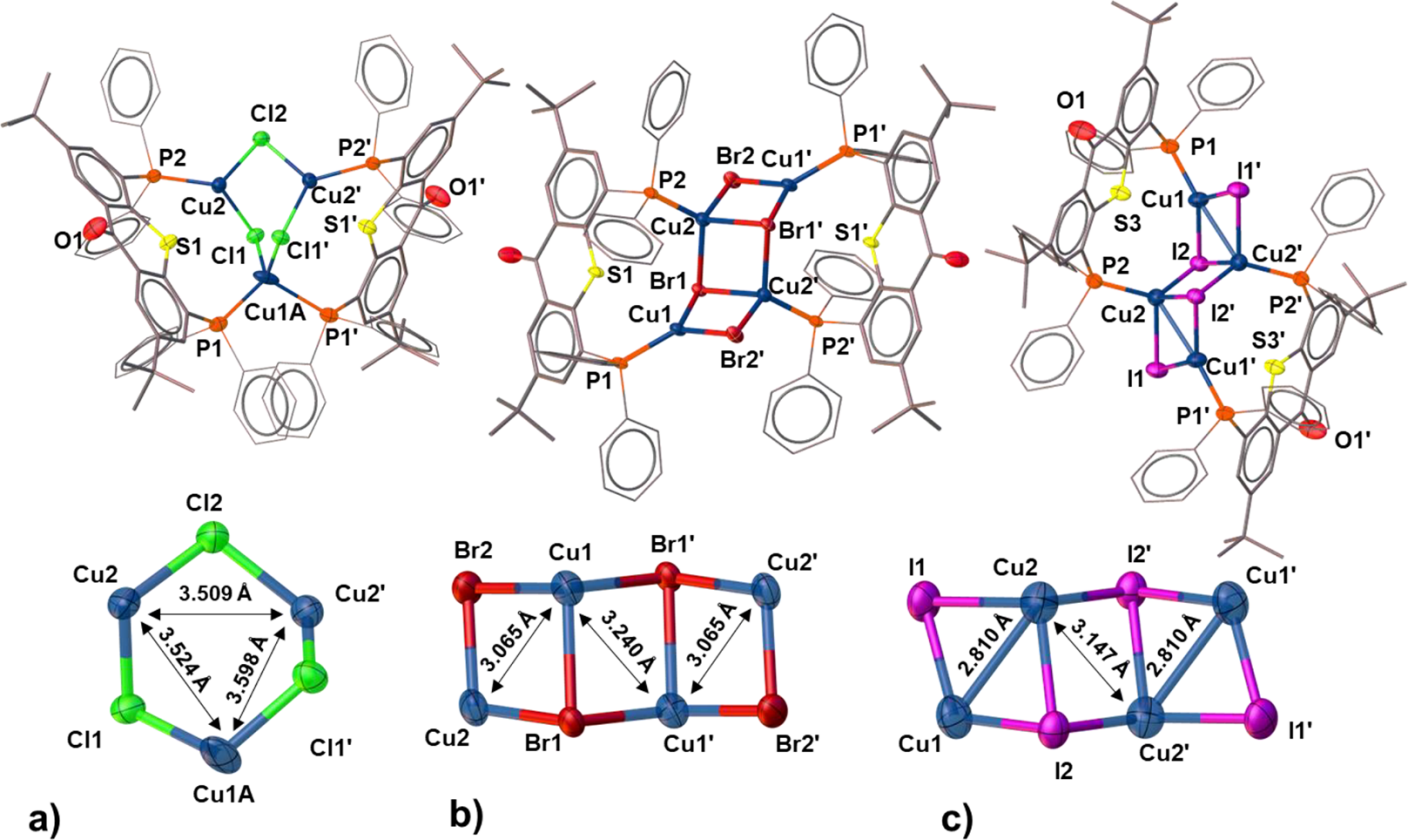
Molecular structure of complexes **1** (a), **2** (b), and **3** (c). All H atoms and solvent molecules have been omitted for the sake of clarity.

To establish the exact structures of complexes **1**–**3**, single crystals of those compounds were grown by the slow diffusion of hexane into their THF solutions. In all three cases, molecular clusters consisting of two **L1** ligands (both adopting the μ_2_-P,P bridging mode) and several CuX units were obtained. Compound **1** crystallized as a trinuclear Cu(I) complex, featuring a unique six-membered Cu_3_(μ-Cl)_3_ core (Figure 4a). To the best of our knowledge, such a Cu_3_Cl_3_ core, where one of the Cu(I) atoms adopts a distorted tetrahedral geometry, while the other two acquire a trigonal-planar geometry, is quite unusual.^61,62^ Within this cluster, two distinct Cu–Cu distances were observed (Cu1–Cu2 = Cu1–Cu2′ = 3.740 Å and Cu2–Cu2′ = 3.509 Å), both too long for any Cu–Cu interaction.

Unlike **1**, compounds **2** and **3** exhibited stairstep-shaped tetranuclear Cu_4_X_4_ cores, in which tri- and tetracoordinated Cu centers are interconnected by halide ions acquiring μ_2_- and μ_3_-bridging modes. Such a geometry of Cu_4_X_4_ clusters has been observed in the past^63−66^ and was termed a *step* or *chair* conformation, in contrast to the more common *cubane* conformation.^53,54,67^ The skewed steplike^68^ structure of those clusters results in two relatively short Cu–Cu distances, corresponding to the diagonals of rectangular Cu_2_X_2_ cells (Figure 4b,c). Although compounds **2** and **3** share the same atom connectivity within their [CuX]_4_ cores, the [CuI]_4_ cluster appears more skewed, presumably due to the larger size of the iodide ions compared to the bromide ions, which pushes them further apart (0.25 Å on av.). This, in turn, allows a shortening of all Cu–Cu distances, with the shortest one dropping below 3 Å to only Cu1–Cu2′ = Cu1′–Cu2 = 2.810 Å. Thus, considering the sum of their van der Waals radii (2.80 Å),^69^ complex **3** features Cu–Cu distances falling on the verge of cuprophilic interactions (2.4–2.8 Å).^70^ Importantly, in all three structures, the TX backbone of **L1** is nearly planar, with bending angles (i.e., the dihedral angle between the aromatic rings of TX) of only 172.4–175.6° and no close contact between any of the Cu centers and the S atom of TX.

### Synthesis and Reactivity of Bis-Cationic Dinuclear Complexes

**Scheme 2:**
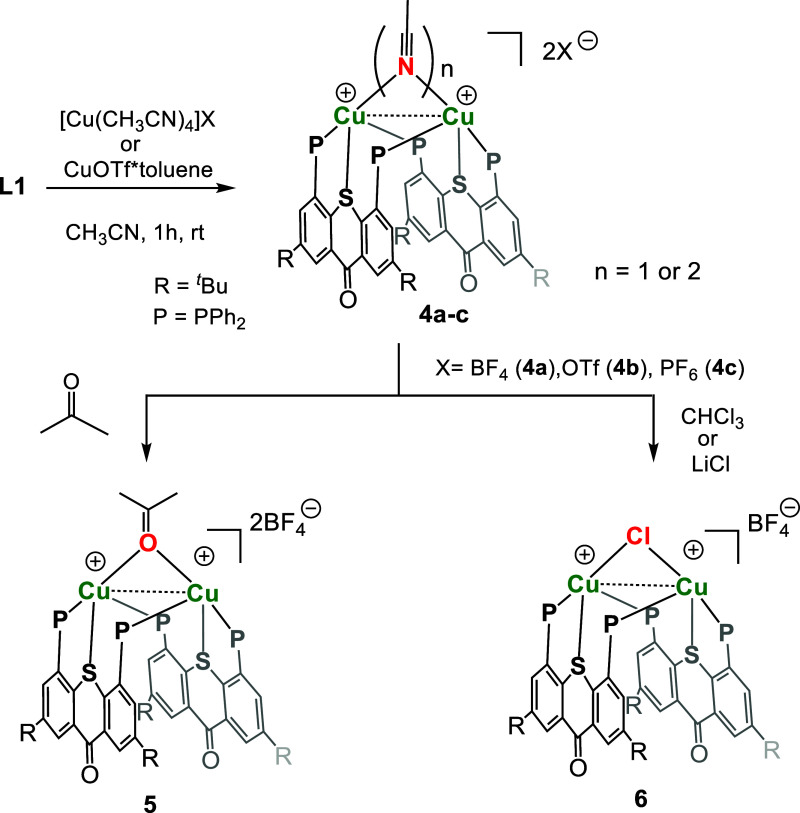
Synthesis of the Cationic Binuclear Copper Complexes **4**–**6**

While polynuclear copper halide complexes **1**–**3** were found to contain several relatively close Cu–Cu contacts, these systems are fully coordinatively saturated and therefore poorly applicable for cooperative bimetallic catalysis. Therefore, to explore the possibility of forming analogous halide-free dimeric structures, we switched to the [Cu(CH_3_CN)_4_]BF_4_ precursor. This time a ^31^P NMR spectrum recorded 1 h after its addition to **L1** exhibited a single broad peak at δ = −13.2 ppm (*W*_1/2_ = 212 Hz) consistent with the formation of a single product (**4**) that was isolated and characterized by multinuclear NMR and IR spectroscopy (Scheme 2). Spectroscopic data obtained for this compound closely resembled that of complexes **1**–**3**, namely, a very close chemical shift in ^31^P NMR (δ = −13.1 ppm), the same multiplicity of the phenyl *ipso*-carbon signals in ^13^C NMR (^1^*J*_P–C_ = 16.4 Hz), and a similar carbonyl stretching frequency (ν_CO_ = 1636 cm^–1^), suggesting that [(**L1**)_2_Cu_2_(NCMe)_*n*_](BF_4_)_**2**_ (**4a**; *n* = 1 or 2) possesses the same overall dimeric structure with planar TX backbone **L1** ligands.

In the absence of bridging chlorides, the vacant coordination sites between the Cu centers in **4** might be occupied either by the BF_4_ anion(s), for instance, in a bridging μ_2_-(F,F) mode,^71−73^ or by the MeCN solvent molecule(s).^13,32,74^ To rule out the possibility of BF_4_ coordination, we repeated the reaction of **L1** with other halide-free Cu(I) precursors, such as CuOTf*PhMe or [Cu(CH_3_CN)_4_](PF_6_)_2_ The resulting complexes [(**L1**)_2_Cu_2_(NCMe)_*n*_](OTf)_2_ (**4b**) and [(**L1**)_2_Cu_2_(NCMe)_*n*_](PF_6_)_2_ (**4c**) showed ^31^P and ^1^H NMR spectra nearly identical with those of **4a** (Figures S17 and S18). Furthermore, in all cases, ^19^F NMR showed only the characteristic signals of free anions, strongly suggesting the presence of MeCN ligands within the coordination sphere of the Cu centers.

Unfortunately, complex **4a** was unstable in all deuterated solvents except for MeCN-*d*_3_, precluding the direct identification of the coordinated MeCN molecules by NMR. Yet, the ^1^H NMR spectrum of **4a** in MeCN-*d*_3_ (Figure S10) exhibited a sharp singlet at δ = 1.96 ppm, integrating as 6H relative to the **L1** signals (i.e., 2 MeCN molecules per dimer). It is possible that this signal with the chemical shift of a free MeCN solvent originated from the weakly coordinated MeCN molecules liberated into solution after exchanging with their deuterated isotopologues. To further confirm this assumption, we performed a thermogravimetric analysis (TGA) of complex **4a**, which showed a clear desolvation weight loss of 6.4%, attributable to ca. 2.5 MeCN molecules per dimer (Figure S33).

**Figure 5:**
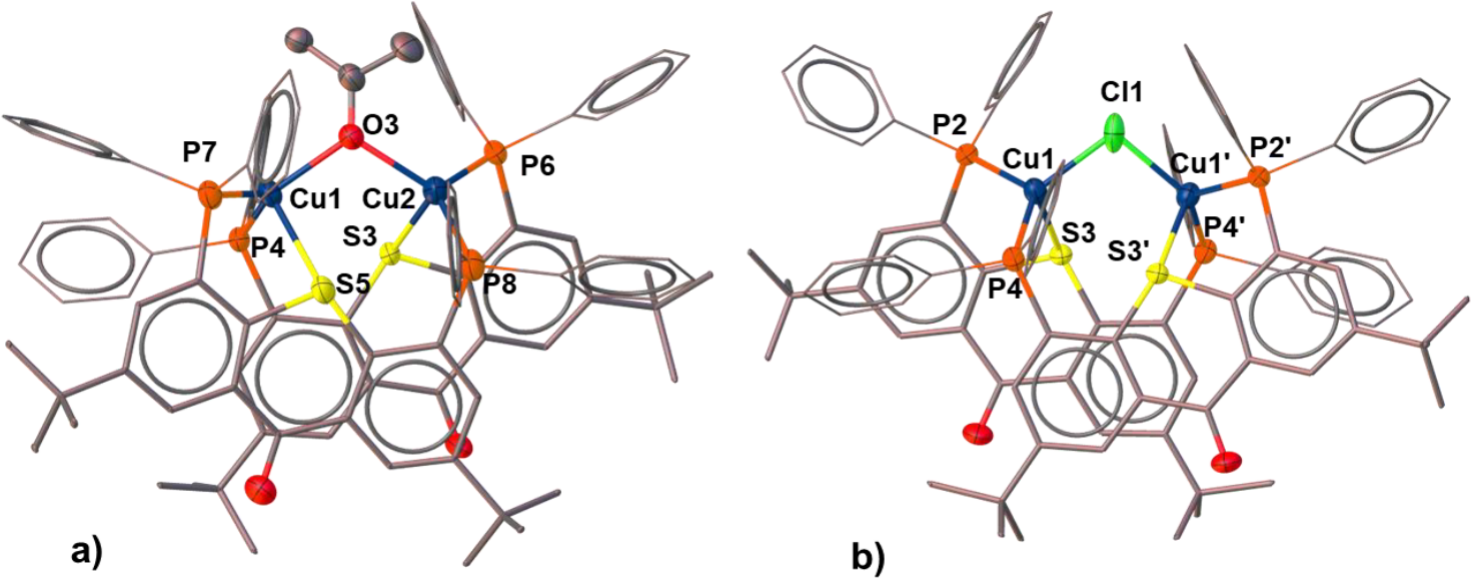
Molecular structure of complexes **5** (a) and **6** (b). H atoms, as well as BF_4_^–^ counteranion and solvent molecules, are omitted for clarity.

Despite considerable efforts, no X-ray diffraction (XRD)-quality crystals of complex **4a** or its anion-exchanged analogues could be grown from their MeCN solutions. However, switching the solvent to acetone results in the formation of yellowish crystals suitable for XRD analysis. This revealed a dimeric complex with a 1:1 L/M stoichiometry, featuring an acetone-bridged bis-cationic dicopper(I) core with a relatively short Cu–Cu distance of 3.340 Å (Figure 5a). Like in compounds **1**–**3**, the **L1** ligands in this dimer are planar, but here, their TX backbones lie in a slipped-parallel manner with respect to one another, which allows their edge-to-face π–π interactions with the phosphine phenyl rings. Such a close alignment of the ligands results in an unusual simultaneously chelating and bridging μ_2_-(κ^2^-P,S-κ^1^-P) binding mode, where each TX ligand binds one Cu center as a κ^2^-P,S chelate, while engaging the remaining phosphine for binding the other Cu center (Figure 3c). Thus, unlike in the other two types of dimers previously obtained with our PSP ligands (Figure 3a,b), here the central S atom participates in metal coordination, albeit a relatively weak one, with an elongated S–Cu bond of 2.608 Å.

**Figure 6:**
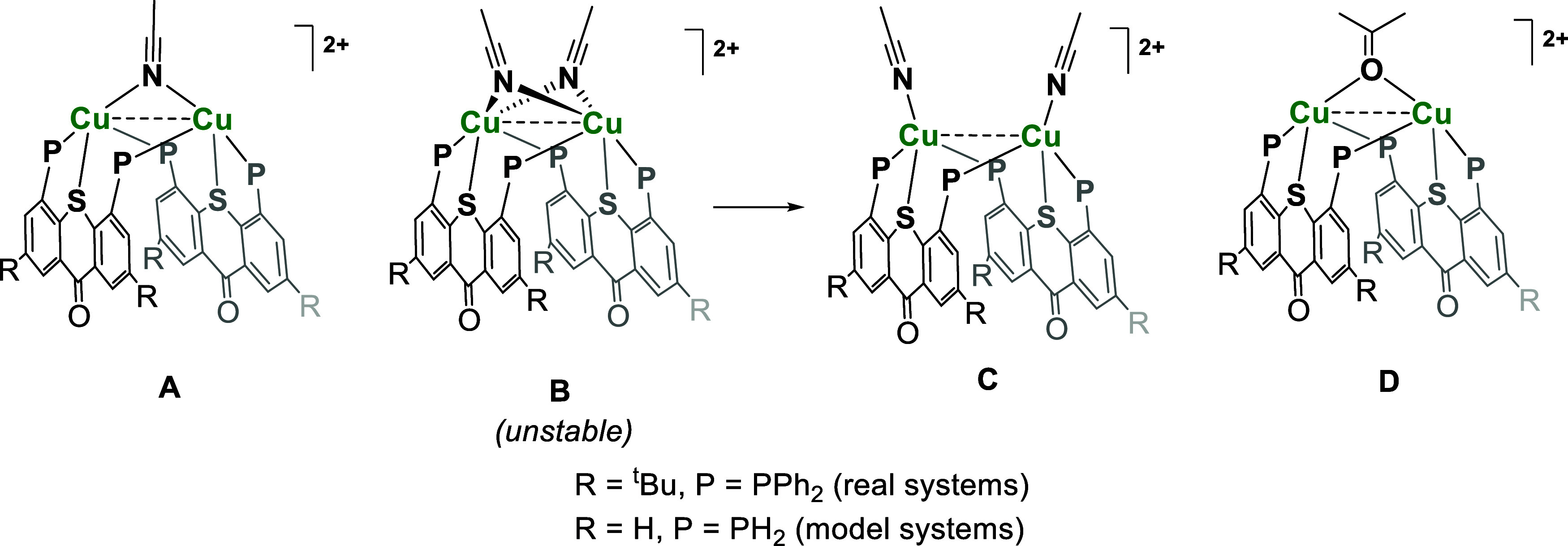
Possible coordination modes of weakly coordinated solvent molecules to a bis-cationic dicopper(I) core.

The observation of a bridging acetone molecule in [(**L1**)_2_Cu_2_(μ-acetone)](BF_4_)_2_ (**5**) raised the possibility that complexes **4a**–**4c** might feature an analogous μ-MeCN-bridged core when in an MeCN solution. The formation of such a structure in our system would be particularly intriguing in light of the unique reactivity of an isostructural dicopper(I) core demonstrated by Tilley and co-workers.^13,32^ In the absence of conclusive XRD or NMR data, to establish the most likely structure of the dicopper core of **4a**–**4c** in a MeCN solution, we resorted to computational techniques. Following related compounds discussed in the literature, we focused on three possible structures: with one bridging MeCN molecule (**A**),^32^ with two bridging MeCN molecules (**B**),^74^ and with two terminally coordinated MeCN molecules (**C**),^36,28^ as shown in Figure 6. For comparison, we also optimized the acetone-bridged structure of crystallographically characterized compound **5** (**D**). In all of these calculations, the **L1** structure was simplified by replacing the phenyl and *tert*-butyl groups with H atoms.

**Figure 7:**
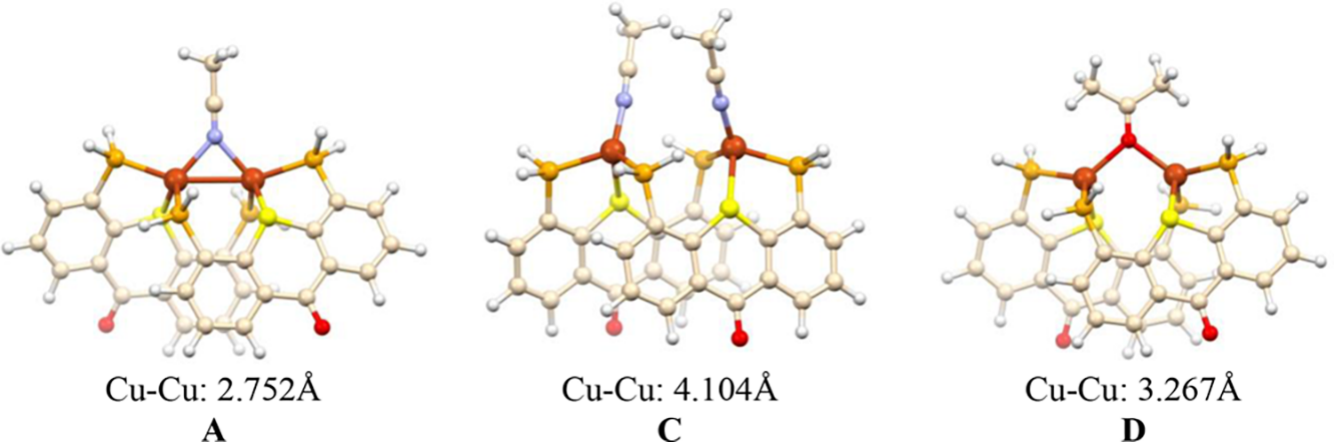
Optimized structures of model dicopper complexes **A**, **C**, and **D**.

The optimization of structure **A** resulted in a stable geometry with a short Cu–Cu contact of 2.752 Å, which is significantly closer than that found in the acetone-bridged analogue**D**, in both optimized and XRD-derived structures (3.283 and 3.267 Å, respectively). When we attempted to introduce a second bridging MeCN molecule (structure **B**), no corresponding local minimum could be found, with both MeCN molecules migrating from bridging to terminal positions in the course of the optimization process. On the other hand, the optimization of structure **C** converged to a stable geometry with a very long Cu–Cu distance of 4.104 Å, which excluded any possibility of intermetallic interaction (Figure 7).

We also considered an isodesmic transformation of **A** into **C** by the addition of a single MeCN molecule (eq 1), which was found to be only slightly exergonic (Δ*G*° = −0.45 kcal/mol). In such a case, even though in the solid-state complexes **4a**–**4c** most likely exhibit the bis-MeCN structure **C**, as suggested by the number of MeCN equivalents detected by ^1^H NMR and TGA, in solution they might exist in an equilibrium with the mono-MeCN species **A**. Because the Cu–Cu distances in **A** and **C** are quite different, the dicopper core of **4a**–**4c** appears “breathing” and therefore capable of adapting to various geometric requirements of incoming substrate molecules if applied in catalysis.
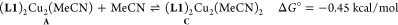
1

**Figure 8:**
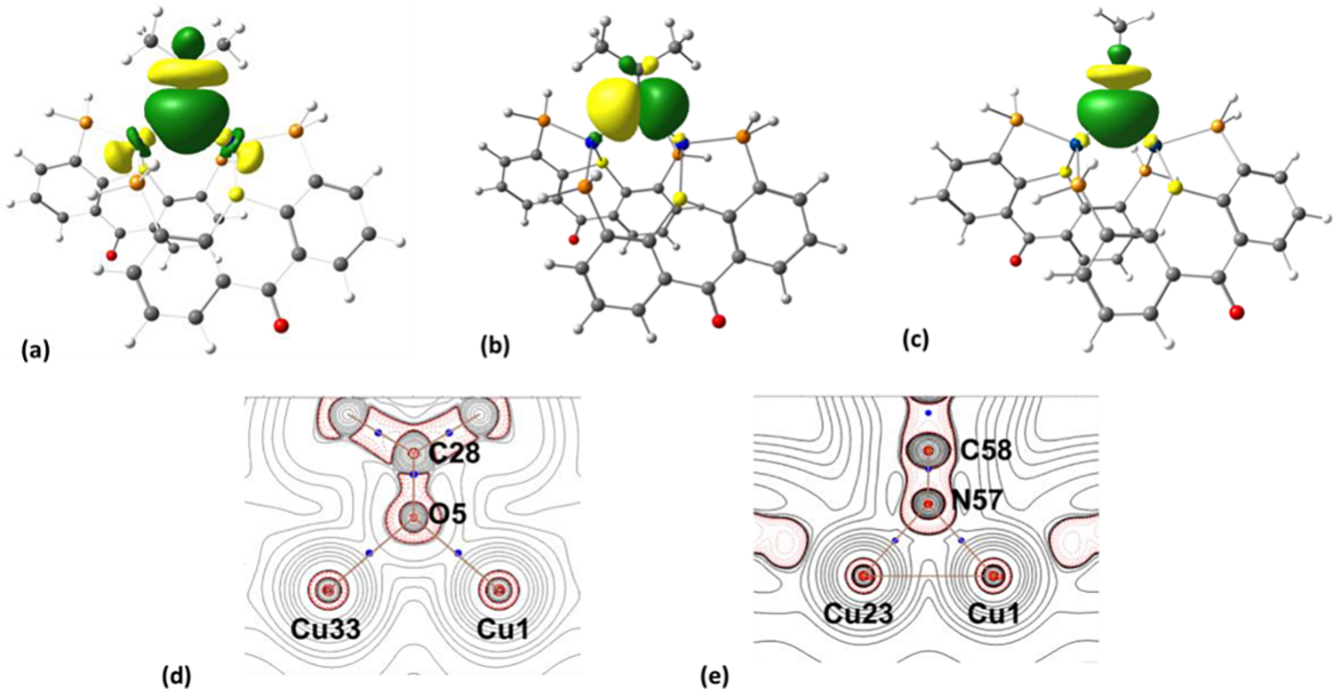
NBOs of the coordination interactions between a dicopper center and acetone in model complex **D** (a and b) and between dicopper and MeCN complex **A** (c) and contour-line maps of the electron density Laplacian in the Cu–O–Cu plane in complex **D** (d) and in the Cu–N–Cu plane in complex **A** (e).

Intrigued by the difference in Cu–Cu distances within the Me_2_CO- and MeCN-bridged cores in **D** and **A**, we undertook a theoretical investigation of the bonding in these model complexes by means of NBO and QTAIM methods (see Tables S3–S5 for the full summary of the results). According to the NBO analysis (Tables S3 and S4), the bonding in both solvent-bridged dicopper cores consists of donor–acceptor interactions between the vacant Cu orbitals of predominantly s character and the heteroatom (O or N) lone pairs. In complex **D**, both O lone pairs of the acetone molecule—an approximately sp-hybridized one, lying perpendicular to the Cu–Cu axis (Figure 8a), and another one of a pure p character, lying parallel to it (Figure 8b)—are involved in dative interactions with the two Cu centers. Similarly, in complex **A**, the MeCN molecule engages its sole sp-hybridized N lone pair in a simultaneous interaction with both Cu centers (Figure 8c), thus forming a class II μ-L 3c-2e bond^75^ similar to those reported for other nitrile- and isonitrile-bridged dicopper systems.^13^ Interestingly, even though in complex **A** only one electron pair participates in bonding, its interaction with each Cu center is even slightly stronger than the combined interaction of both acetone lone pairs in **D** (*E*^(2)^_(av)_ = 24.9 vs 21.4 kcal/mol, respectively).

The same conclusions could also be derived from the QTAIM topological analysis (Table S5), which detected bond critical points (BCPs) between the bridging heteroatoms and each of the Cu centers in both model complexes (Figure 8d,e). The low electron densities and positive Laplacian values at those BCPs (ρ_b (av)_ = 0.059 and ∇^2^ρ_(av)_ = +0.295 au for **D** and ρ_b (av)_ = 0.070 and ∇^2^ρ_(av)_ = +0.300 au for **A**) are characteristic of closed-shell interactions, in this case, dative bonding.^76^ Most importantly, despite a relatively short Cu–Cu distance in **A**, compared to **D**, no significant orbital interactions between the two Cu centers in the former model complex were identified by NBO, and, accordingly, the QTAIM analysis detected no BCPs between them. Those findings rule out any cuprophilic interactions in **D**, in contrast to a related μ-MeCN dicopper(I) complex reported by Davenport and Tilley.^13^ In this respect, our putative MeCN-bridged bis-cationic dicopper core is similar to a carbene-bridged one reported by Broere et al.,^77^ where, in spite of an even closer Cu–Cu contact of only 2.36 Å, no cuprophilic interactions were found as well.

Due to its coordinatively unsaturated nature, the reactivity of the bis-cationic dicopper core in **4a** spreads beyond simple exchange of coordinated solvent molecules, as we discovered serendipitously when attempting to grow its single crystals in chloroform, a noncoordinative solvent. To our surprise, the obtained XRD structure revealed the dimeric binuclear copper complex [(**L1**)_2_Cu_2_(μ-Cl)](BF_4_) (**6**), quite similar to **5** but with a bridging chloride ion between the two Cu centers (Figure 5b). The Cu–Cu distance in this dimeric compound is slightly shorter than that in the [CuCl]_3_ cluster-bridged complex **1** but longer than that in some reported Cl-bridged dicopper(I) complexes.^15,78^ In contrast, the Cu–Cl bonds of only 2.15 Å found in **6** are among the shortest reported for those two elements.

Literature precedents^79,80^ suggested chloroform as the most likely source of the adventitious chloride ion in this complex. To confirm that the formation of **6** from **4a** is indeed due to chloride abstraction from the solvent, the latter compound was dissolved in chloroform, which was freshly purified by passing through a pad of basic alumina to remove any traces of HCl. Indeed, the formation of compound **6**, as confirmed by a prominent molecular ion peak of *m*/*z* 1546.3216 in HRMS matching the formula of [(**L1**)_2_Cu_2_Cl]^+^, was evident by a gradual emergence of a new signal at −9.3 ppm in ^31^P NMR. The very same species also forms when the bis-cationic dimer **6** is reacted with LiCl in a nonchlorinated solvent (THF). This formation of the chloro-bridged complex provides indirect evidence for a highly Lewis acidic nature of the cationic dicopper core in **4a**, which might later prove useful for electrophilic catalysis.

### Synthesis of Monomeric Cu(I) Complexes with a Flexible Diaryl Sulfide-Based Ligand **L2**

**Scheme 3:**
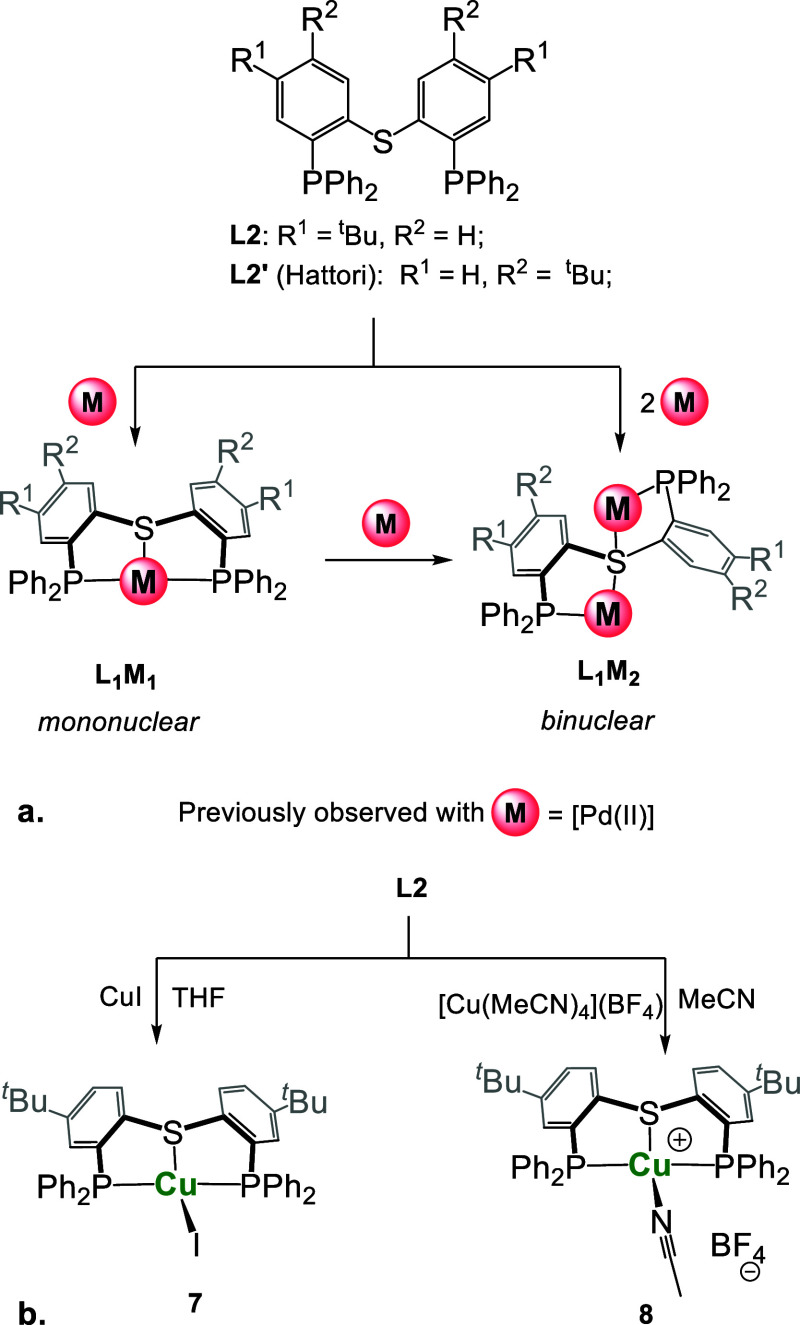
Previously Observed Coordination Modes of Ligands **L2** and **L2**′ (a) and Reactions of **L2** with Neutral and Cationic Cu(I) Precursors (b)

To further demonstrate that it is the rigid nature of the TX backbone of **L1** that determines its coordinative behavior toward Cu(I), we turned to a highly flexible PSP ligand **L2** (Scheme 3) for a comparison. This ligand was specifically chosen because, similar to **L1**, in Pd(II) systems, such diaryl sulfide-based ligands have previously been shown by us (**L2**)^37^ and others (**L2**′)^81^ to be capable of forming distinct mono- and binuclear complexes (Scheme 3a). Formation of the latter occurs by engaging both lone pairs of the central S donor in metal bonding, resulting in a doubly chelating μ-S-(κ^2^-S,P)_2_ mode, possible due to a free rotation about the S–C bonds in **L2**.

Control experiments were therefore carried out by the addition of CuI or [Cu(MeCN)_4_]BF_4_ to stirred solutions of **L2** in THF or MeCN, respectively (Scheme 3b). In both cases, the formation of single products, [(**L2**)Cu-I] (**7**) and [(**L2**)(Cu-MeCN)](BF_4_) (**8**), resonating at δ = −10.2 and −3.8 ppm, respectively, was detected by ^31^P NMR within a short time after addition, irrespective of whether 1 or 2 equiv of Cu(I) precursors was used (Figures S24 and S30). ESI-HRMS of both complexes showed the same molecular ion of *m*/*z* 729.1876 corresponding to the formula of the mononuclear species [(**L2**)Cu]^+^. Furthermore, a close look at the ^13^C NMR spectra of **7** and **8** revealed that the signals corresponding to the P-phenyl *ipso* carbons, as well as the S-aryl carbon adjacent to phosphines appeared as virtual triplets (^1^*J*_P–C_ = 14 and 30 Hz), strongly indicating the κ^3^-P,S,P coordination mode of **L2** in both complexes (Figures S25 and S32). Furthermore, in the case of complex **7**, two distinct sets of signals of the chemically inequivalent phenyl rings could be observed in the ^1^H NMR spectrum (Figure S23), clearly indicating the reduction of molecular symmetry from *C*_2*v*_ to *C*_*s*_ that ligand **L2** undergoes upon its coordination.

**Figure 9:**
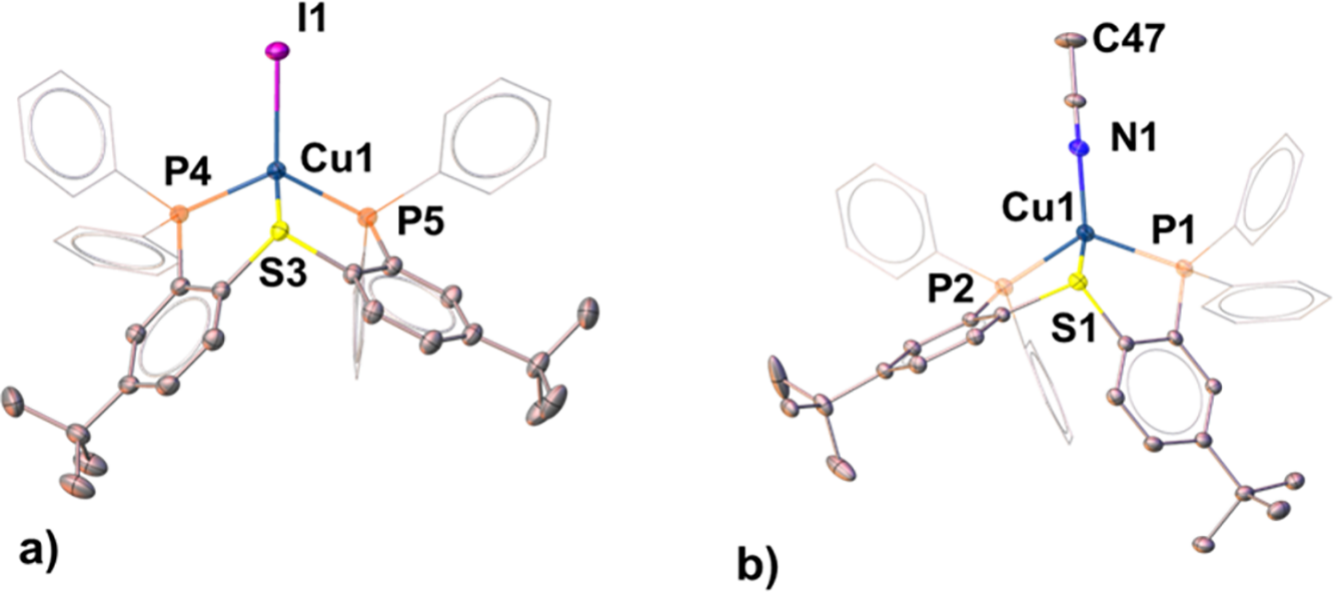
Molecular structures of complexes **7** (a) and **8** (b). H atoms, counteranions, and solvent molecules are omitted for clarity.

This coordination mode of **L2** was indeed confirmed by the XRD structures of **7** and **8**, which crystallized as mononuclear pincer complexes (Figure 9). Both neutral and cationic Cu(I) centers in these complexes exhibited a relatively rare trigonal-pyramidal geometry (τ_4_ = 0.81)^82^ defined by the phosphine and the I or N_MeCN_ atoms lying nearly coplanar with the metal (∑_angles_ = 356.78° and 355.12°, respectively), while the sulfide occupies the apical position. In fact, similarly distorted coordination spheres around Cu (τ_4_ = 0.79) were observed in the dimeric complexes **5** and **6** as well, although there the two phosphines were not originating from the same PSP ligand. The key difference between dimeric and monomeric complexes of ligands **L1** and **L2**, however, is the substantially shorter Cu–S bonds in the latter (Table 1). It is also noteworthy that, unlike the bis-cationic dicopper core in the dimeric complex **4**, the cationic Cu(I) center of **8** was entirely inert toward chloroform, which further emphasizes the unique reactivity of the former.

**Table 1:** Selected Structural Parameters of Complexes **1**–**3** and **5**–**8**

complex	bond length [Å]		bond angle [deg]		geometry index (τ_4_)	molecular symmetry
**1**	Cu1–Cu2	3.524	Cl1–Cu2–Cl2	110.80(3)		*C*_2_
	Cu2–Cu2′	3.509	P2–Cu2–Cl1	123.20(3)		
	Cu–P (av.)	2.210	Cl1–Cu1–Cl1	102.0(3)		
**2**	Cu1–Cu2	3.065	Cu1–Br1–Cu1	78.48(3)		*C*_*i*_
	Cu1–Cu1′	3.240	Br1–Cu1–Br1	101.53(3)		
	Cu–P (av.)	2.199	P1–Cu2–Br1	123.83(5)		
**3**	Cu1–Cu2′	2.810	Cu2–I2–Cu2	70.930(18)		*C*_*i*_
	Cu2–Cu2′	3.147	I2–Cu2–I2	109.071(18)		
	Cu–P (av.)	2.246	P2–Cu2–I2	108.00(3)		
**5**	Cu1–Cu2	3.340	Cu1–O3–Cu2	110.9(2)	0.79	*C*_2_
	Cu–O (av.)	2.082	P4–Cu1–S5	104.61		
	Cu–P (av.)	2.266	P6–Cu2–S3	76.90(7)		
	Cu–S (av.)	2.668				
**6**	Cu1–Cu1′	3.348	Cu1–Cl1–Cu1	102.3(3)	0.76	*C*_2_
	Cu–Cl (av.)	2.150	P2–Cu1–S3	77.13(6)		
	Cu–P (av.)	2.256	Cl1–Cu1–P4	125.58(8)		
	Cu–S (av.)	2.659				
**7**	I1–Cu2	2.5708(18)	P5–Cu2–P4	122.992(14)	0.81	*C*_*s*_
	Cu2–S3	2.4439(3)	P4–Cu2–I1	115.503(11)		
	Cu–P (av.)	2.256	P5–Cu2–I1	118.287(11)		
**8**	Cu1–S1	2.4487(5)	P2–Cu1–P1	121.65(2)	0.81	*C*_*s*_
	Cu1–N1	1.9554(19)	N1–Cu1–P2	118.43(6)		
	Cu–P (av.)	2.244	N1–Cu1–P1	115.04(6)		

**Figure 10:**
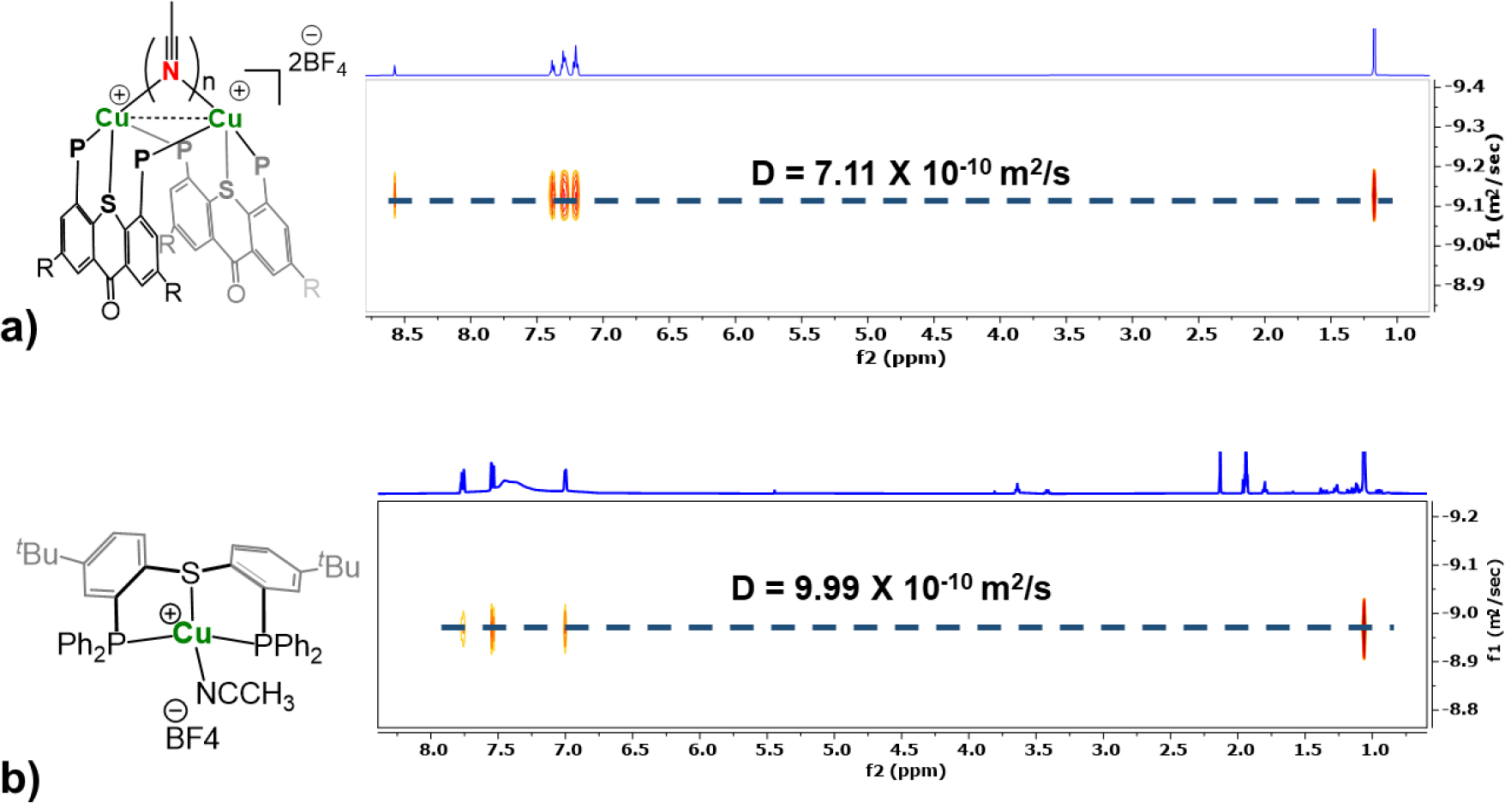
2D DOSY NMR spectra of complexes of **4a** (a) and **8** (b) in MeCN at 25 °C.

Even though **7** and **8** were isolated in the solid state as distinct mononuclear species, this might not necessarily be the case in solution, especially for complex **8**, where dimerization can easily occur upon dissociation of its loosely coordinated MeCN molecule. To clarify this point, a 2D DOSY NMR spectrum of **8** in MeCN at room temperature was compared to that of the dimeric complex **4a**, recorded under the same conditions. In both cases, only one set of contour plots was clearly visible, indicating that each of the compounds exists in solution as a single species (Figure 10). Moreover, the diffusion constant determined for **8** (*D* = 9.99 × 10^–10^ m^2^ s^–1^) was 1.405 times larger than that obtained for **4a** (*D* = 7.11 × 10^–10^ m^2^ s^–1^). The ratio between these two values is clearly higher than ^3^√2 = 1.260, the minimum theoretical *D*_monomer_/*D*_dimer_ ratio calculated for the idealized case of spherically symmetric molecules,^83^ thus confirming that the different nuclearities of complexes **4a** and **8** are maintained. This result further emphasizes the unique ability of ligand **L1** to stabilize a coordinatively unsaturated dicopper(I) core not only in the solid state but also in solution, where it might serve as a platform for a cooperative bimetallic catalysis.

### Photophysical Properties

**Figure 11:**
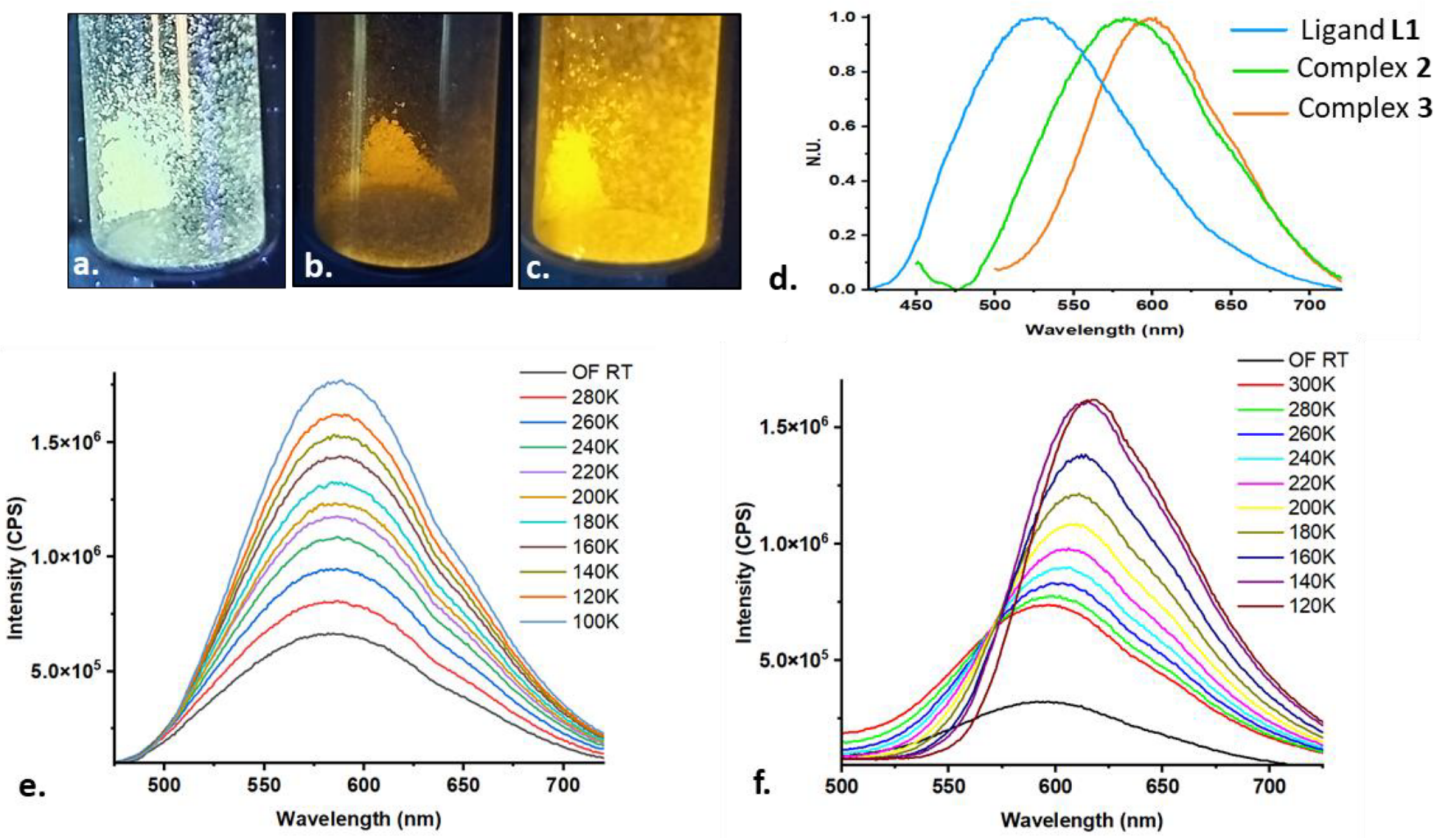
Photographs under UV light of ligand **L1** (a) and its complexes **2** and **3** (b and c, respectively), a comparison of their emission spectra at room temperature (d), and variable-temperature emission spectra of complexes **2** (e) and **3** (f) in the solid state (powder).

Before studying the luminescence properties of the copper halide complexes **1**–**3**, we first focused on ligand **L1** itself. We found that the presence of *tert*-butyl and phosphine substituents in **L1** did not quench the inherent phosphorescence of its TX core in the solid state (powder). In fact, **L1** shows a prominent emission at 525 nm (Table 2 and Figure 11a,d), somewhat blue-shifted compared to the parent TX, which emits at 560 nm.^84^

**Table 2:** Photophysical Properties of Ligand **L1** and Its Complexes in the Solid State (Powder)

compound	temp (K)	λ_ex_ (nm)	λ_em_ (nm)	τ_em_ (μs)	Φ (%)
**L1**	300	390	525	200	3
**2**	300	380	585	8	1
	100			145	n.d.^a^
**3**	300	400	590	20	10
	100		620	149	n.d.^a^

a Quantum yield measurements could only be carried out at room temperature using our equipment.

Moving to the Cu complexes, we could detect no luminescence of the **L1** complexes with the bis-cationic [Cu_2_]^2+^ and neutral [CuCl]_3_ cores (complexes **4a** and **1**). Conversely, powders of the tetranuclear complexes **2** and **3** exhibited bright-orange luminescence at room temperature, peaking at 585 and 595 nm, respectively (Table 2 and Figure 11b–d). The absolute quantum yields at room temperature were determined as 1% and 10% for complexes **2** and **3**, respectively. The corresponding lifetimes (τ_em_) of 8 and 20 μs were established by an exponential fitting of their emission decay curves (Table 2 and Figure S35).

Emission lifetimes within this time scale are usually indicative of phosphorescence due to metal-to-ligand charge transfer (^3^MLCT), halide-metal-to-ligand charge transfer (^3^XMLCT), and intra-ligand charge transfer (^3^ILCT) processes. For instance, Chen and co-workers^68^ reported similar dimeric complexes of carbazole-based PNP ligands with a *stairstep* [CuBr]_4_ core similar to the one presented here, which exhibited a yellow emission with a maximum at 549 nm and a long emission lifetime of 171.6 μs. A comparable emission wavelength (535 nm) but a lifetime of only 4.2 μs was reported for a *cubane* [CuI]_4_ cluster supported by simple triphenylphosphine ligands. Interestingly, in stark contrast to compound **3**, a *stairstep* polymorph of the former compound was nearly nonemissive at room temperature.^52^

Upon lowering the temperature from room temperature to 100 K, the emission of both compounds **2** and **3** becomes increasingly more intense due the inhibition of nonradiative decay processes by reducing thermal vibrations (Figure 11e,f). This kinetic stabilization of the triplet excited states at lower temperatures also results in significantly longer lifetimes, growing from 8 to 145 μs and from 20 to 149 μs for **2** and **3**, respectively (Table 2 and Figure S35, insets). Furthermore, while the former complex showed no thermochromic behavior within the measured temperature range (Figure 11e), the latter exhibited a prominent bathochromic shift of ca. 30 nm of its emission maxima (Figure 11f) upon cooling to 100 K. According to the literature, such a spectral shift could originate from geometrical changes within the [CuI]_4_ cluster, occurring upon cooling, especially those involving intercopper distances.^52,53^ Indeed, as established by XRD, complex **3** features several shorter Cu–Cu distances than in its Br analogue, **2**, and therefore luminescence of the former might be more sensitive to minor structural changes. In addition, the significantly stronger luminescence of **3** compared to **2** can be attributed to the so-called “heavy atom effect”, i.e., stronger spin–orbit coupling in the heavier elements that enhances the radiative decay of triplet excited states by a spin-forbidden triplet–singlet transition.^85−87^

The pronounced differences in luminescence and thermochromic behavior between complexes **2** and **3** strongly suggested the direct involvement of their [CuX]_4_ clusters in the emission process. Yet, because their emission spectra and lifetimes are not so different from those of the free ligand **L1**, one could not entirely rule out the possibility that the luminescence of these complexes is due to a metal-perturbed ligand-centered (^3^MP-LC) phosphorescence.^88,89^

**Figure 12:**
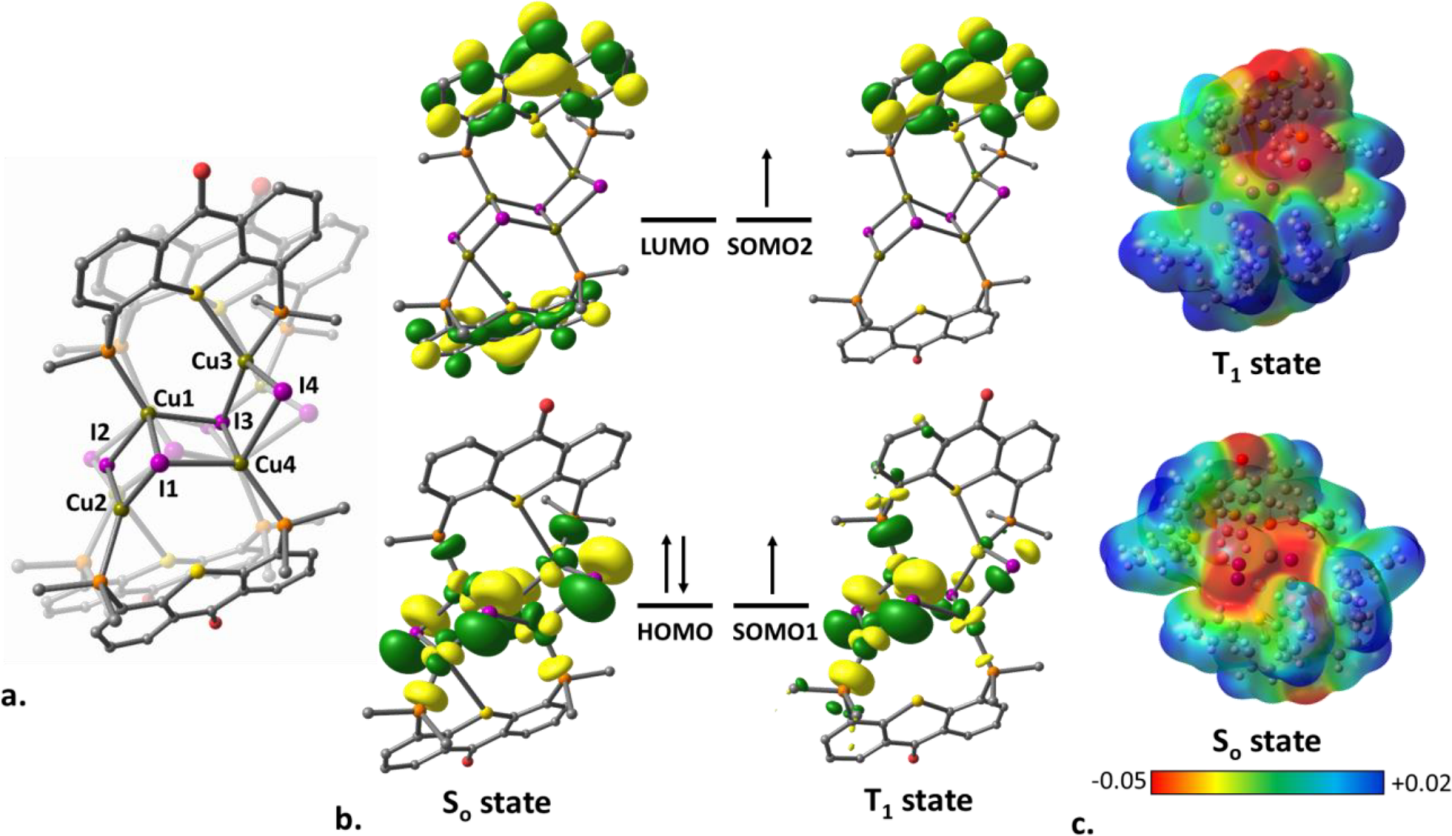
Optimized geometries of model complex 3* in the excited T_1_ and ground S_0_ electronic states overlaid (T_1_ geometry is shown at the forefront) (a), their corresponding frontier molecular orbitals (b), and ESP plots (c). H atoms and phenyl rings are omitted for clarity.

To clarify this point, we employed computational methods (DFT/TD-DFT, B3LYP/6-31G*, and LANL2DZ levels of theory). We began by optimizing the geometries of simplified versions of ligand **L1** (designated as L1*) and of its most luminescent complex, **3** (designated as 3*), at their ground and excited states (S_0_ and T_1_) (unlike in the model bis-cationic dicopper complexes **A**–**D**, here only the *tert*-butyl groups were replaced by H atoms, while the phenyl rings on the phosphines were deliberately included in calculations for a more reliable representation of the real systems). While the optimized geometries for L1* in the S_0_ and T_1_ states are nearly identical (Figure S36a and Table S6), significant structural differences occur within the (CuI)_4_ cluster in complex 3* upon its excitation from the S_0_ to T_1_ states (Figure 12a). Most importantly, in the triplet state, all Cu–Cu distances are increased by 0.41 Å on average (Table S7).

Inspection of the frontier molecular orbitals of complex 3* compared to the free ligand L1* in the T_1_ and S_0_ states revealed the different nature of luminescence in those systems. As is evident from Figure S36b, the ground-state HOMO of L1* has a significant contribution from the phosphine moieties (both P lone pairs and phenyl rings), while the LUMO is localized exclusively on the TX π system, with a large contribution from the carbonyl group. A similar difference in electron distribution also exists between the two frontier SOMO orbitals of the triplet state. Thus, the phosphorescence of **L1** can be ascribed to a phosphine-to-backbone ^3^ILCT transition. Indeed, the calculated emission wavelength of 522 nm is in excellent agreement with the experimental value of 525 nm.

In complex 3*, however, the frontier orbital localization state is quite different, with the S_0_ HOMO residing entirely on the [CuI]_4_ cluster, whereas the S_0_ LUMO is ligand-centered. Accordingly, the population of the two SOMO orbitals in the T_1_ state is consistent with a single electron transfer between the two ground-state frontier orbitals, i.e., from the [CuI]_4_ cluster to the ligand backbone (Figure 12b). This process is also evident from the corresponding electrostatic surface potential (ESP) plots (Figure 12c), clearly showing the negatively charged area on one of the ligands (designated in red) to be much more prominent in T_1_ than in S_0_. The predicted emission wavelength of 556 nm (calculated as the energy difference between the optimized triplet and singlet states with the same geometry)^52,90^ is in a reasonable agreement with the experimental value of 590 nm. These calculations clearly established that the emission of complexes **2** and **3** is indeed due to the ^3^XMLCT process and not due to ^3^ILCT of their TX ligand backbone. Finally, no luminescence was detected for the mononuclear Cu complexes **7** and **8** rather than for the flexible thioether-based ligand **L2** itself. This further emphasizes the key role of the rigid TX-based ligand **L1** in the formation of luminescent systems based on tetranuclear [CuX]_4_ clusters (X = Br, I).

## Summary

In this study, we showcased the distinctive propensity of a rigid TX-based PSP ligand **L1** to stabilize various polynuclear Cu(I) cores. The dinucleating character of ligand **L1** was even more pronounced in the Cu(I) systems described herein than in the previously studied Pd(II) systems, where it was capable of forming both mono- and binuclear complexes. In contrast to the flexible thioether-based analogue, ligand **L2**, which formed mononuclear pincer complexes, only dimeric structures of ligand **L1** with different L/M ratios were observed in all cases, both in the solid state and in solution.

With the halide-free Cu(I) precursors, this ligand forms dimeric complexes with a solvent-stabilized dicopper core, i.e., (**L1**)_2_Cu_2_(Solv)_*n*_ (Solv = MeCN or Me_2_CO). According to the DOSY measurements, these dimers stay intact in solution, and, therefore, such novel species featuring two adjacent coordinatively unsaturated Cu(I) centers might prove to be a viable platform for bimetallic catalysis. With excess copper halides, ligand **L1** was capable of stabilizing copper halide clusters, i.e., (**L1**)_2_(CuX)_*n*_ (*n* = 3 or 4), which in the case of X = I displayed room temperature phosphorescence with a quantum yield of 10%.

In conclusion, we hope that this study on a series of polynuclear Cu(I) complexes stabilized by TX-based ligands demonstrates the utility of these systems as platforms for developing cooperative bimetallic catalysts as well as their potential for preparing novel photoluminescent materials.

## Experimental Section

All air-sensitive reactions were carried out under an atmosphere of purified nitrogen in a glovebox equipped with an inert gas purifier. Tetrahydrofuran (THF), acetone (Me_2_CO), acetonitrile (MeCN), dichloromethane (DCM), and diethyl ether (Et_2_O) were purified by passing through a column of activated alumina under an inert atmosphere. Anhydrous hexane and pentane packed under inert gas over molecular sieves were used as purchased. 2,7-Di-*tert*-butyl-4,5-bis(diphenylphosphaneyl)-9*H*-thioxanthen-9-one (**L1**) and bis(4-(*tert*-butyl)-2-(diphenylphosphaneyl)phenyl)sulfane (**L2**) were prepared according to literature procedures.^11^ CuCl, CuBr, CuI, [Cu(CH_3_CN)_4_](BF_4_), [Cu(CH_3_CN)_4_](PF_6_), and CuOTf·toluene were purchased from STREM Chemicals and used as received. All chromatographic purifications were performed with prepacked SiO_2_ columns on a CombiFlash EZ-Prep instrument. High-resolution mass spectrometry (HRMS) spectra were recorded on an HR Q-TOF-MS mass instrument, using a positive-mode electrospray ionization^37^ (ESI^+^) technique (MeCN/H_2_O 80%; flow = 0.2 mL/min). NMR spectra were measured on Bruker 500 MHz Avance II and Neo spectrometers. Residual solvent peaks were used as internal standards for ^1^H and ^13^C NMR, respectively (CDCl_3_, δ 7.26/77.0 ppm; CD_2_Cl_2_, δ 5.31/53.8 ppm; CD_3_CN, δ 1.94/0.3 ppm; (CD_3_)_2_CO, δ 2.05/28.9 ppm). Other nuclei were referenced to the ^1^H reference frequency multiplied by the standard ratios: ^13^C, 0.25145020; ^19^F, 0.94094011; ^31^P, 0.40480742. NMR data are reported as follows: chemical shift, multiplicity (s = singlet, d = doublet, t = triplet, q = quartet, m = multiplet, br = broad, v = virtual), coupling constant(s), and integration. The NMR parameters were measured at 25 °C unless stated otherwise. Fourier transform infrared (FTIR) spectra of complexes **1**–**8** were measured on a Bruker AlPHA II compact spectrometer using *OPUS* software. Thermogravimetric analysis (TGA) was carried out using a Mettler Toledo TG 50 analyzer. UV/vis absorption and fluorescence spectra were recorded on an Agilent Technologies Cary 5000 UV/vis–near-IR spectrophotometer. Fluorescence measurements were performed on a Horiba Scientific Fluoromax-4 spectrofluorometer. Quantum yield measurements were carried out using a Hamamatsu C11347 PL quantum yield spectrometer. Fluorescence lifetime measurements were obtained using a Fluoro-Hub-B fluorimeter, equipped with NanoLed-390 as a light source.

### Synthetic Protocols

#### General Procedure for the Reaction of Ligand **L1** with CuX (X = Cl, Br, I)

Inside the glovebox, a solution of ligand **L1** (0.030 g, 0.05 mmol) in dry THF (2 mL) was added slowly to a stirred suspension of CuCl (0.0085 g, 0.9 mmol) in THF (2 mL). The resulting mixture was stirred for 1 h at room temperature, forming a clear solution. The solvent was dried under vacuum, and the solid residue was washed three times with Et_2_O. After removal of the solvent in a vacuum, **1** was yielded as a yellow solid (0.063 g, 88% yield). Under similar reaction conditions, the reaction of ligand **L1** (0.030 g, 0.05 mmol) with CuBr (0.012 g, 0.09 mmol) or CuI (0.016 g, 0.09 mmol) yielded **2** as a yellow solid (0.07 g, 83% yield) and **3** as a yellow solid (0.08g, 86% yield).

#### Trinuclear Complex [(**L1**)_2_Cu_3_(μ_1_-Cl)_3_] (**1**)

^1^H NMR (500 MHz, THF-*d*_8_): δ 8.61 (s, br, 4H_Ar-xanth_), 7.52 (t, *J* = 9.3 Hz, 16H_Ar_), 7.39 (t, *J* = 7.4 Hz, 8H_Ar_), 7.31 (t, *J* = 7.6 Hz, 16H_Ar_), 7.15 (d, *J* = 9.1 Hz, 4H_Ar-xanth_), 1.15 (s, 36H ^*t*^_Bu_). ^13^C NMR (126 MHz, THF-*d*_8_): δ 179.5 (s, C=O), 148.7 (s, ArC-^*t*^Bu), 134.7 (s, ArC_xanth_), 134.4 (d, *J*_pc_ = 15.6 Hz, Ar), 131.0 (s, ArC_xanth_), 130.7 (s, ArC_xanth_), 130.3 (s, ArC_xanth_), 130.2 (s, ArC_xanth_), 129.9 (s, ArC_xanth_), 128.7 (d, *J*_pc_ = 9.1 Hz, Ar), 127.1 (s, ArC_xanth_), 34.5 (s, C(CH_3_)_3_), 30.2 (s, C(CH_3_)_3_). ^31^P{^1^H} NMR (202 MHz, CDCl_3_): δ −11.9.

FTIR: ν = 1634 cm^–1^ (CO).

HRMS (ESI^+^). Calcd for [C_90_H_84_Cu_2_ClO_2_P_4_S_2_]^+^ corresponding to [M – Cu_2_Cl_3_]^+^: *m*/*z* 1547.3145. Found: *m*/*z* 1547.3152.

#### Tetranuclear Complex [(**L1**)_2_Cu_4_(μ_3_-Br)_2_(μ-Br)_2_] (**2**)

^1^H NMR (400 MHz, THF-*d*_8_): δ 8.56 (d, *J* = 2.3 Hz, 4H_Ar-xanth_), 7.58–7.43 (m, 16H_Ar_), 7.37 (ddd, *J* = 7.1, 5.2, and 1.5 Hz, 8H_Ar_), 7.30 (ddd, *J* = 9.0, 6.6, and 2.0 Hz, 16H_Ar_), 7.18 (dd, *J* = 9.0 and 2.3 Hz, 4H_Ar-xanth_), 1.14 (s, 36H ^*t*^_Bu_). ^13^C NMR (101 MHz, THF-*d*_8_): δ 179.6 (s, C=O), 148.9 (d, *J* = 4.3 Hz, ArC-^*t*^Bu), 136.9 (dd, *J*_pc_ = 18.5 and 8.1 Hz, ArC_xanth_) 135.0 (d, *J* = 2.3 Hz, ArC_xanth_), 134.3 (d, *J*_pc_ = 15.1 Hz, Ar), 130.9 (s, ArC_xanth_), 130.5 (s, ArC_xanth_), 130.5 (s, ArC_xanth_), 130.2 (s, ArC_xanth_), 130.2 (s, ArC_xanth_), 130.1 (s, ArC_xanth_), 129.7 (s, ArC_xanth_), 128.8 (d, *J* = 10.2 Hz, Ar), 127.3 (s, ArC_xanth_), 34.6 (s, C(CH_3_)_3_), 30.1 (s, C(CH_3_)_3_). ^31^P{^1^H} NMR (162 MHz, THF-*d*_8_): δ −12.1.

FTIR: ν = 1634 cm^–1^ (CO).

HRMS (ESI^+^). Calcd for [C_90_H_84_Cu_2_BrO_2_P_4_S_2_]^+^ corresponding to [M – Cu_2_Br_3_]^+^: *m*/*z* 1590.26463. Found: *m*/*z* 1590.27113.

#### Tetranuclear Complex [(**L1**)_2_Cu_4_(μ_3_-I)_2_(μ-I)_2_] (**3**)

^1^H NMR (400 MHz, CDCl_3_): δ 8.58 (dd, *J* = 2.4 and 0.7 Hz, 4H_Ar-xanth_), 7.49 (m, 16H_Ar_), 7.45–7.32 (m, 24H_Ar_), 7.30 (dd, *J* = 9.0 and 2.3 Hz, 4H_Ar-xanth_), 1.16 (s, 36H ^*t*^_Bu_). ^13^C NMR (101 MHz, THF-*d*_8_): δ 179.1 (s, C=O), 148.9 (d, *J* = 4.3 Hz, ArC-^*t*^Bu), 136.9 (dd, *J* = 18.5 and 8.1 Hz, ArC_xanth_), 135.6 (d, *J* = 2.3 Hz, ArC_xanth_), 134.3 (d, *J* = 15.2 Hz, Ar), 130.9 (s, ArC_xanth_), 130.5 (s, ArC_xanth_), 130.5 (s, ArC_xanth_), 130.2 (s, ArC_xanth_), 130.2 (s, ArC_xanth_), 130.1 (s, ArC_xanth_), 129.7 (s, ArC_xanth_), 128.8 (d, *J* = 10.1 Hz, Ar), 127.3 (s, ArC_xanth_), 34.6 (s, C(CH_3_)_3_), 30.1 (s, C(CH_3_)_3_). ^31^P{^1^H} NMR (162 MHz, CDCl_3_): δ −11.5.

FTIR: ν = 1637 cm^–1^ (CO).

HRMS (ESI^+^). Calcd for [C_90_H_84_Cu_2_IO_2_P_4_S_2_]^+^ corresponding to [M – Cu_2_I_3_]^+^: *m*/*z* 1638.25168. Found: *m*/*z* 1638.25727.

#### Bis-Cationic Binuclear Complexes [(**L1**)_2_Cu_2_(NCMe)_*n*_](X)_2_ (**4a**–**4c**; X = BF_4_, PF_6_, OTf)

Inside a glovebox, a solution of [Cu(CH_3_CN)_4_](BF_4_) (0.009 g, 0.028 mmol) in dry MeCN (1 mL) was added dropwise to a solution of ligand **L1** (0.020 g, 0.028 mmol) in MeCN (1 mL). The mixture that became a clear solution was stirred for 15 min at room temperature. The solvent was removed in vacuo, and the solid residue was washed three times with pentane and dried, yielding **4a** as a yellow solid (0.045 g, 92% yield).

Under similar reaction conditions, the reaction ligand **L1** (0.02 g, 0.028 mmol) with CuOTf·toluene (0.014 g, 0.028 mmol) or with [Cu(CH_3_CN)_4_](PF_6_) (0.01 g, 0.028 mmol) yielded solid complexes **4b** (0.047 g, 88% yield) and **4c** (0.046 g, 87% yield).

Crystallization of compound **4a** from an acetone/THF solution at −30 °C resulted in XRD-quality single crystals corresponding to compound **5**.

**4a**[BF_4_]_2_. ^1^H NMR (500 MHz, MeCN-*d*_3_): δ 8.57 (d, *J* = 2.3 Hz, 4H_Ar-xanth_), 7.37 (t, *J* = 7.4 Hz, 8H_Ar_), 7.30 (dd, *J* = 6.8 and 2.3 Hz, 4H_Ar-xanth_), 7.28–7.23 (m, 15H), 7.19 (t, *J* = 8.4 Hz, 16H_Ar_), 1.17 (s, 36H ^*t*^_Bu_). ^13^C NMR (126 MHz, MeCN-*d*_3_): δ 179.8 (s, C=O), 149.6 (d, *J* = 2.3 Hz, ArC-^*t*^Bu), 136.0 (s, ArC_xanth_), 133.4 (d, *J*_pc_ = 16.6 Hz, Ar), 132.1–131.8 (m, ArC_xanth_), 130.1 (s, ArC_xanth_), 129.4 (d, *J* = 5.7 Hz, ArC_xanth_), 128.9 (d, *J*_pc_ = 8.3 Hz, Ar), 34.5 (s, C(CH_3_)_3_), 29.9 (s, C(CH_3_)_3_). ^31^P{^1^H} NMR (202 MHz, MeCN-*d*_3_): δ −13.1. ^19^F{^1^H} NMR (376 MHz, MeCN-*d*_3_): δ −151.81 (^10^BF_4_), −151.87 (^11^BF_4_).

**4c**[OTf]_2_. ^31^P{^1^H} NMR (202 MHz, MeCN-*d*_3_): δ −13.1. ^19^F{^1^H} NMR (376 MHz, MeCN-*d*_3_): δ −78.9.

**4b**[PF_6_]_2_. ^31^P{^1^H} NMR (202 MHz, MeCN-*d*_3_): δ −13.1, 144.6 (septet, ^1^*J*_P–F_ = 707 Hz). ^19^F{^1^H} NMR (376 MHz, MeCN-*d*_3_): δ −72.9.

FTIR: ν = 1638 cm^–1^ (CO).

Due to extreme sensitivity, complexes **4a**–**4c** could not be characterized by HRMS.

#### Chloride Anion Abstraction from Chloroform by Complex **4a**

In a reaction tube, complex **4a** (0.02 g, 0.01 mmol) was dissolved in CHCl_3_ (1 mL), and the resulting solution was stirred for 6 h at room temperature. Pentane (2 mL) was added to afford a yellow precipitate, and the mixture was stirred rapidly for 5 min. The precipitate was allowed to settle and filtered through a Celite plug, and the precipitate was washed with DCM. After removal of the solvent, the solid was rinsed with pentane three times (2 mL each time), and the remaining volatile compounds were removed in vacuo, affording **6** as a yellow powder (0.034 g, 72% yield). Layering pentane in a DCM mixture solution of the product afforded X-ray-quality yellow crystals of **6**.

Complex **6** could also be obtained by mixing complex **4a** (0.050 g, 0.028 mmol) and LiCl (1.2 mg, 0.028 mmol) in THF (2 mL) stirring the reaction mixture for 1 h at room temperature.

#### Cationic Dinuclear Complex [(**L1**)_2_Cu_2_(μ-Cl)](BF_4_) (**6**)

^1^H NMR (400 MHz, CDCl_3_): δ 8.52 (d, *J* = 2.2 Hz, 4H_Ar-xanth_), 7.33 (s, br, 8H_Ar_), 7.03 (s, br, 28H_Ar_), 6.38 (s, br, 4H_Ar-xanth_, 4H_Ar_), 1.21 (s, 36H ^*t*^_Bu_). ^13^C NMR (101 MHz, CDCl_3_): δ 177.8 (s, C=O), 150.9 (s, ArC-^*t*^Bu), 136.0 (s, ArC_xanth_), 134.6 (s, br, Ar), 132.8 (m, ArC_xanth_), 131.4 (s, ArC_xanth_), 129.2 (s, ArC_xanth_), 128.8 (s, br, Ar), 35.2 (s, C(CH_3_)_3_), 30.8 (s, C(CH_3_)_3_). ^31^P{^1^H} NMR (162 MHz, CDCl_3_): δ −9.4. ^19^F{^1^H} NMR (376 MHz, CDCl_3_): δ −152.7.

FTIR: ν = 1641 cm^–1^ (CO).

HRMS (ESI^+^). Calcd for [C_90_H_84_Cu_2_ClO_2_P_4_S_2_]^+^ corresponding to [M]^+^: *m*/*z* 1546.31600. Found: *m*/*z* 546.32165.

#### Mononuclear Complexes of Ligand **L2**

In a reaction tube, ligand **L2** (0.020 g, 0.03 mmol) and CuI (0.006 g, 0.03 mmol) were dissolved together in 2 mL of MeCN. The reaction mixture was stirred for 15 min at room temperature, after which all volatiles were removed by evaporation. The solid residue was collected by filtration, washed with pentane three times (2 mL each time), and dried under vacuum to give the product as a white solid **7** (0.022g, 88% yield).

Using the same procedure, ligand **L2** (0.02 g, 0.03 mmol) was reacted with [Cu(CH_3_CN)_4_](BF_4_) (0.009 g, 0.03 mmol), affording complex **8** as a white solid (0.021g, 88% yield).

#### Complex [(**L2**)CuI] (**7**)

^1^H NMR (500 MHz, CD_2_Cl_2_): δ 7.82 (d, *J* = 7.7 Hz, 4H_Ar-xanth_), 7.65 (dt, *J* = 8.3 and 2.4 Hz, 2H_Ar-xanth_), 7.51–7.33 (m, 8H_Ar_), 7.24 (d, *J* = 7.5 Hz, 3H_Ar_), 7.13 (t, *J* = 7.7 Hz, 4H_Ar_), 6.98 (td, *J* = 3.4 and 2.3 Hz, 2H_Ar-xanth_), 6.93–6.75 (m, 4H_Ar_), 1.07 (s, 18H ^*t*^_Bu_). ^13^C NMR (126 MHz, CD_2_Cl_2_): δ 152.4 (s, ArC-^*t*^Bu), 137.7 (vt, *J*_pc_ = 30.2 Hz, ArC_xanth_), 137.0 (t, *J*_pc_ = 35.0 Hz, ArC_xanth_), 134.5 (t, *J*_pc_ = 14.2 Hz, Ar), 134.0 (t, *J*_pc_ = 6.3 Hz, Ar), 132.7 (vt, *J*_pc_ = 14.2 Hz, Ar), 131.5 (s, ArC_xanth_), 130.2 (s, ArC_xanth_), 129.2 (s, ArC_xanth_), 128.5 (t, *J*_pc_ = 9.3 Hz, Ar), 128.2 (t, *J*_pc_ = 9.3 Hz, Ar), 127.7 (s, ArC_xanth_), 34.6 (s, C(CH_3_)_3_), 30.5 (s, C(CH_3_)_3_).

HRMS (ESI^+^). Calcd for [C_44_H_44_CuP_2_S]^+^ corresponding to [M – I]^+^: *m*/*z* 729.1935. Found: *m*/*z* 729.1877.

#### Cationic Complex [(**L2**)Cu(MeCN)](BF_4_) (**8**)

^1^H NMR (400 MHz, MeCN-*d*_3_): δ 7.78–7.76 (m, 1H_Ar-thioether_), 7.76–7.73 (m, 1H_Ar-thioether_), 7.55 (dt, *J* = 2.2 and 0.4 Hz, 1H_Ar-thioether_), 7.53 (dt, *J* = 2.3 and 0.5 Hz, 1H_Ar-thioether_), 7.52–7.06 (m, br, 20H_Ar_), 7.02–6.98 (m, 2H_Ar-thioether_), 1.06 (s, 18H^*t*^_Bu_). ^13^C NMR (101 MHz, MeCN-*d*_3_): δ 153.3 (s, ArC-^*t*^Bu), 137.0 (vt, *J* = 29.6 Hz, ArC _thioether_), 135.6 (vt, *J* = 38.4 Hz, ArC _thioether_), 134.4 (vt, *J* = 14.5 Hz, Ar), 132.9 (s, br, Ar), 131,6 (s, ArC _thioether_), 130.5 9 (s, br, Ar), 129.0 (s, br, Ar), 128.8 (s, br, Ar), 34.5 (s, C(CH_3_)_3_), 29.9 (s, C(CH_3_)_3_). ^31^P NMR (162 MHz, MeCN-*d*_3_): δ −3.8. ^19^F NMR (376 MHz, MeCN-*d*_3_): δ −151.79 (^10^BF_4_), −151.85 (^11^BF_4_).

HRMS (ESI^+^). Calcd for [C_44_H_44_CuP_2_S]^+^ corresponding to [M – MeCN]^+^: *m*/*z* 729.1935. Found: *m*/*z* 729.1877.

### Computational Methods

All DFT calculations have been performed using the *Gaussian 16* suite of programs.^91^ Molecular structures of model complexes **A**–**D** were optimized in the presence of solvent with the TPSS functional^92^ and def2TZVP basis set.^93^ Frequency calculations were performed on the optimized geometries to confirm that they are a local minimum in the potential energy surface. To account for the solvation, the polarizable continuum model was employed^94^ using MeCN for molecules **A**–**C** and Me_2_CO for molecule **D**. The natural bond order (NBO) and a second-order perturbation analysis were performed on the optimized structures using the *NBO-7.0* program.^95^ QTAIM analysis was done with the *AIMAll* program^96^ (AIMQB module) using the WFX file from *Gaussian 16* calculations. Geometry optimization of model ligand L1* and model complex 3* was performed with the UB3LYP functional using the LANL2DZ basis set, with an effective core potential employed for the I, Cu, P, and S atoms and the 6-31G* basis set for the O, C, and H atoms.^97^ In the case of complex 3*, the ground-state geometry (S_0_) was obtained from an XRD structure of complex **3** simplified by replacing the *t*-Bu groups by H atoms, optimizing their positions only. The ground-state geometry of ligand L1* was obtained by optimization of all of the atoms. Vertical electronic excitations based on (U)B3LYP-optimized geometries were computed by the time-dependent density functional theory (TD-DFT) formalism, using the conductor-like polarizable continuum model with THF as a solvent.^98,99^ A total of 50 roots were considered in the calculation. T_0_ obtains the emission spectra for complexes 3* and L1*, and the optimized geometry of the T_1_ state was obtained using the TD-DFT formalism, taking the optimized S_0_ geometry as the starting point. The emission wavelength was calculated as the energy difference between the optimized triplet state (T_1_) and a singlet state with geometry identical with that of the optimized T_1_ state.^52,90^ The XYZ coordinates of all optimized structures are given as a separate file in the Supporting Information.

## Supporting Information Available

NMR spectra, TGA, photophysical measurements, XRD parameters, and computational data (PDF)
Coordinates of all optimized structures (XYZ)

## Supplementary Material

ic4c03819_si_001.pdf

ic4c03819_si_002.xyz
