# Supplementary material for: Assembling Di- and Polynuclear Cu(I) Complexes with Rigid Thioxanthone-Based Ligands: Structures, Reactivity, and Photoluminescence
Source: Inorg Chem. 2024 Dec 16;63(52):24466–81. doi: 10.1021/acs.inorgchem.4c03819 (PMC11688670; doi:10.1021/acs.inorgchem.4c03819)
Supplement: Supplementary file 1 — ic4c03819_si_001.pdf [file ic4c03819_si_001.pdf]

Supporting Information

# Assembling Di- and Polynuclear Cu(I) Complexes with Rigid Thioxanthone-based Ligands – Structures, Reactivity and Photoluminescence

Mohammad Zafar,[†‡] Vasudevan Subramaniyan,[†‡] Kamal Uddin Ansari,[†] Hadar Yakir,[†] David Danovich,[†] Yuri Tulchinsky*[†]

[†]Institute of Chemistry, Hebrew University of Jerusalem, Jerusalem, 9190401, Israel

## Table of Contents

**1. NMR spectra** **Page no.**


| | | |
|---|---|---|
| Figure S1 | $^{1}$H NMR spectrum of **1** in THF-*d*8. | S4 |
| Figure S2 | $^{31}$P NMR spectrum of **1** in THF-*d*8 | S4 |
| Figure S3 | $^{13}$C{$^{1}$H} NMR spectrum of **2** in THF-*d*8 | S5 |
| Figure S4 | $^{1}$H NMR spectrum of **2** in THF-*d*8 | S5 |
| Figure S5 | $^{31}$P{$^{1}$H} NMR spectrum of **2** in THF-*d*8. | S6 |
| Figure S6 | $^{13}$C{$^{1}$H} NMR spectrum of **2** in THF-*d*8 | S6 |
| Figure S7 | $^{1}$H NMR spectrum of the **3** in $CDCl_3$ | S7 |
| Figure S8 | $^{31}$P{$^{1}$H} NMR spectrum of **3** in $CDCl_3$. | S7 |
| Figure S9 | $^{13}$C{$^{1}$H} NMR spectrum of **3** in THF-*d*8 | S8 |
| Figure S10 | $^{1}$H NMR spectrum of **4a** in $CD_3CN$ | S8 |
| Figure S11 | COSY NMR of **4a** in $CD_3CN$ | S9 |
| Figure S12. | HSQC NMR of **4a** in $CD_3CN$ | S9 |
| Figure S13 | HMBC NMR of **4a** in $CD_3CN$ | S10 |
| Figure S14 | $^{31}$P{$^{1}$H} NMR spectrum of **4a** in $CD_3CN$ | S10 |
| Figure S15 | $^{13}$C{$^{1}$H} NMR spectrum of **4a** in $CD_3CN$ | S11 |
| Figure S16 | $^{19}$F{$^{1}$H} NMR spectrum of **4a** in $CD_3CN$ | S11 |
| Figure S17 | Stacked $^{1}$H NMR spectra of the **4a**, **4b** and **4c** in $CD_3CN$ | S12 |
| Figure S18 | Stacked $^{31}$P NMR spectra of the **4a**, **4b** and **4c** in $CD_3CN$ | S12 |
| Figure S19 | $^{1}$H NMR spectrum of **6** in $CDCl_3$ | S13 |
| Figure S20 | $^{31}$P{$^{1}$H} NMR spectrum of **6** in $CDCl_3$ | S13 |
| Figure S21 | $^{19}$F{$^{1}$H} NMR spectrum of **6** in $CDCl_3$ | S14 |
| Figure S22 | $^{13}$C{$^{1}$H} NMR spectrum of **6** in $CDCl_3$ | S14 |
| Figure S23 | $^{1}$H NMR spectrum of **7** in $CD_2Cl_2$ | S15 |
| Figure S24 | $^{31}$P{$^{1}$H} NMR spectrum of **7** in $CD_2Cl_2$ | S15 |
| Figure S25 | $^{13}$C{$^{1}$H} NMR spectrum of **7** in $CD_2Cl_2$ | S16 |
| Figure S26 | $^{1}$H NMR spectrum of **8** in $CD_3CN$ | S16 |
| Figure S27 | COSY NMR of **8** in $CD_3CN$ | S17 |
| Figure S28. | HSQC NMR of **8** in $CD_3CN$ | S17 |
| Figure S29 | HMBC NMR of **8** in $CD_3CN$ | S18 |
| Figure S30 | $^{31}$P{$^{1}$H} NMR spectrum of **8** in $CD_3CN$ | S18 |
| Figure S31 | $^{19}$F{$^{1}$H} NMR spectrum of **8** in $CD_3CN$ | S19 |
| Figure S32 | $^{13}$C{$^{1}$H} NMR spectrum of **8** in $CD_3CN$ | S19 |

2. **Thermogravimetric analysis**

Figure S33 TGA of complex **4a**. S20

3. **Photophysical measurements**

Figure S34 UV-Vis absorption spectra of **L1**, **2** and **3** in THF S21

Figure S35 Phosphorescence decay of complexes **2** and **3** S22

4. **Crystallography**

**4.1 General crystallographic and refinement details** S23

**4.2 Crystallographic parameters**

Table S1 Crystallographic parameters of complexes **1-3** S24

Table S2 Crystallographic parameters of complexes **5-8** S25

5. **Computational data**

Table S3 NBOs participating in the Cu-L bonding (L = MeCN or $Me_2CO$) S26

Table S4 Second order perturbation energies of the Cu-L orbital interactions (L = MeCN or $Me_2CO$). S26

Table S5 QTAIM analysis results - XYZ coordinates of selected atoms and bond critical points (BCPs), along with electron densities and electron densities Laplacian values at those BCPs S26

Table S6 Selected bond lengths of the optimized geometries of **L1*** in the $S_0$ and $T_1$ states S27

Table S7 Selected bond lengths of the optimized geometries of **3*** in the $S_0$ and $T_1$ states S27

Figure S36 Optimized geometries of the model ligand **L1*** in the $S_0$ and $T_1$ states, corresponding frontier MOs, and ESP plots S28

Figure S37 Overlay of the experimental spectra of **L1** and **3** with the calculated electronic transitions of **L1*** and **3*** S29

Table S8 Electronic transitions of ligand **L1** (**L1***) S29

Table S9 Electronic transitions of complex **3** (**3***) S29

Figure S38 Frontier MOs of **L1*** and **3*** involved in UV-Vis transitions listed in **Tables S8-S9** S30

## 1. NMR spectra.

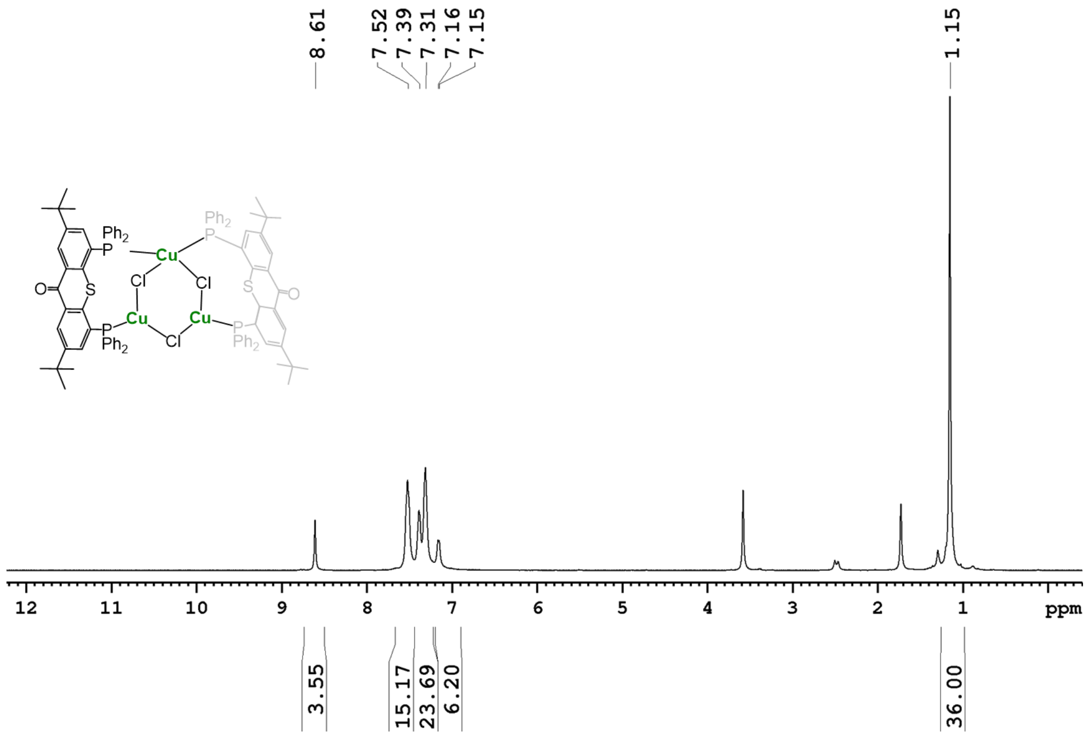


**Figure S1**. $^{1}$H NMR spectrum of **1** in THF-$d_8$ (500 MHz).

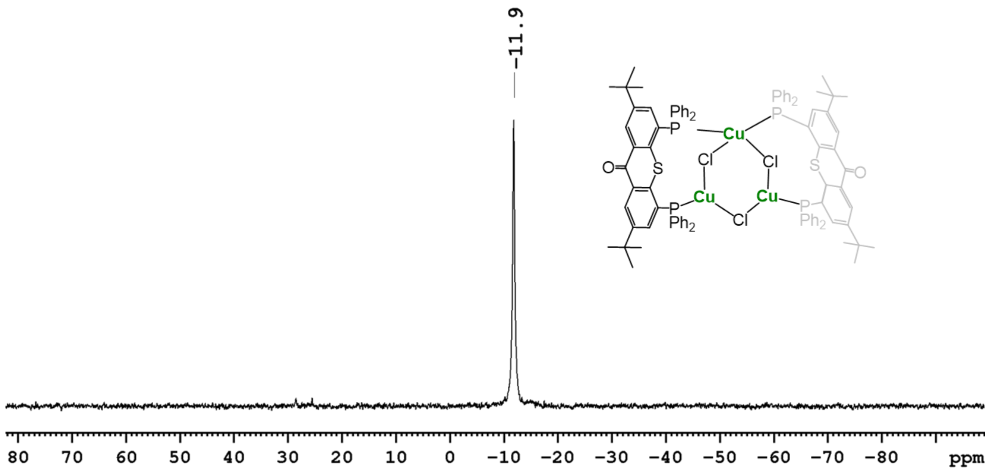


**Figure S2**. $^{31}$P NMR spectrum of **1** in THF-$d_8$ (202 MHz).

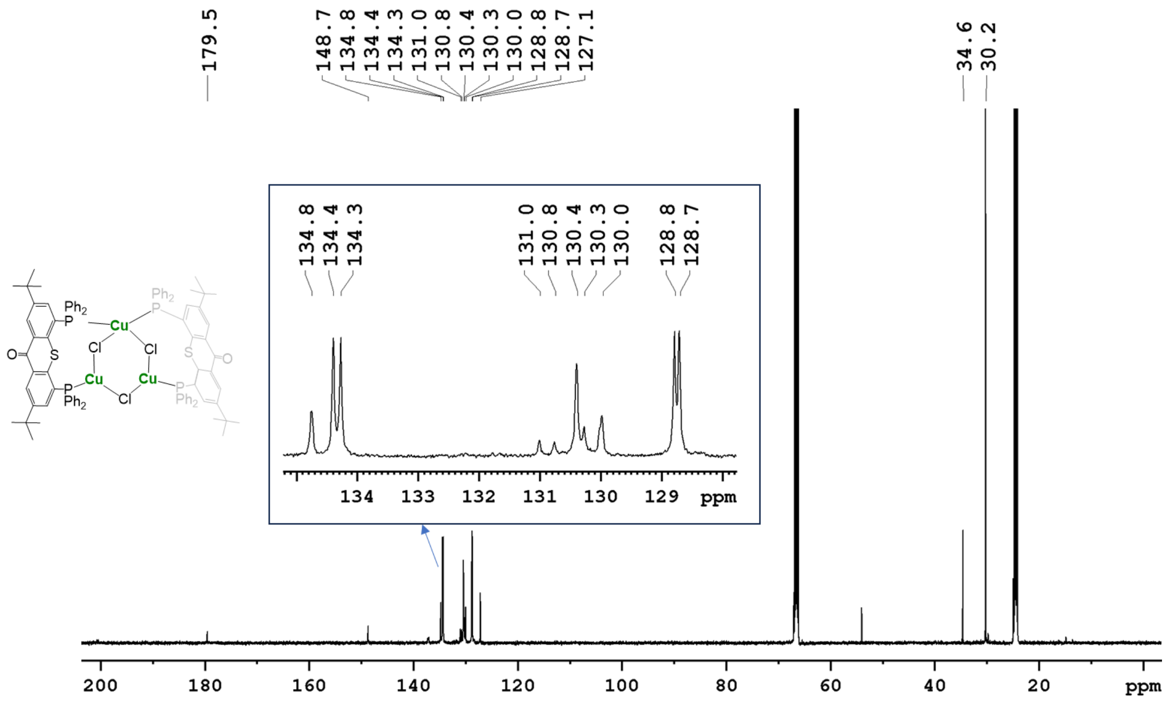


**Figure S3**. $^{13}C\{^{1}H\}$ NMR spectrum of **1** in THF-$d_8$ (126 MHz).

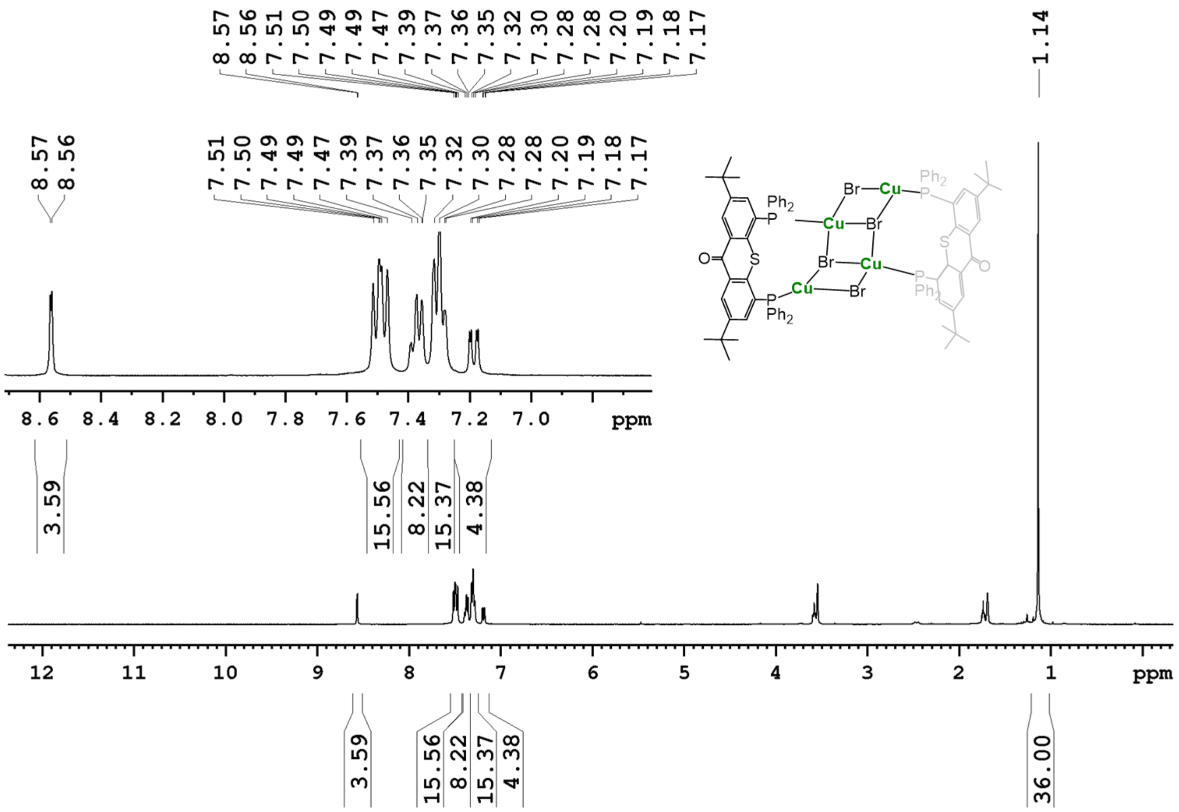


**Figure S4**. $^{1}H$ NMR spectrum of the **2** in THF-$d_8$ (400 MHz).

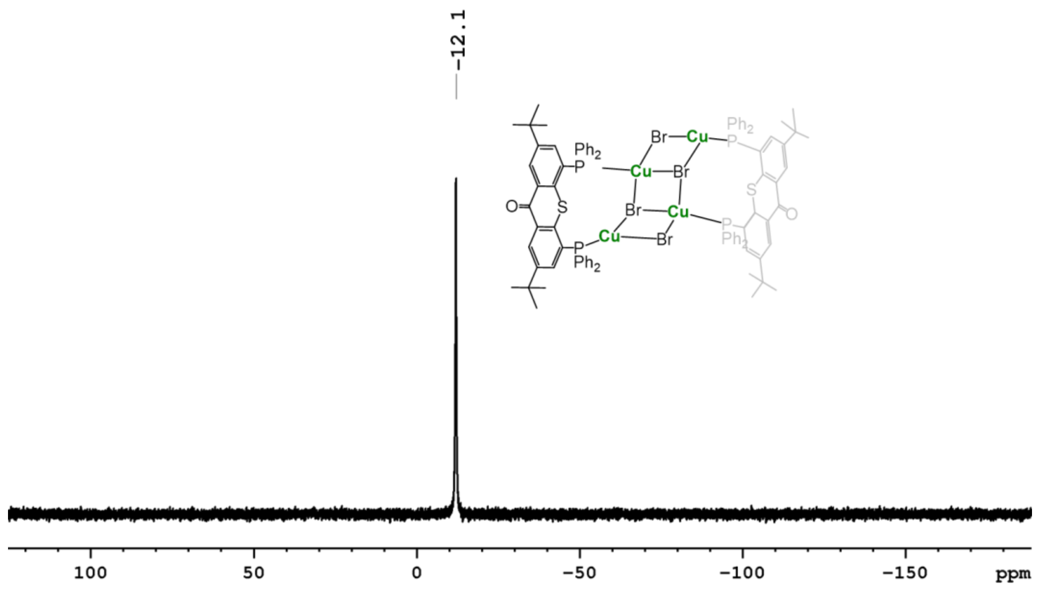


**Figure S5**. $^{31}P\{^1H\}$ NMR spectrum of the **2** in THF-$d_8$ (162 MHz).

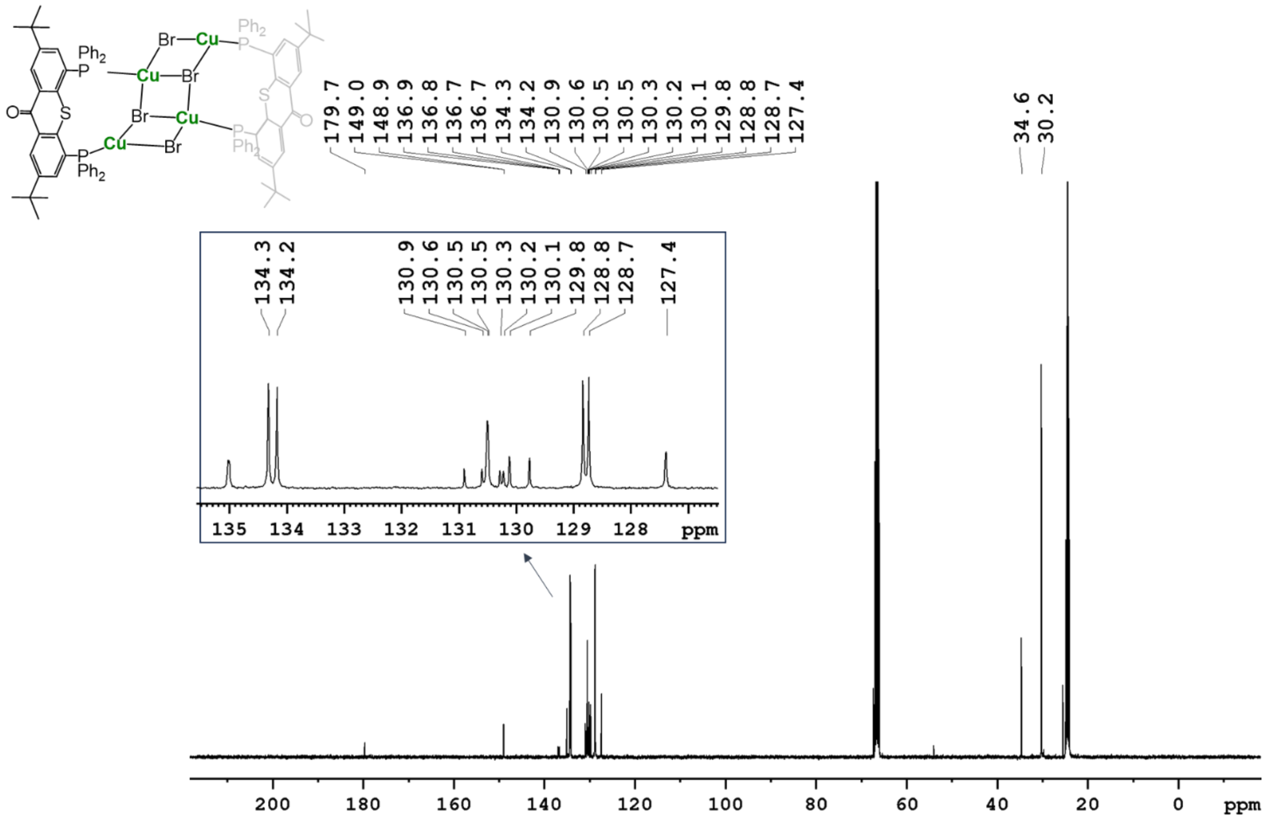


**Figure S6**. $^{13}C\{^1H\}$ NMR spectrum of **2** in THF-$d_8$ (101 MHz).

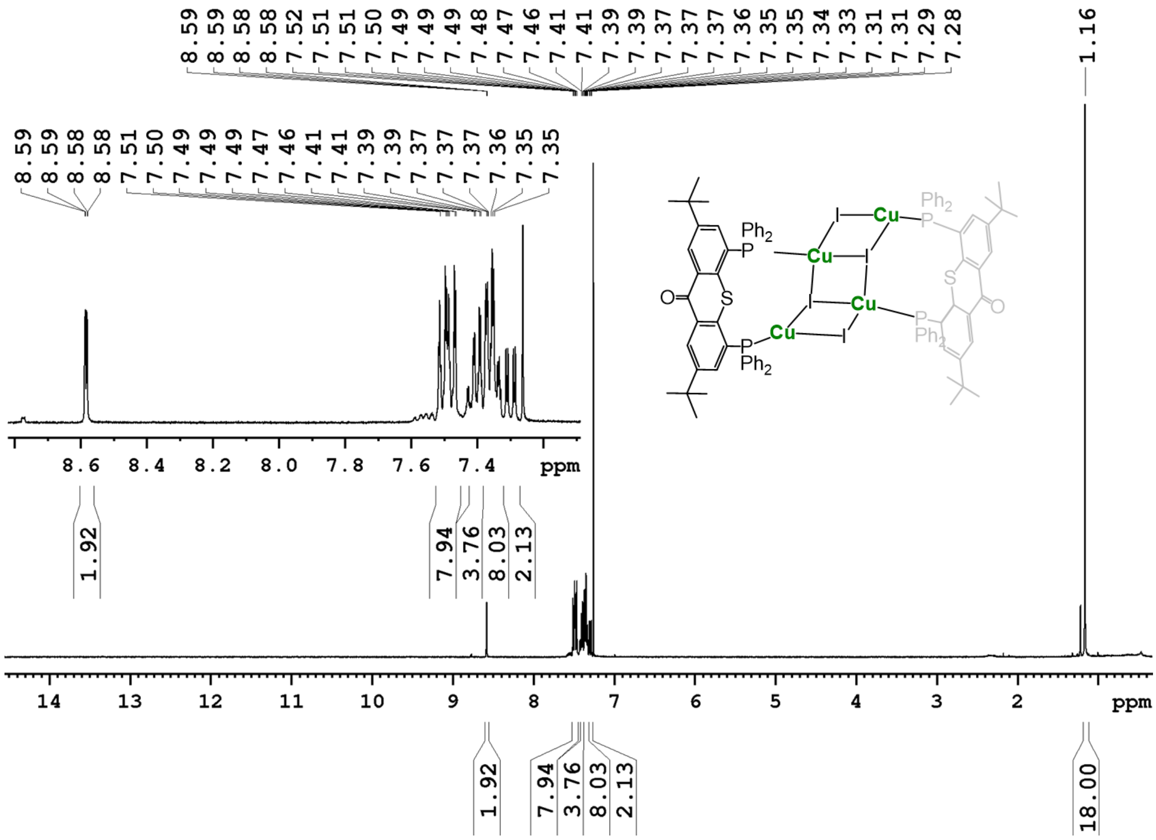

**Figure S7**. $^{1}$H NMR spectrum of the **3** in $CDCl_3$ (400 MHz).

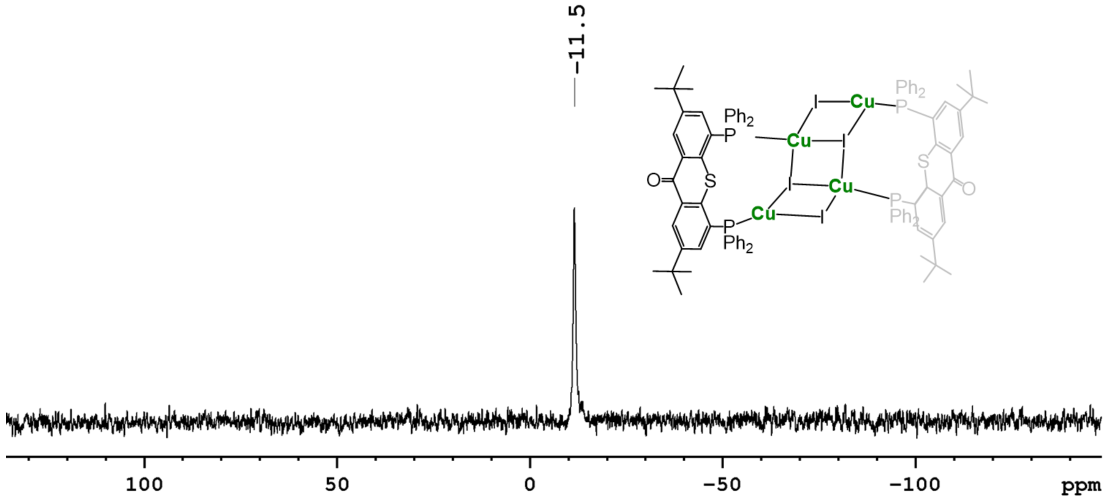

**Figure S8**. $^{31}P\{^{1}H\}$ NMR spectrum of **3** in $CDCl_3$ (162 MHz).

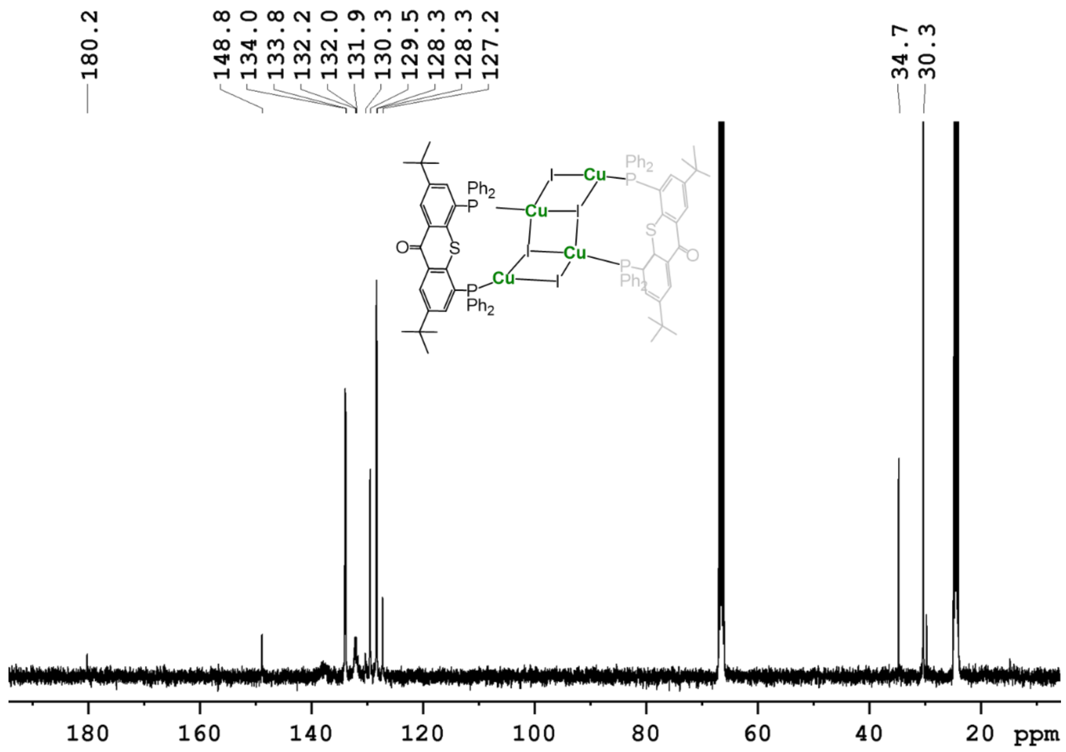


**Figure S9**. $^{13}C\{^1H\}$ NMR spectrum of **3** in in THF-$d_8$ (101 MHz).

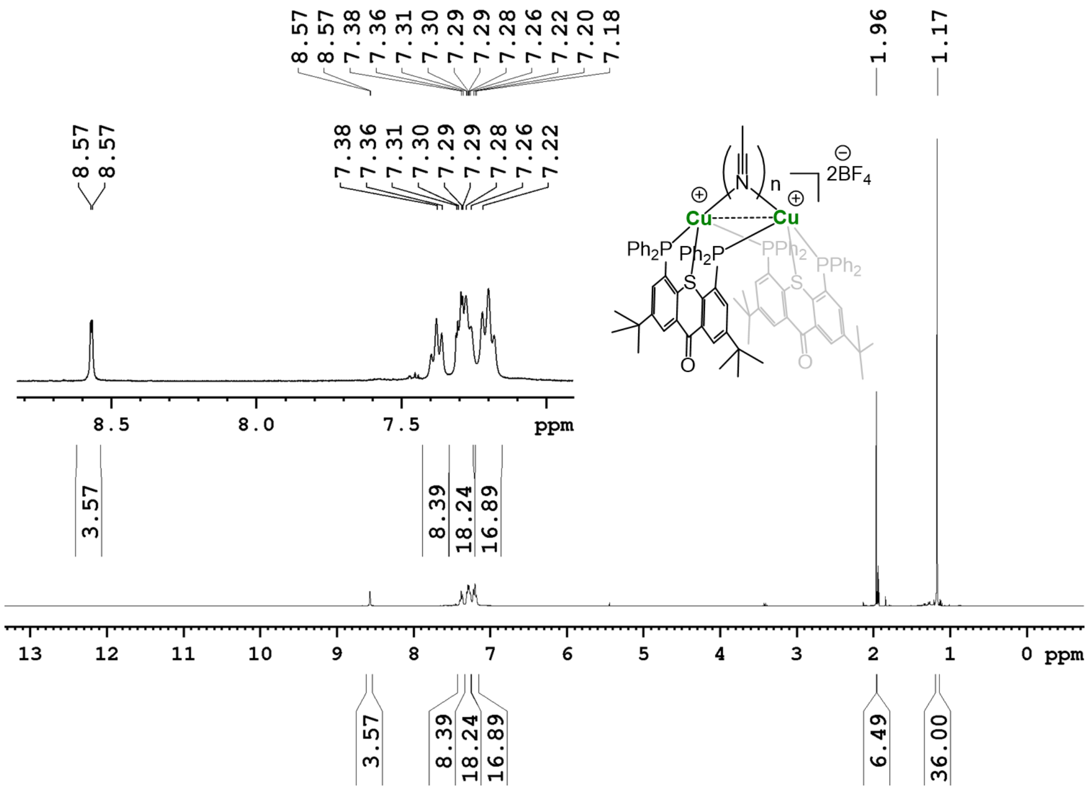


**Figure S10**. $^1H$ NMR spectrum of the **4a** in $CD_3CN$ (500 MHz).

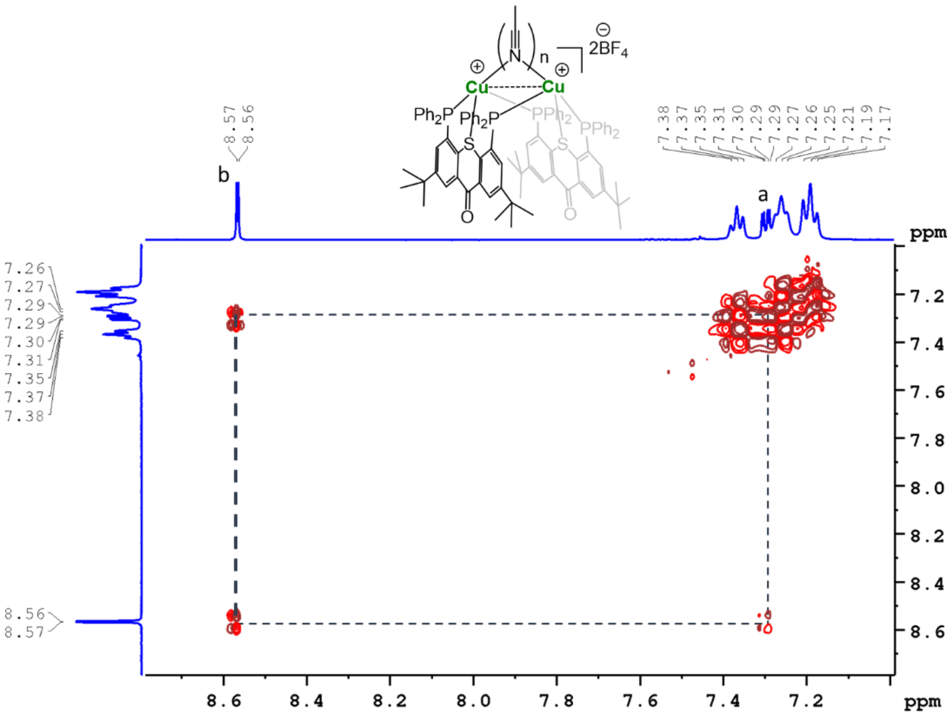


**Figure S11**. ${}^{1}H$–${}^{1}H$ COSY NMR (500 MHz) of **4a** in $CD_3CN$.

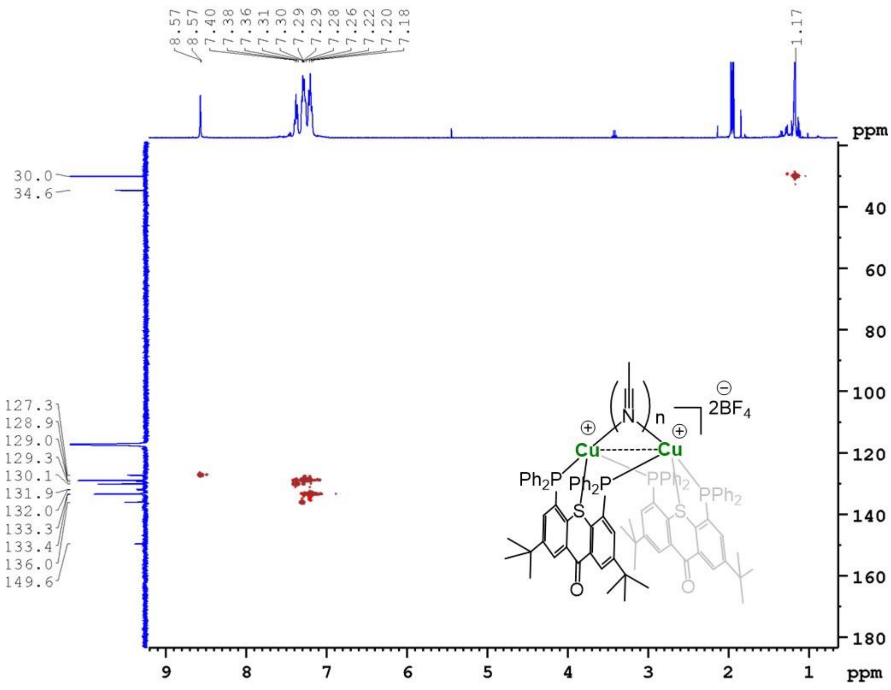


**Figure S12**. ${}^{1}H$–${}^{13}C$ HSQC NMR (500 MHz) of **4a** in $CD_3CN$.

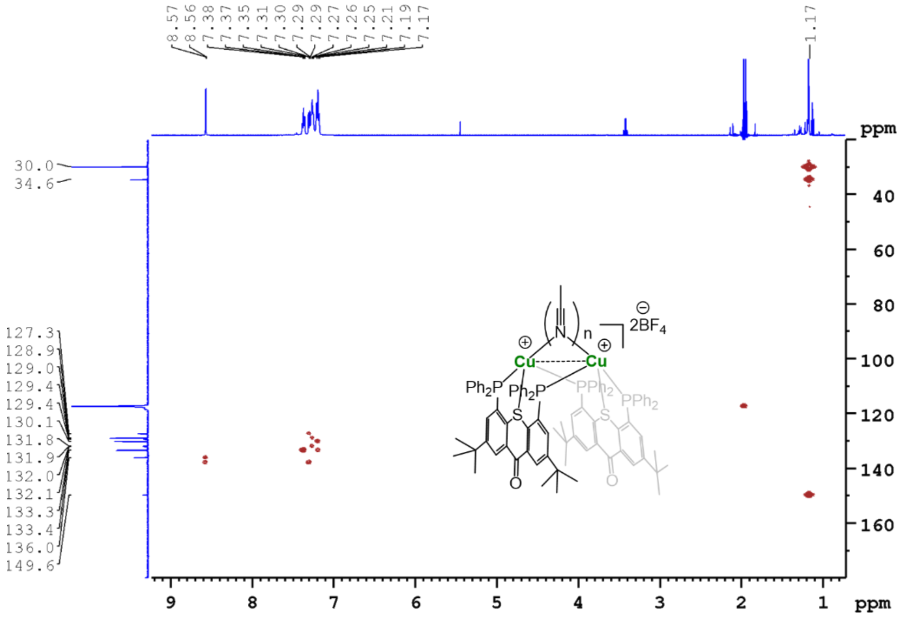


**Figure S13**. $^{1}H$–$^{13}C$ HMBC NMR (500 MHz) of **4a** in $CD_3CN$.

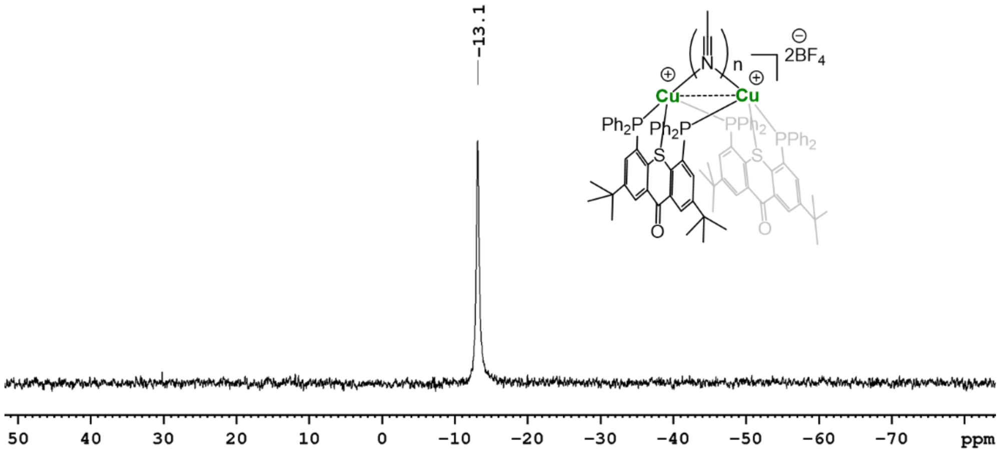


**Figure S14**. $^{31}P\{^{1}H\}$ NMR spectrum of **4a** in $CD_3CN$ (202 MHz).

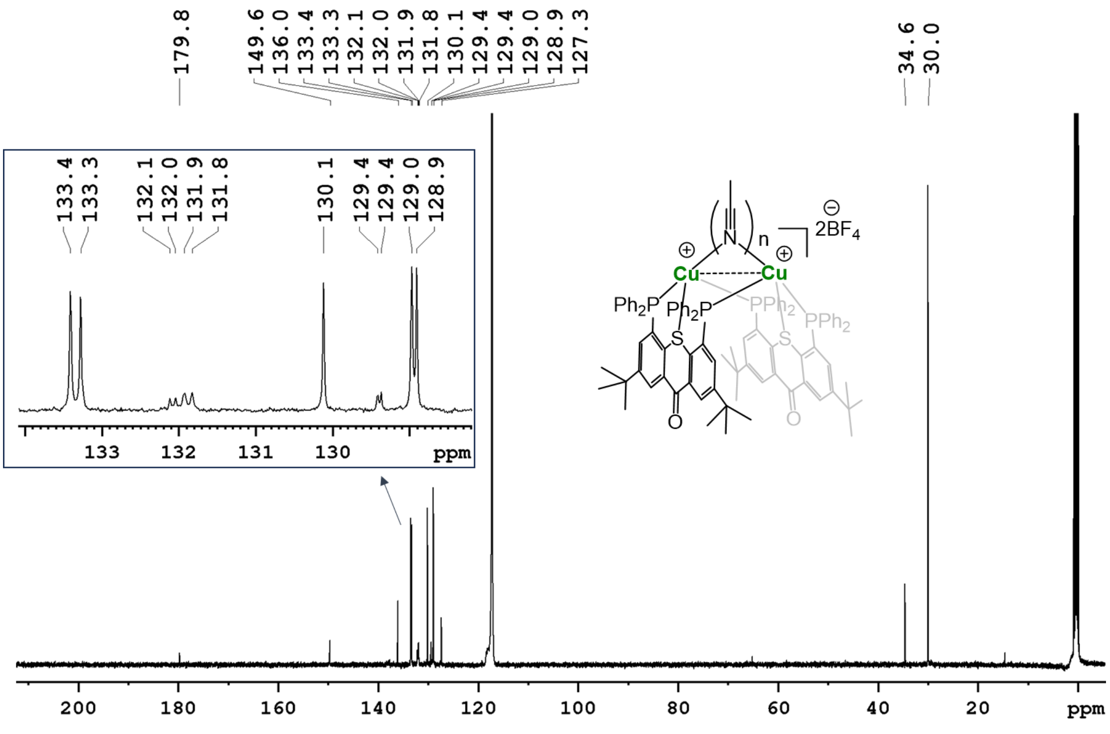


**Figure S15**. $^{13}C\{^1H\}$ NMR spectrum of **4a** in $CD_3CN$ (126 MHz).

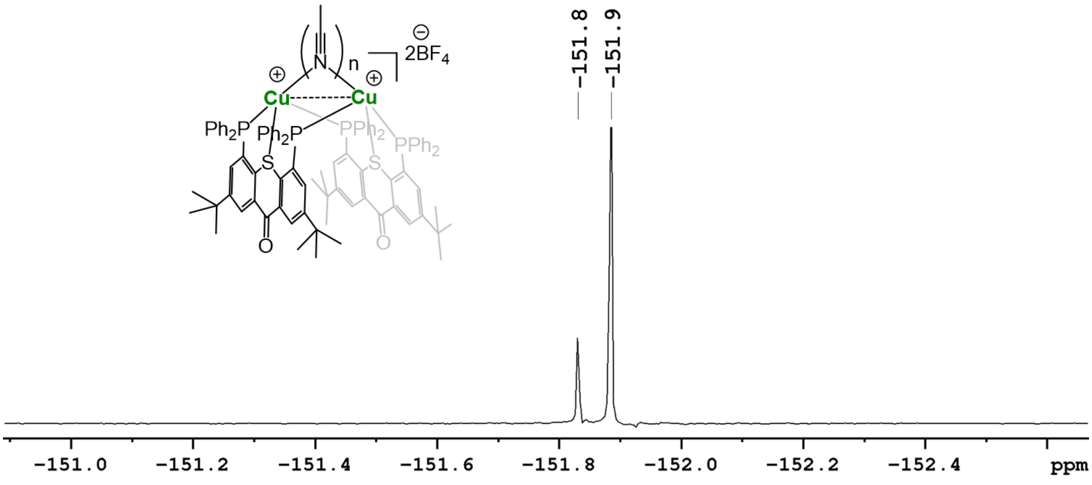


**Figure S16**. $^{19}F\{^1H\}$ NMR spectrum of **4a** in $CD_3CN$ (376 MHz).

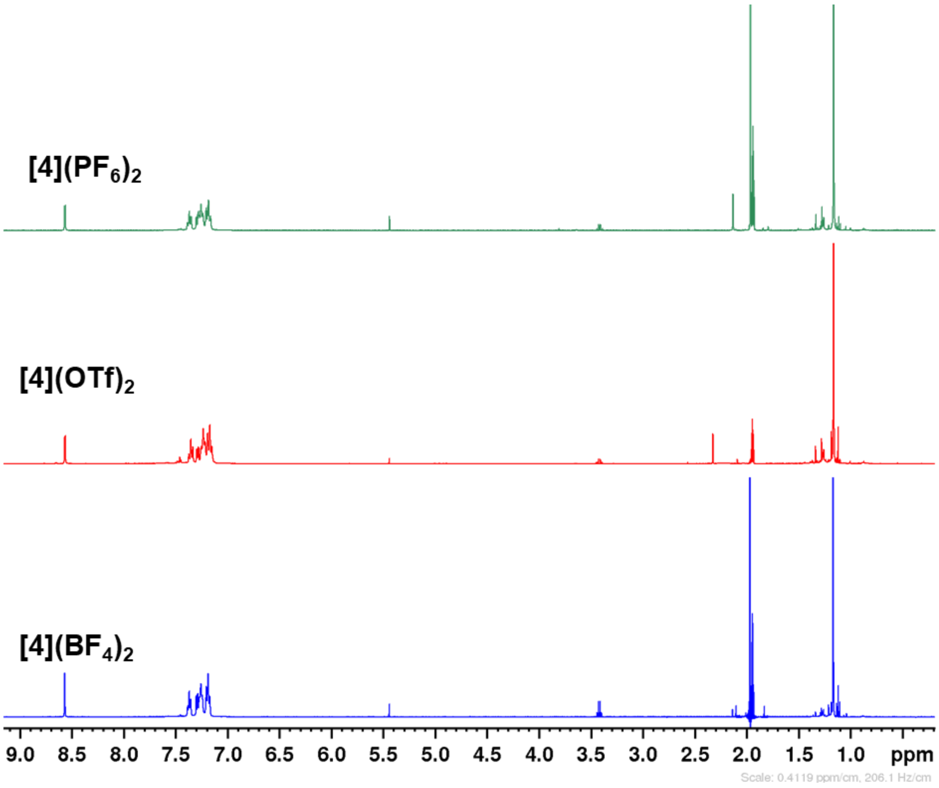


**Figure S17**. Stacked $^{1}$H NMR spectra of the **4a, 4b** and **4c** in $CD_3CN$.

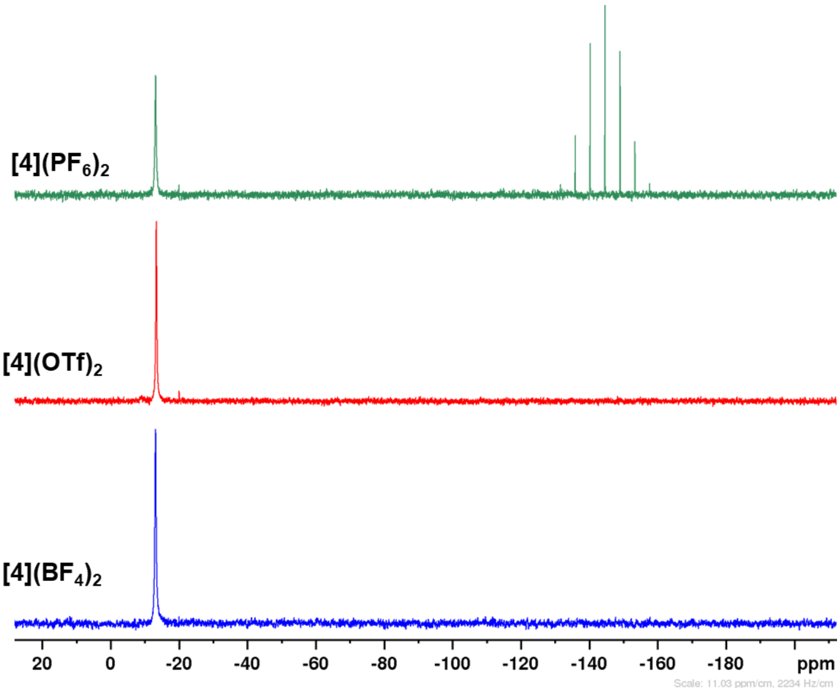


**Figure S18**. Stacked $^{31}$P NMR spectra of the **4a, 4b** and **4c** in $CD_3CN$.

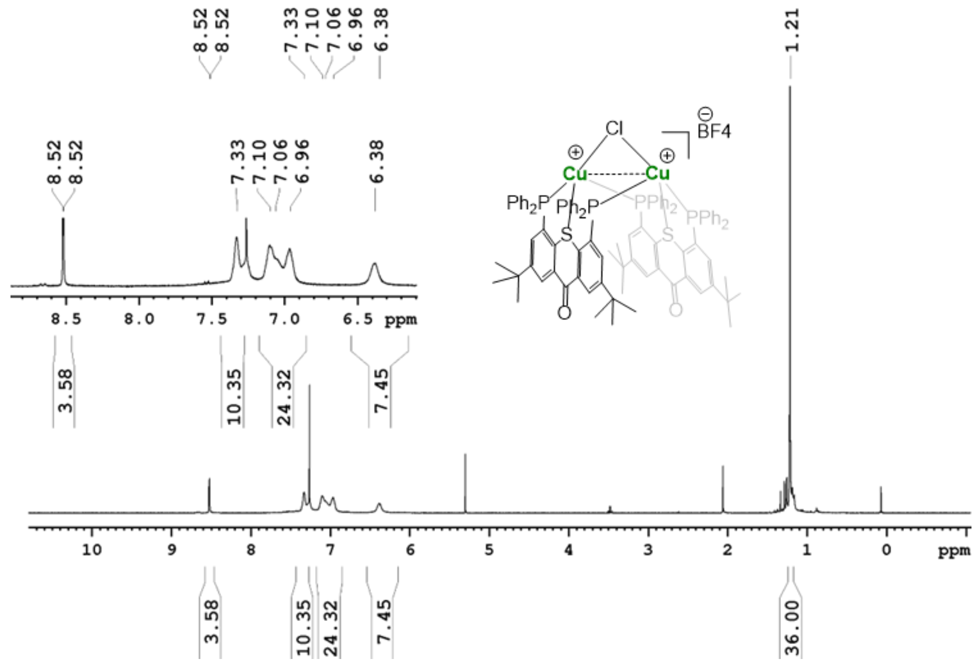


**Figure S19**. $^{1}H$ NMR spectrum of the **6** in $CDCl_3$ (400 MHz).

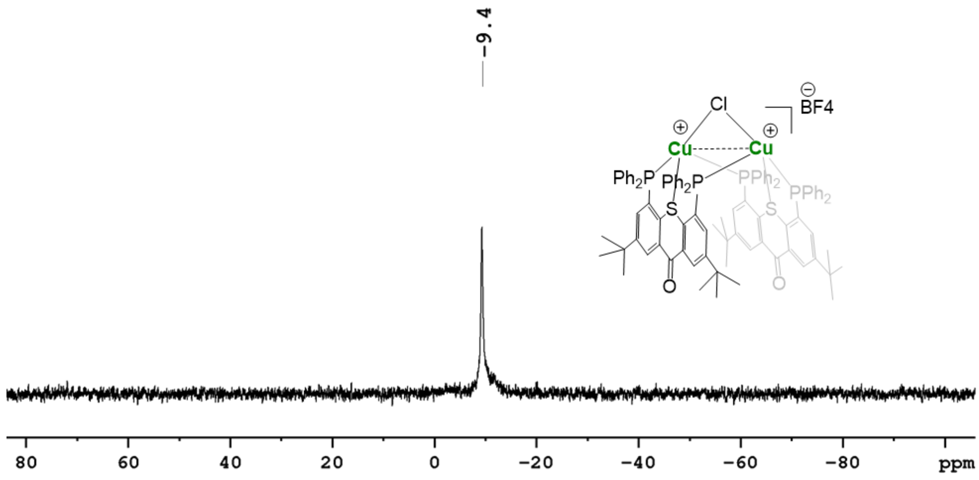


**Figure S20**. $^{31}P\{^{1}H\}$ NMR spectrum of **8** in $CDCl_3$ (162 MHz).

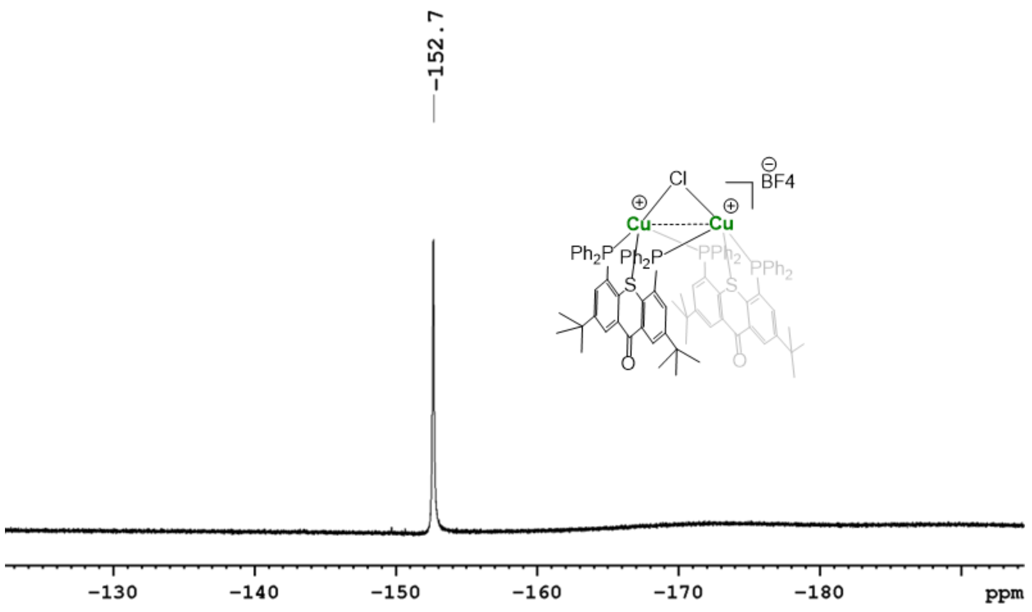


**Figure S21**. $^{19}F\{^{1}H\}$ NMR spectrum of **6** in $CD_3CN$ (376 MHz).

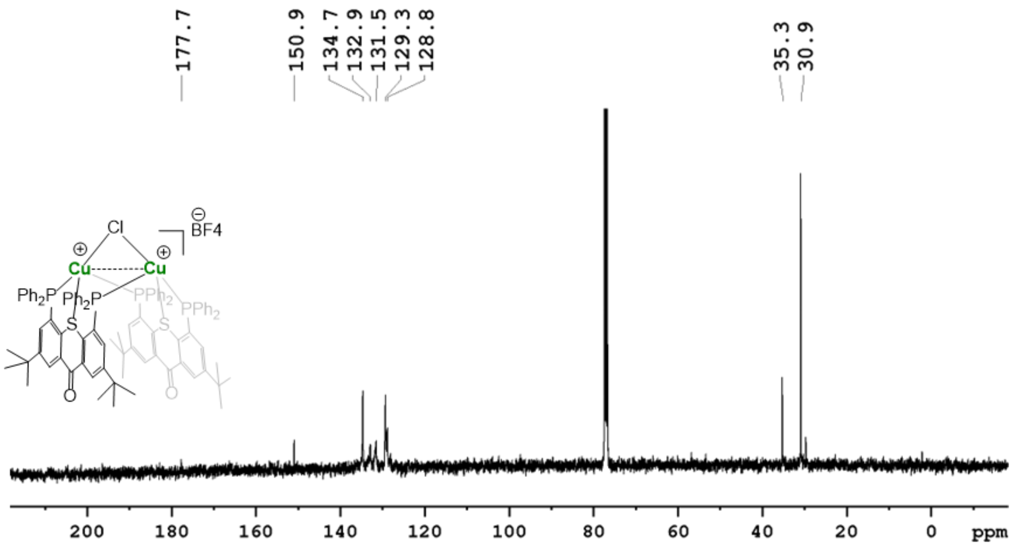


**Figure S22**. $^{13}C\{^{1}H\}$ NMR spectrum of **6** in $CDCl_3$ (126 MHz).

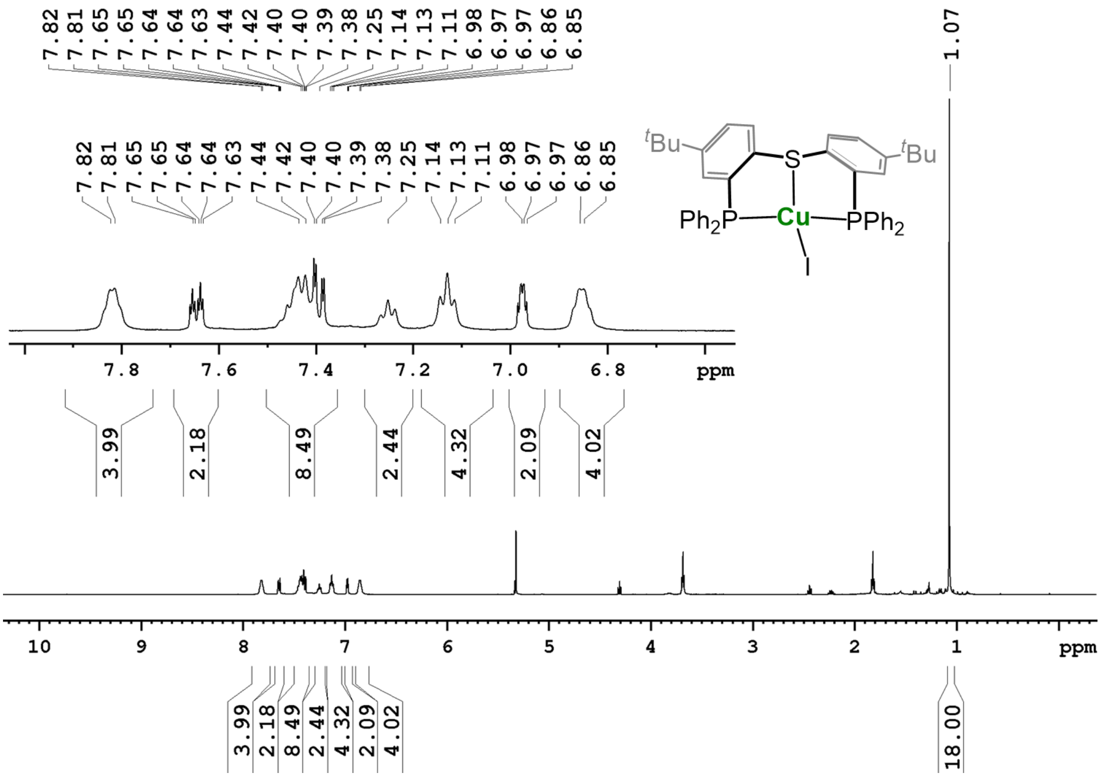

**Figure S23**. ¹H NMR spectrum of the **7** in $CD_2Cl_2$ (400 MHz).

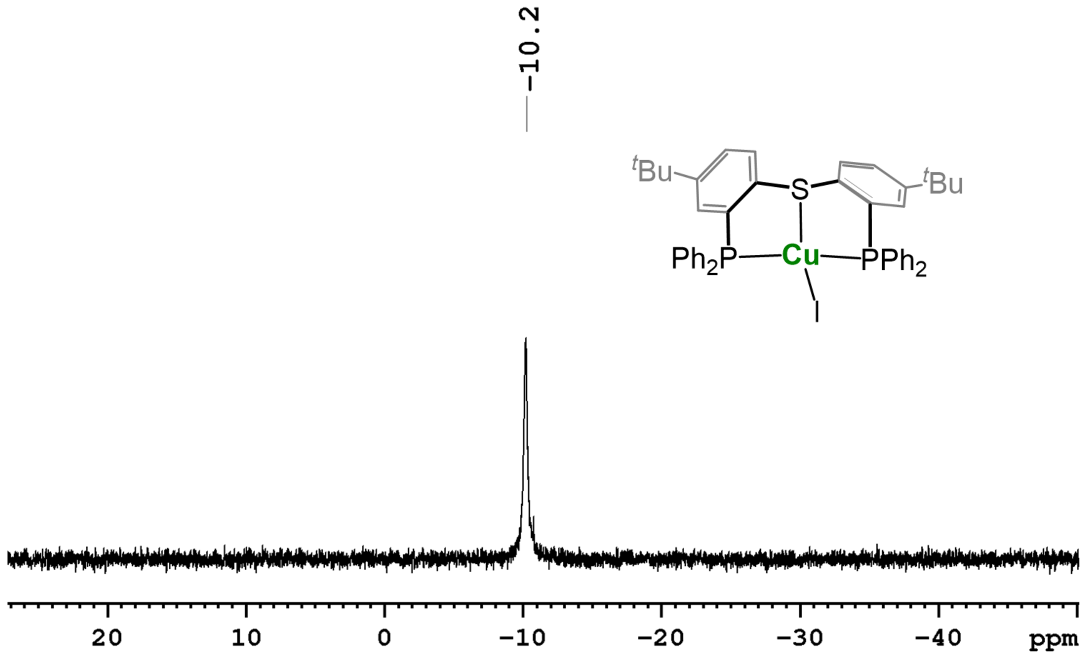

**Figure S24**. $^{31}P\{^1H\}$ NMR spectrum of **7** in $CD_2Cl_2$ (162 MHz).

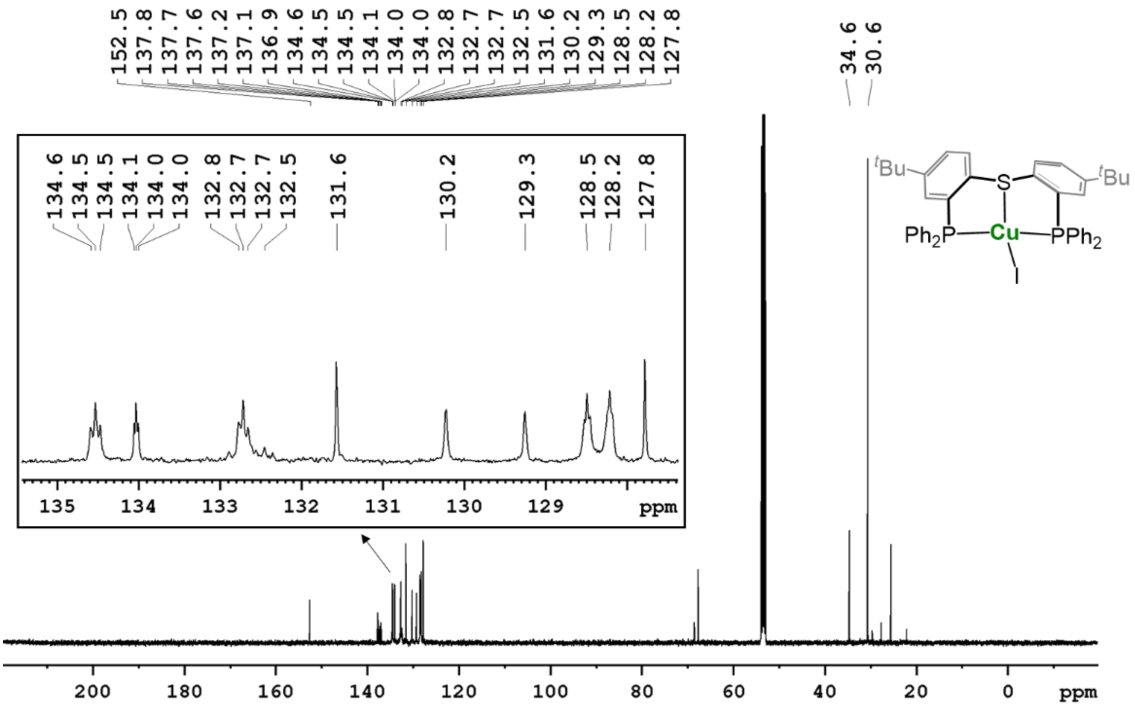


**Figure S25**. $^{13}C\{^{1}H\}$ NMR spectrum of **7** in $CD_2Cl_2$ (126 MHz).

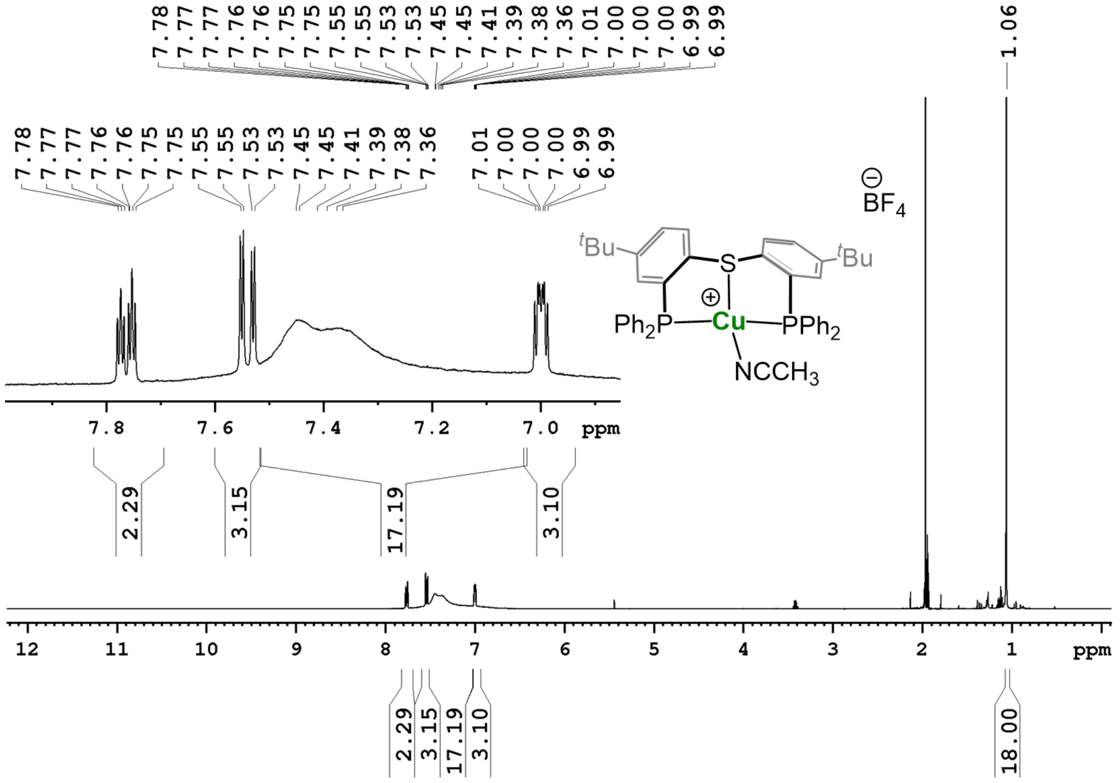


**Figure S26**. $^{1}H$ NMR spectrum of the **6** in $CD_3CN$ (400 MHz).

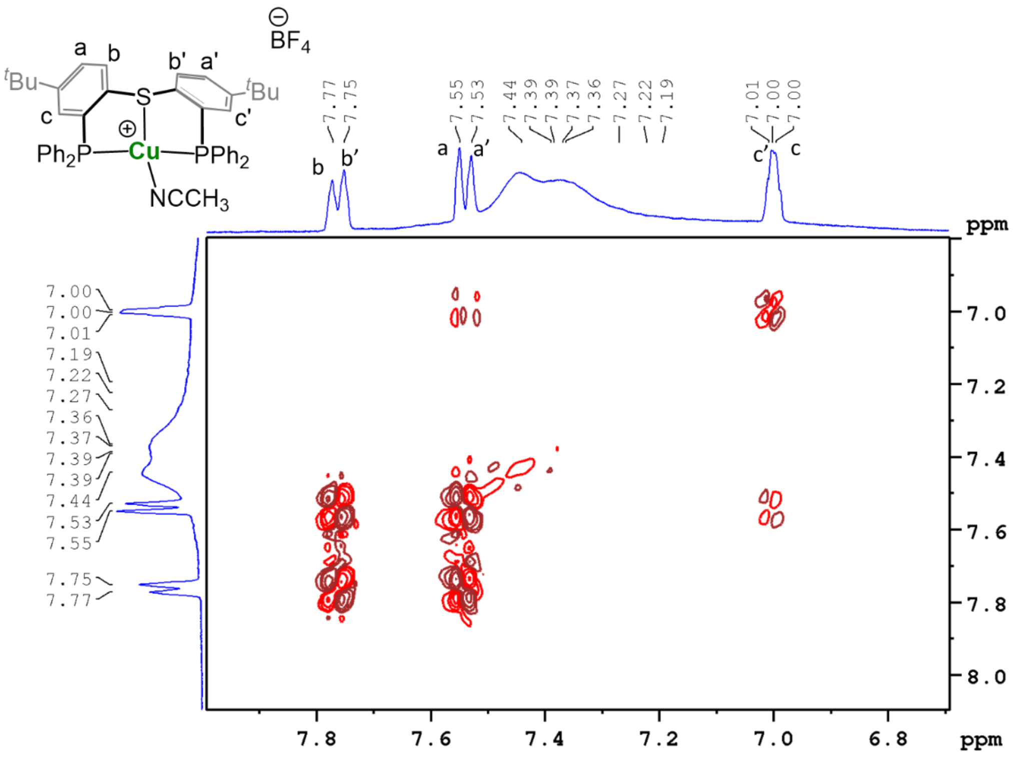

**Figure S27**. $^{1}$H–$^{1}$H COSY NMR (400 MHz) of **8** in $CD_3CN$.

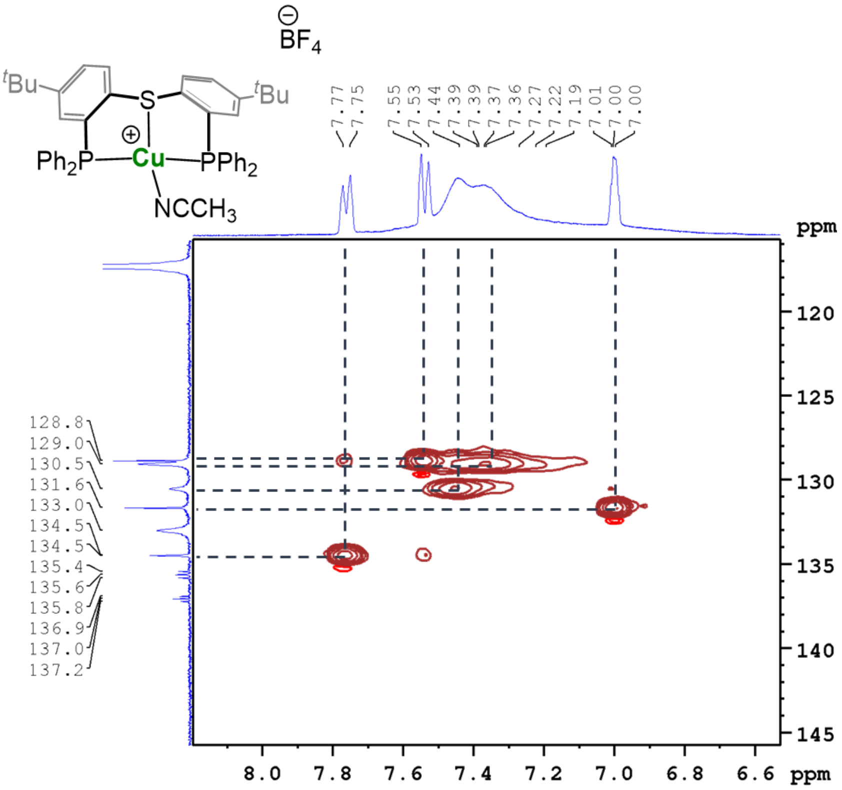

**Figure S28**. $^{1}$H–$^{13}$C HSQC NMR (500 MHz) of **8** in $CD_3CN$.

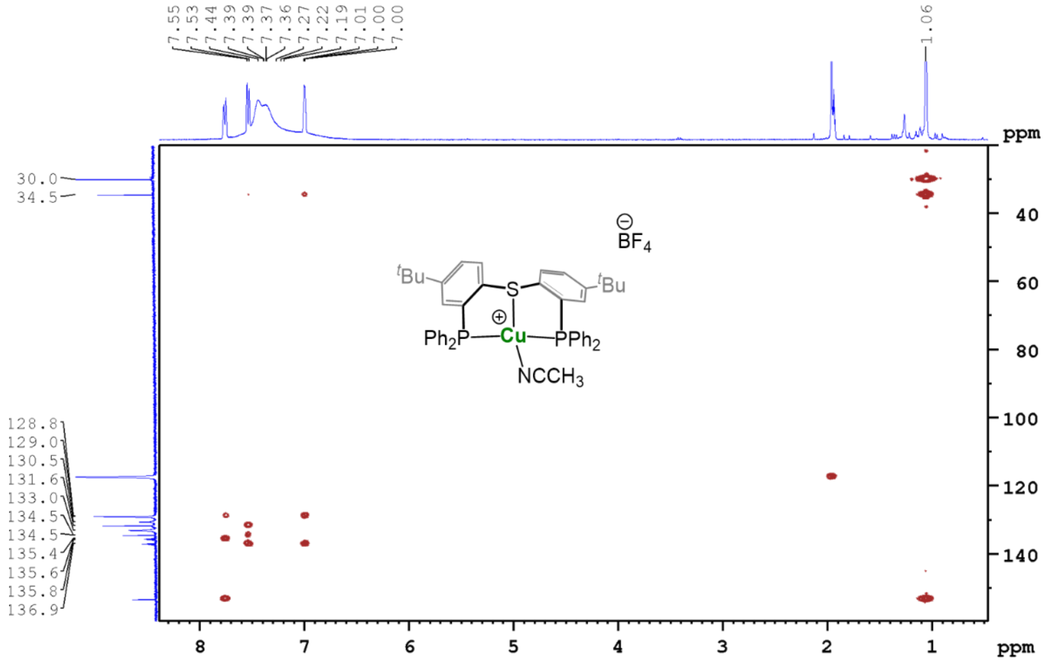

**Figure S29**. $^{1}H$–$^{13}C$ HMBC NMR (400 MHz) of **8** in $CD_3CN$.

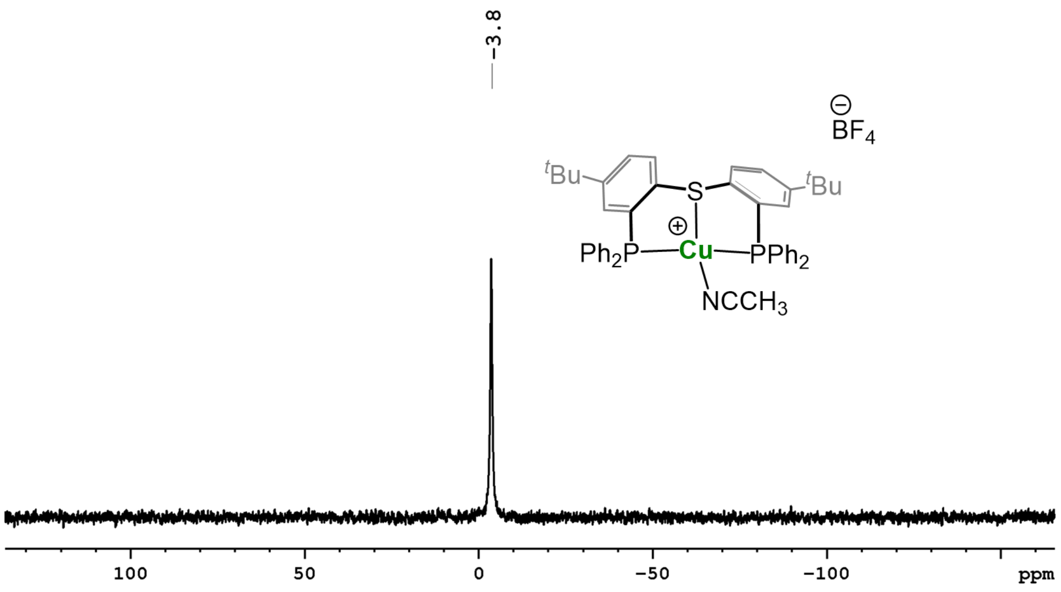

**Figure S30**. $^{31}P\{^{1}H\}$ NMR spectrum of **8** in $CD_3CN$ (162 MHz).

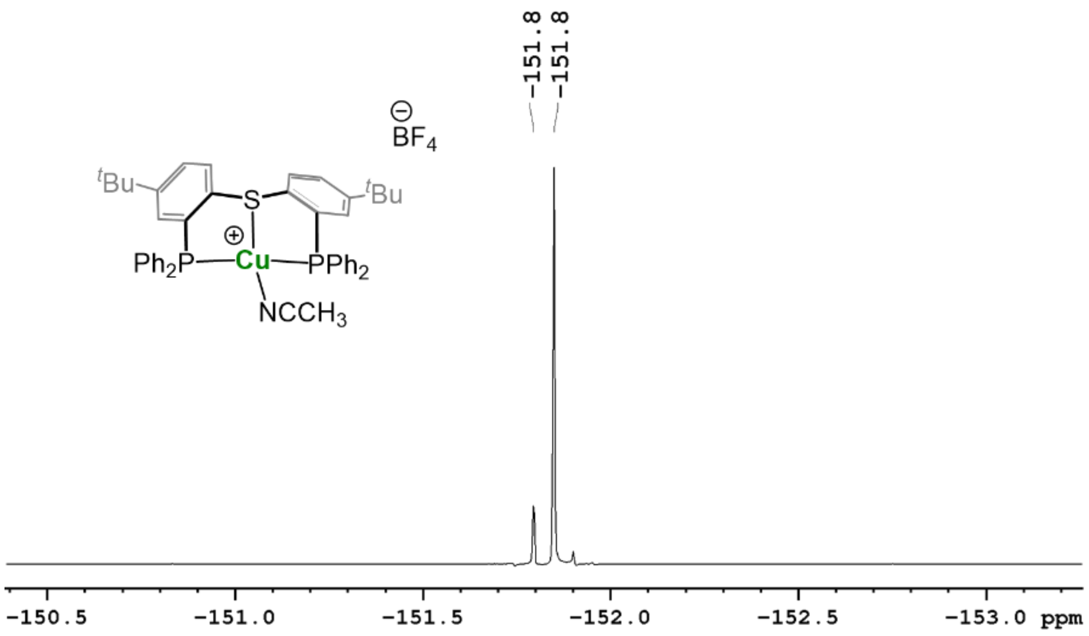


**Figure S31**. $^{19}F\{^1H\}$ NMR spectrum of **8** in $CD_3CN$ (376 MHz).

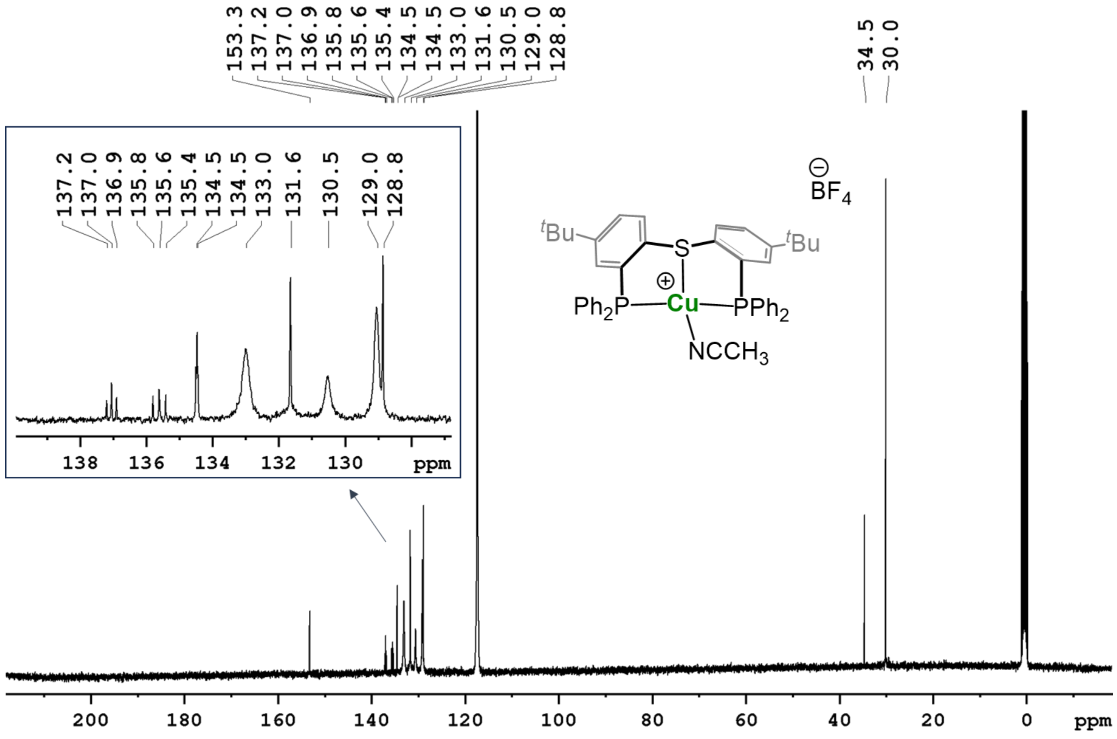


**Figure S32**. $^{13}C\{^1H\}$ NMR spectrum of in **8** $CD_3CN$ (126 MHz).

## 2. Thermogravimetric analysis (TGA)

The TGA measurement was performed in the range of 25-600° under a $N_2$ atmosphere with a heating range of 10°Cmin-1.

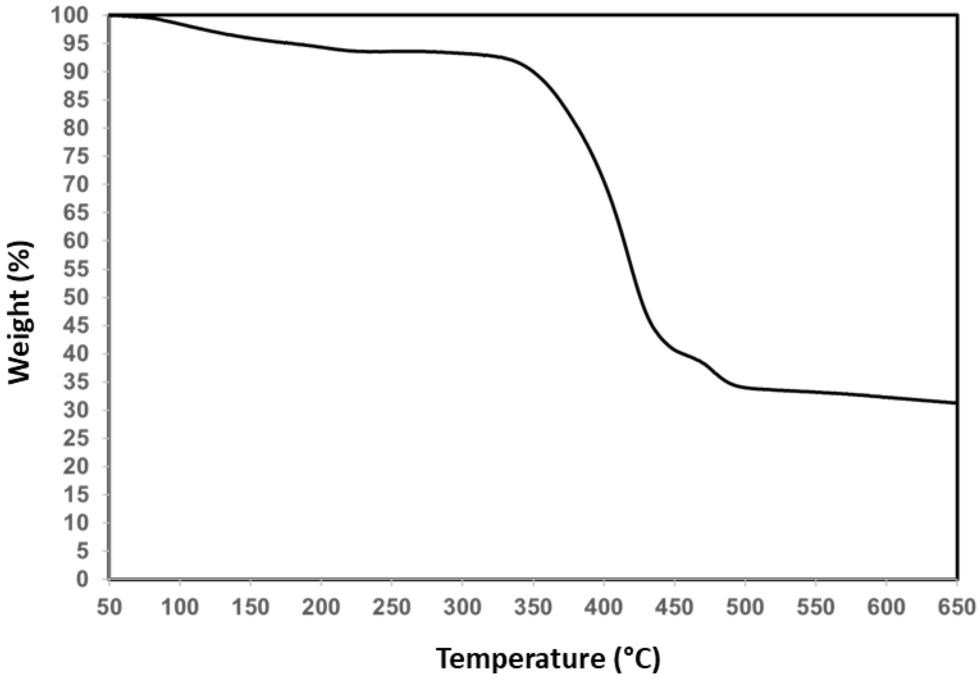


**Figure S33**. The thermogravimetric analysis (TGA) of complex **4a**.

## 3. Photophysical measurements

Photophysical measurements were performed with dilute solutions of the compounds in spectroscopic-grade THF, maintaining the absorbance of the lowest energy band at 0.05 to reduce self-absorption effects. All spectroscopic experiments utilized standard quartz cuvettes with a path length of 1 cm. The films were fabricated by drop-casting solutions of the emissive compounds in DCM. Solid-state experiments at low temperatures were conducted using an Optistat DN-X cryostat.

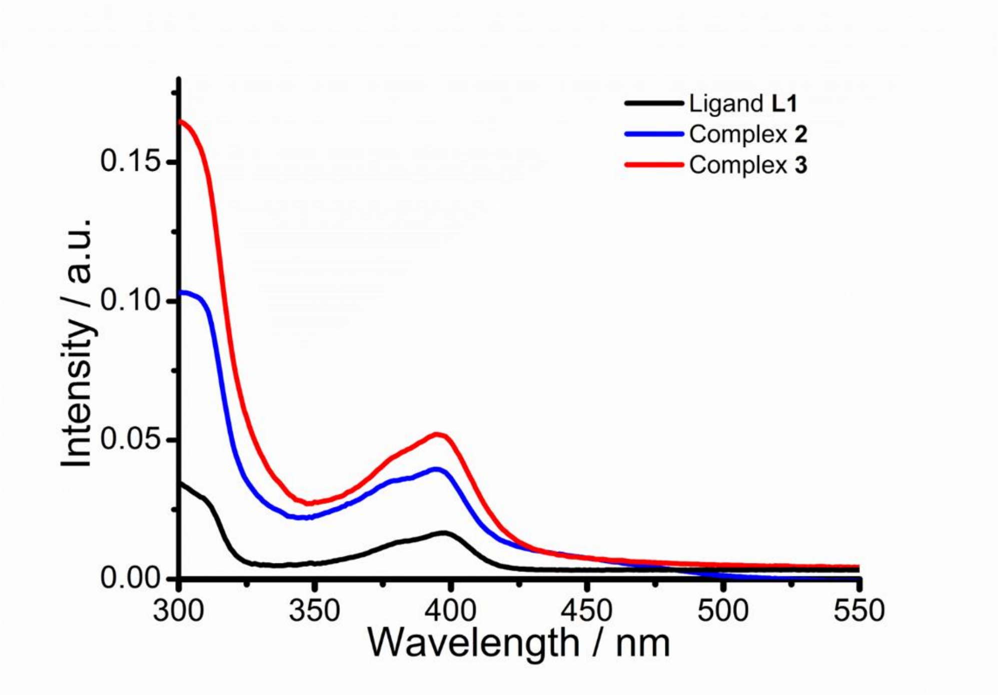


**Figure S34**. UV-vis. absorption spectra of ligand **L1** and complexes **2** and **3** in THF.

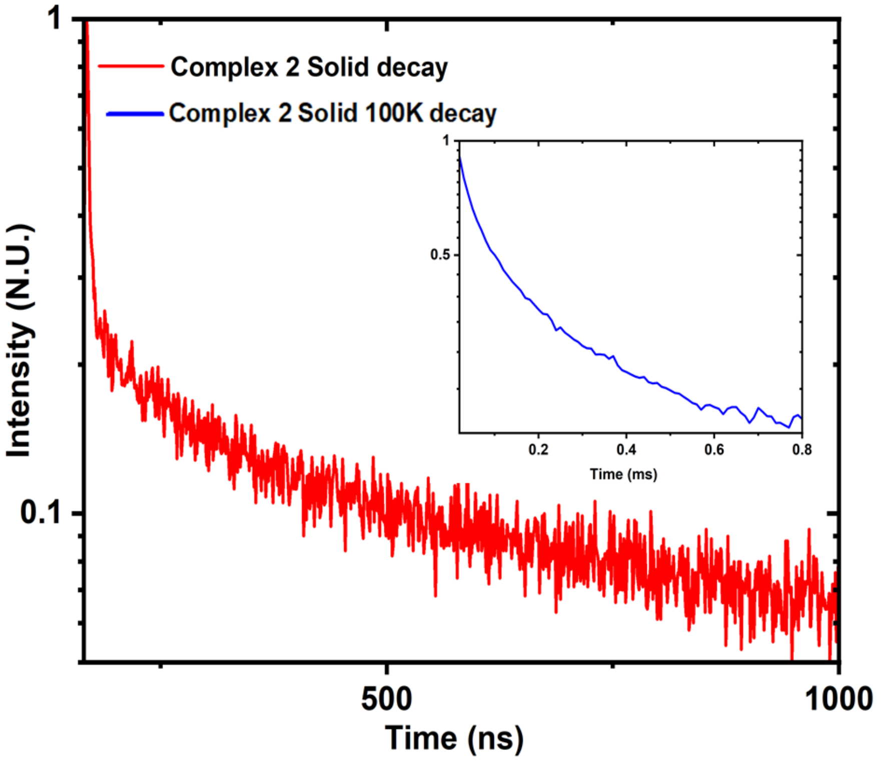


a.

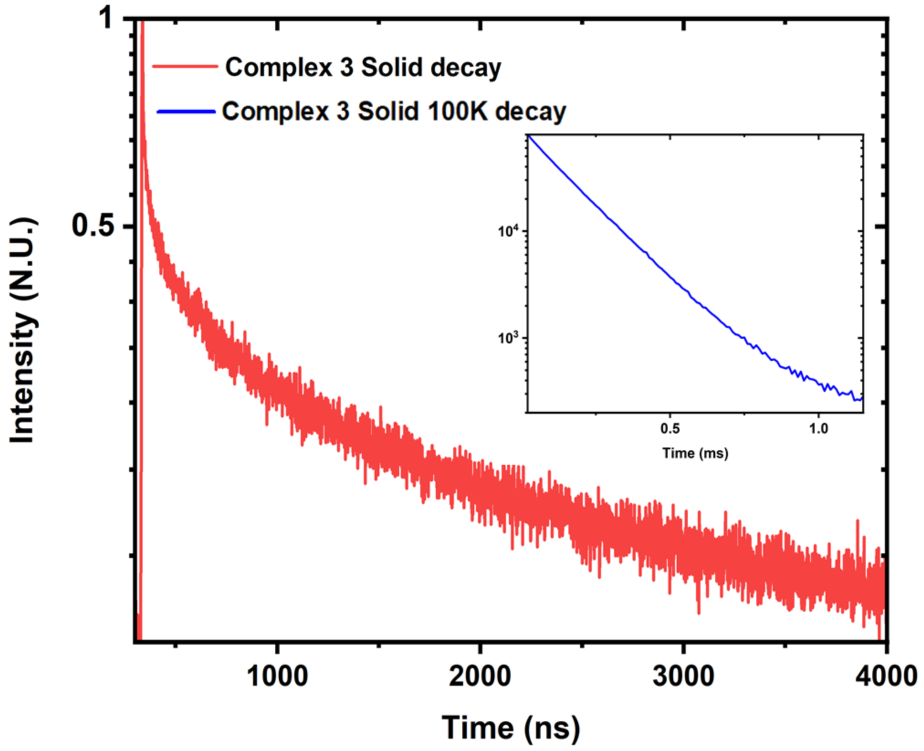


b.

**Figure S35**. Phosphorescence decay of complexes **2** (a) and **3** (b) at room temperature and at 100K (shown in insets).

## 4. Crystallography

### 4.1 General crystallographic and refinement details.

Suitable single crystals of **1**, **2**, **3, 5, 6, 7** and **8** were grown from the solvents mentioned in their respective synthetic procedures. Single-crystal X-ray diffraction data collections were performed using Rigaku goniometer diffractometer with graphite mono-chromated Mo Kα radiation (λ = 0.71073 Å). The diffraction intensity details were extracted from the diffraction frames using CrysAlisPro XtalLAB Synergy-S system (version 41.112a) program. The structures were then solved by SHELXT-9740 available in Olex2 crystallographic suite, which located most of the non-hydrogen atoms. Subsequently, least-squares refinements were carried out on $F^2$ using SHELXL-Version 2018/3 to locate the remaining non-hydrogen atoms. Non-hydrogen atoms were refined with anisotropic displacement parameters. Hydrogen atoms attached to carbon atoms were fixed in calculated positions. All disorders were handled with SADI, SIMU, EADP, SAME and RIGU constraints and restraints. The crystallographic parameters of each structure are given bellow. All structures are deposited to CCDC and their corresponding deposition numbers are listed below:

**CCDC: 1** = 2348189; **2** = 2348190; **3** = 2348191; **5** = 2377713; **6** = 2359930; **7** = 2377715; **8** = 2348192

**Table S1.** Crystallographic parameters of complexes **1-3.**

| | **1** | **2** | **3** |
|---|---|---|---|
| Empirical formula | $C_{97}H_{91}Cl_{24}Cu_3O_2P_4S_2$ | $C_{96}H_{96}Br_4Cu_4O_{3.5}P_4S_2$ | $C_{94}H_{88}Cl_{12}Cu_4I_4O_2P_4S_2$ |
| Formula weight | 2518.11 | 2067.52 | 2624.80 |
| Wavelength (Å) | 0.71073 | 0.71073 | 0.71073 |
| Temperature (K) | 200.0(4) | 200.0(4) | 292.9(4) |
| Crystal system | monoclinic | monoclinic | monoclinic |
| Space group | *P12/c1* | *C12/c1* | *P121/c1* |
| a/Å | 20.6462(5) | 34.5022(13) | 12.0656(6) |
| b/Å | 13.7249(2) | 10.9590(3) | 22.7227(5) |
| c/Å | 22.1899(5) | 25.9833(8) | 24.7219(13) |
| α/degree | 90 | 90.00 | 90 |
| β/degree | 116.804(3) | 109.759(4) | 130.922(8) |
| γ/degree | 90 | 90.00 | 90 |
| Volume (Å$^3$) | 5612.3(2) | 9246.1(6) | 5121.3(6) |
| Z | 2 | 4 | 2 |
| $D_{calcd}$, g cm$^{-3}$ | 1.490 | 1.485 | 1.702 |
| μ/mm$^{-1}$ | 1.274 | 2.799 | 2.487 |
| F(000) | 2552 | 4192 | 2584 |
| 2θ range (degree) | 2.21 to 50 | 2.21 to 50 | 2.18 to 50 |
| Total/ unique no. of reflns | 63247 / 8960 | 50470 / 8139 | 42342 / 9009 |
| $R_{int}$ | 0.0341 | 0.0575 | 0.0296 |
| GOF (F$^2$) | 1.033 | 1.036 | 1.063 |
| $R_1$, $wR_2$ | 0.0471, 0.1252 | 0.0571, 0.1665 | 0.0339, 0.1077 |

**Table S2.** Crystallographic parameters of complexes **5-8.**

| | **5** | **6** | **7** | **8** |
|---|---|---|---|---|
| Empirical formula | $C_{94}H_{97}B_2Cu_4F_8O_4P_4S_2$ | $C_{123}H_{133}BCl_{12}Cu_2F_4P_4S_2$ | $C_{44}H_{44}CuIP_2S$ | $C_{52}H_{59}BCuNO_{1.5}P_2S$ |
| Formula weight | 1807.80 | 2163.11 | 857.23 | 966.35 |
| Wavelength (Å) | 0.71073 | 0.71073 | 0.71073 | 0.71073 |
| Temperature (K) | 149.99(10) | 219.99(13) | 149.99(10) | 150.00(10) |
| Crystal system | monoclinic | trigonal | monoclinic | triclinic |
| Space group | *P12/c1* | *P3121* | *C12/c1* | *P-1* |
| a/Å | 23.4782(14) | 25.2661(5) | 23.0022(4) | 14.2208(3) |
| b/Å | 17.0073(9) | 25.2661(5) | 14.4528(2) | 14.2834(3) |
| c/Å | 26.5918(13) | 16.2303(3) | 26.2983(5) | 14.5245(3) |
| α/degree | 90 | 90 | 90.00 | 77.027(2) |
| β/degree | 108.726(6) | 90 | 112.265(2) | 62.975(2) |
| γ/degree | 90 | 120 | 90.00 | 71.182(2) |
| Volume (Å$^3$) | 10056.0(10) | 8972.9(4) | 8090.9(3) | 2476.97(10) |
| Z | 4 | 3 | 8 | 2 |
| $D_{calcd}$, g cm$^{-3}$ | 1.194 | 1.201 | 1.407 | 1.296 |
| μ/mm$^{-1}$ | 0.588 | 0.540 | 1.463 | 0.601 |
| F(000) | 3757 | 3407 | 3488 | 1012 |
| 2θ range (degree) | 2.09 to 50 | 2.0 to 50 | 2.11 to 50.48 | 2.08 to 49.99 |
| Total/ unique no. of reflns | 45474 / 9532 | 35536 / 8277 | 70317 / 10671 | 35455 / 7764 |
| $R_{int}$ | 0.0769 | 0.0432 | 0.0278 | 0.0263 |
| GOF ($F^2$) | 1.125 | 1.046 | 1.037 | 1.086 |
| $R_1$, $wR_2$ | 0.0939, 0.2651 | 0.0583, 0.1594 | 0.0237, 0.0572 | 0.0361, 0.1043 |

## 5. Computational data

**Table S3.** NBOs participating in the Cu-L bonding (L = MeCN or $Me_2CO$)

| NBO # | Atom | Orbital type* | Hybridization | Occupancy |
|---|---|---|---|---|
| Complex **A** (L = MeCN) | | | | |
| 102 | N57 | LP | *s* (49.98%)<br>*p* (49.97%) | 1.84535 |
| 182 | Cu1 | LV | *s* (99.29%) | 0.45134 |
| 183 | Cu23 | LV | *s* (99.24%) | 0.44958 |
| Complex **D** (L = $Me_2CO$) | | | | |
| 90 | O5 | LP | *s* (60.77%)<br>*p* (39.19%) | 1.91699 |
| 91 | O5 | LP | *p* (99.95%) | 1.89486 |
| 187 | Cu1 | LV | *s* (99.18%) | 0.43691 |
| 188 | Cu33 | LV | *s* (99.18%) | 0.43692 |

*LP – lone pair, LV – lone vacant.

**Table S4.** Second order perturbation energies of the Cu-L orbital interactions (L = MeCN or $Me_2CO$).

| **From NBO to NBO** (LP→LV)* | $E^2$ (kcal/mol) |
|---|---|
| Molecule **A** (L = MeCN) | |
| 102 → 182 | 23.81 |
| 102 → 183 | 25.94 |
| Molecule **D** (L = $Me_2CO$) | |
| 90 → 187 | 14.56 |
| 90 → 188 | 14.56 |
| 91 → 187 | 6.81 |
| 91 → 188 | 6.81 |

*Orbital numbers are the same as in **Table S3**.

**Table S5.** QTAIM analysis results - XYZ coordinates of selected atoms and bond critical points (BCPs) along with electron densities and electron densities Laplacian values at those BCPs.

| Atom/BCP | X | Y | Z | Electron density ($\rho$) | Electron density Laplacian ($\nabla^2\rho$) |
|---|---|---|---|---|---|
| Complex **A** (L = MeCN) | | | | | |
| N57 | 0.02561 | 6.01763 | -0.01584 | - | - |
| Cu1 | -2.32582 | 3.10726 | 1.14191 | - | - |
| Cu23 | 2.30162 | 3.11696 | -1.22939 | - | - |
| N57-Cu1 | -1.16018 | 4.53719 | 0.56962 | 0.06857 | 0.29223 |
| N57-Cu23 | 1.17766 | 4.54374 | -0.62986 | 0.07159 | 0.30851 |
| Complex **D** (L = $Me_2CO$) | | | | | |
| O5 | -0.00003 | 5.86473 | -0.00005 | - | - |
| Cu1 | 2.77161 | 3.35842 | -1.35904 | - | - |
| Cu33 | -2.77165 | 3.35841 | 1.35909 | - | - |

| | | | | | |
|---|---|---|---|---|---|
| O5-Cu1 | 1.42931 | 4.62218 | -0.71552 | 0.05886 | 0.29511 |
| O5-Cu33 | -1.42936 | 4.62217 | 0.71553 | 0.05886 | 0.29510 |

**Table S6.** Selected bond lengths of the optimized geometries of **L1*** in the $S_0$ and $T_1$ states.

| **Bond** | **Bondlength (Å)** | |
|---|---|---|
| | Ground state ($S_0$) | Excited triplet state ($T_1$) |
| C1-P1 | 1.863 | 1.900 |
| C4-P2 | 1.863 | 1.900 |
| S1-C2 | 1.772 | 1.813 |
| S1-C3 | 1.772 | 1.813 |
| O1-C4 | 1.232 | 1.305 |
| C2-C5 | 1.408 | 1.432 |
| C3-C6 | 1.408 | 1.432 |

**Table S7**. Selected bond lengths of the optimized geometries of **3*** in the $S_0$ and $T_1$ states.

| **Bond** | **Bondlength (Å)** | |
|---|---|---|
| | Ground state ($S_0$) | Excited triplet state ($T_1$) |
| Cu4-I1 | 2.541 | 2.693 |
| Cu4-I2 | 2.549 | 2.663 |
| Cu72-I1 | 2.750 | 2.889 |
| Cu72-I2 | 2.701 | 2.800 |
| Cu72-I70 | 2.674 | 2.789 |
| Cu3-I70 | 2.750 | 2.859 |
| Cu3-I1 | 2.674 | 2.752 |
| Cu3-I71 | 2.701 | 2.845 |
| Cu73-I70 | 2.541 | 2.678 |
| Cu73-I71 | 2.549 | 2.653 |
| Cu3-P5 | 2.261 | 2.407 |
| Cu4-P7 | 2.231 | 2.358 |
| Cu72-P74 | 2.261 | 2.400 |
| Cu72-P74 | 2.231 | 2.400 |
| Cu4-Cu72 | 2.810 | 3.211 |
| Cu3-Cu72 | 3.147 | 3.466 |
| Cu3-Cu73 | 2.810 | 3.328 |

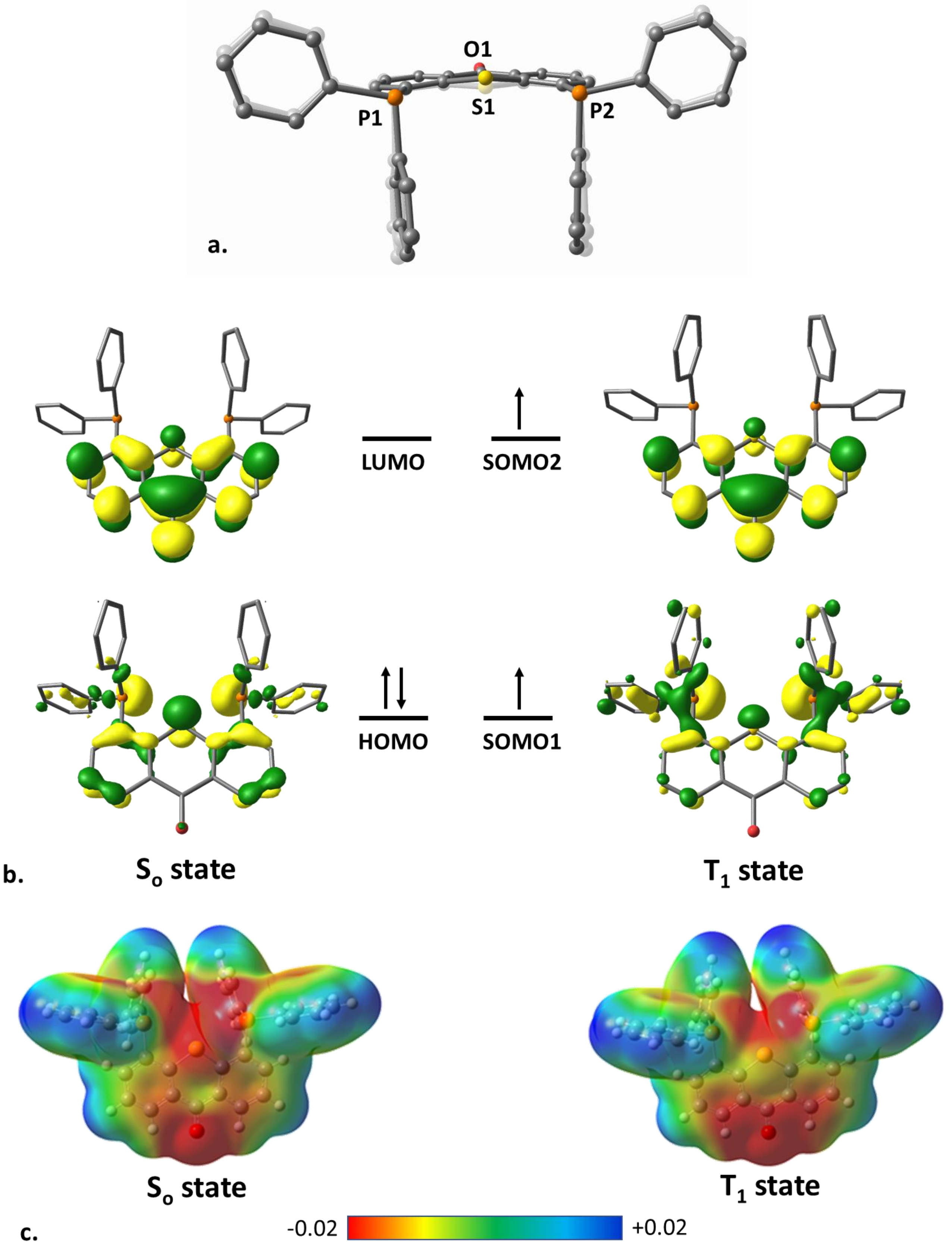


**Figure S36.** Overlaid optimized geometries of the model ligand **L1*** in the $S_0$ and $T_1$ states ($T_1$ geometry is shown at the forefront) (a) their corresponding frontier MOs (b) and electrostatic surface potential (ESP) plots (c). Hydrogen atoms and phenyl rings are omitted for clarity**.**

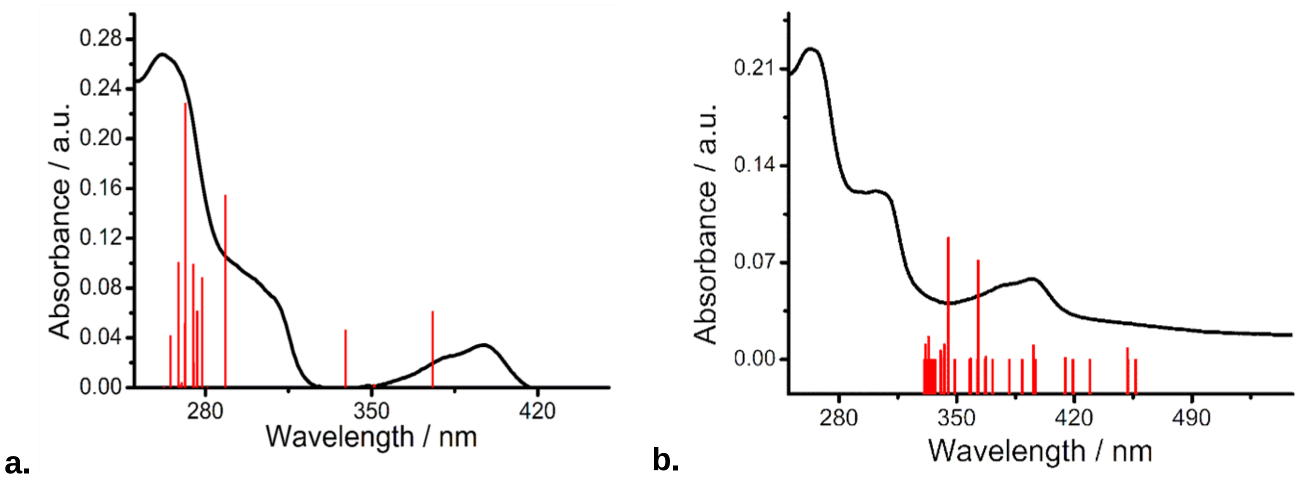


**Figure S37.** Overlay of the experimental absorption spectra of (black trace) with oscillator strength values of the calculated transitions (red bars) for ligand **L1** (**L1***) (a) and complex **3** (**3***) (b).

**Table S8:** Experimental and calculated electronic transitions of ligand **L1** and **L1***, respectively.

| **Experimental** | **Calculated (TD-DFT, (U)B3LYP/CPCM/THF))** | | |
|---|---|---|---|
| $\lambda_{max}$ (nm) | λ (nm) ($f_{osc}$) | Transition | Character |
| 397 | 375.72 (0.0613) | HOMO(α) -> LUMO(α)(49%) | PSP(lp) -> π* TX |
| | | HOMO(β) -> LUMO(β)(49%) | PSP(lp) -> π* TX |
| 381 | 339.07 (0.0464) | HOMO-2(α) -> LUMO(α)(48%) | PSP(lp) -> π* TX |
| | | HOMO-2(β) -> LUMO(β)(48%) | PSP(lp) -> π* TX |
| 308 | 288 (0.1546) | HOMO(α) -> LUMO+2(α)(43%) | PSP(lp) -> π* TX+ π* Phenyl |
| | | HOMO(β) -> LUMO+2(β)(43%) | PSP(lp) -> π* TX+ π* Phenyl |
| 271 | 274.93 (0.0992) | HOMO-6(α) -> LUMO(α)(26%) | π* Phenyl-> π* TX |
| | | HOMO(α) > LUMO+3(α)(10%) | PSP(lp) -> π* TX |
| | | HOMO-6(β) -> LUMO(β)(26%) | π* Phenyl-> π* TX |
| | | HOMO(β) > LUMO+3(β)(10%) | PSP(lp) -> π* TX |

**Table S9:** Experimental and calculated electronic transitions of complexes **3** and **3***, respectively.

| **Experimental** | **Calculated (TD-DFT, (U)B3LYP/CPCM/THF))** | | |
|---|---|---|---|
| $\lambda_{max}$ (nm) | λ (nm) ($f_{osc}$) | Transition | Character |
| 452 | 451.61 (0.0084) | HOMO(α) -> LUMO+1(α)(48%) | Cu/I→ π* TX |
| | | HOMO(β) -> LUMO+1(β)(48%) | Cu/I→ π* TX |
| 395 | 395.78 (0.0101) | HOMO-2(α) -> LUMO+1(α)(48%) | Cu/I→ π* TX |
| | | HOMO-2(β) -> LUMO+1(β)(48%) | Cu/I→ π* TX |
| 372 | 362.73 (0.0716) | HOMO-5(α) → LUMO(α) (27%) | Cu/I→ π* TX |
| | | HOMO-4(α) → LUMO+1(α) (6%) | Cu/I→ π* TX |
| | | HOMO-3(α) → LUMO(α) (12%) | Cu/I→π* π* TX |
| | | HOMO-5(β) → LUMO(β) (27%) | Cu/I→ π* TX |
| | | HOMO-4(β) → LUMO+1(β) (6%) | Cu/I→ π* TX |
| | | HOMO-3(β) → LUMO(β) (12%) | Cu/I→π* TX |

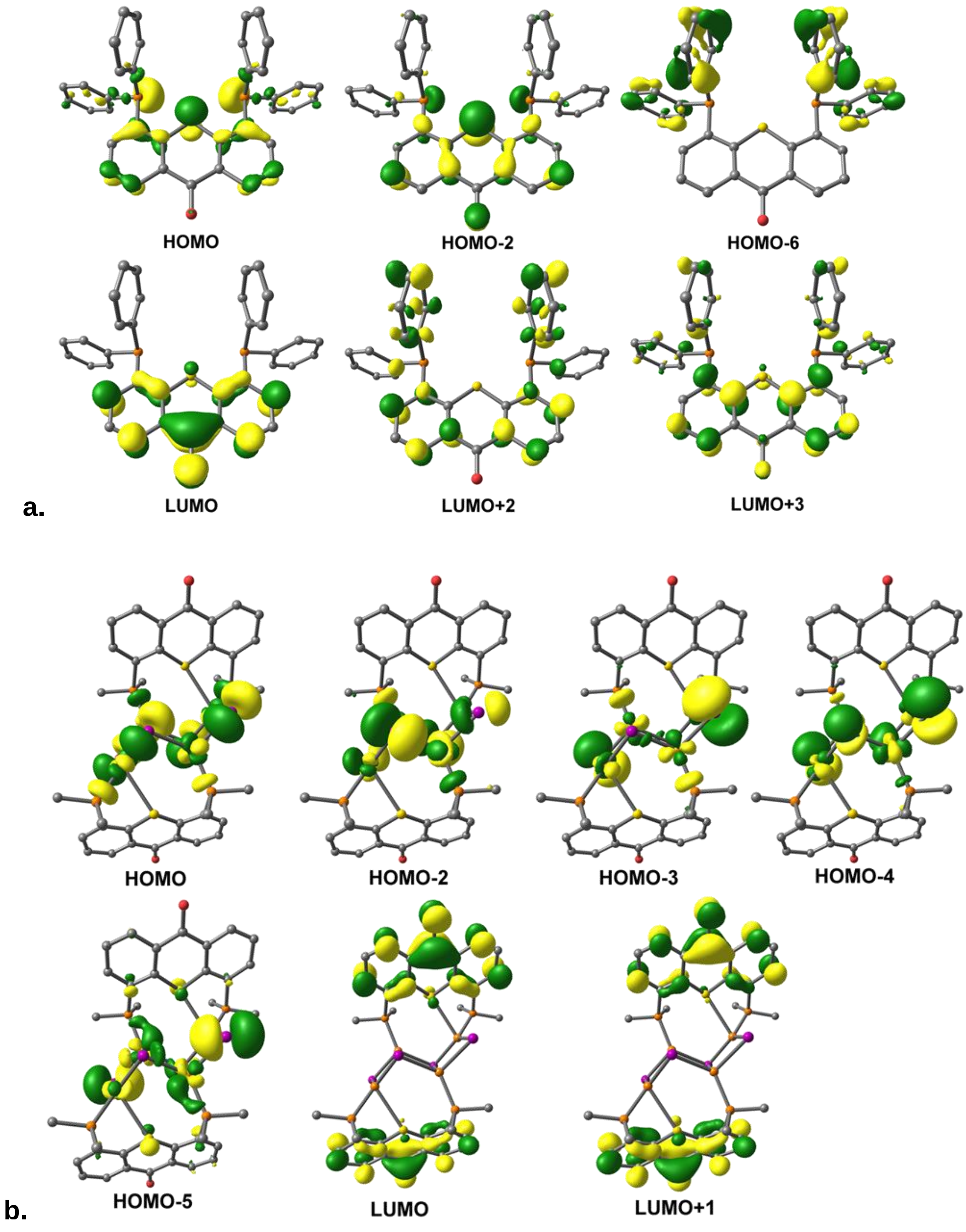


**Figure S38**: Frontier molecular orbitals of **L1*** (a) and **3*** (b) involved in UV-Vis transitions listed in **Tables S8-S9**.
