# Supplementary material for: Assembling Di- and Polynuclear Cu(I) Complexes with Rigid Thioxanthone-Based Ligands: Structures, Reactivity, and Photoluminescence
Source: Inorg Chem. 2024 Dec 16;63(52):24466–81. doi: 10.1021/acs.inorgchem.4c03819 (PMC11688670; doi:10.1021/acs.inorgchem.4c03819)
Supplement: Supplementary file 2 — ic4c03819_si_002.xyz [file ic4c03819_si_002.xyz]

           X              Y              Z       Complex ACu      1.230775    1.644292   -0.604272S      -2.338685    0.361541   -1.135064P       0.518396    0.479889   -2.422618P      -3.403729    2.383875    0.895817O      -4.445419   -3.529700   -1.381661C       0.137923   -2.255070   -2.879315H       1.125244   -2.220800   -3.330116C      -0.561089   -3.465144   -2.834079C      -0.420861   -1.086261   -2.356324C      -3.802325   -0.109687   -0.274436C      -1.698116   -1.152680   -1.756615C      -4.411388   -1.365073   -0.441187C      -2.430165   -2.354827   -1.743014C      -1.833226   -3.506384   -2.286924H      -2.407755   -4.426511   -2.266578C      -5.633228   -1.602457    0.215439H      -6.090470   -2.577783    0.084694C      -4.374387    0.874297    0.562848C      -3.812424   -2.482711   -1.213564C      -5.585811    0.597202    1.199117H      -6.027113    1.340698    1.855969C      -6.221913   -0.634825    1.014247Cu     -1.217965    1.649427    0.650563S       2.336780    0.325910    1.159508P      -0.526822    0.413862    2.429699P       3.412484    2.400066   -0.809591O       4.439246   -3.571836    1.315129C      -0.157084   -2.335329    2.807608H      -1.148201   -2.312470    3.250759C       0.541198   -3.544475    2.734079C       0.407704   -1.152972    2.322898C       3.805683   -0.122944    0.295945C       1.689871   -1.204498    1.732575C       4.413277   -1.382505    0.433059C       2.420981   -2.406514    1.690922C       1.818143   -3.572050    2.197118H       2.391780   -4.492058    2.156143C       5.639552   -1.602190   -0.221454H       6.096196   -2.580645   -0.114113C       4.382645    0.883073   -0.511081C       3.807896   -2.520478    1.170094C       5.598205    0.623100   -1.146640H       6.043577    1.383786   -1.780665C       6.233281   -0.613404   -0.990195H      -7.163449   -0.836530    1.514754H      -0.110446   -4.363187   -3.244051H       7.178247   -0.801660   -1.489472H       0.086137   -4.453174    3.114624H       1.534757    0.088738   -3.312273H      -0.262303    1.248523   -3.311327H      -3.959976    3.346784    0.027256H      -3.976416    2.820250    2.104008H       0.245600    1.147365    3.354725H      -1.555311   -0.003802    3.292941H       3.959004    3.338189    0.091614H       3.995519    2.872118   -1.999260N      -0.013553    3.184393    0.008384C      -0.004101    4.347698   -0.032306C       0.013630    5.790314   -0.084212H      -0.590643    6.134139   -0.928721H      -0.394254    6.192301    0.848523H       1.045357    6.135597   -0.206624Complex CCu      1.994588    1.900536   -0.488172S      -2.046726   -0.004111   -1.281784P       0.726731    1.314868   -2.274687P      -4.166622    1.246923    0.606091O      -2.411747   -4.372215   -2.018640C       1.285767   -1.246694   -3.152304H       2.194219   -0.783928   -3.528201C       1.094208   -2.620935   -3.315282C       0.328166   -0.454808   -2.513914C      -3.270445   -1.097736   -0.642031C      -0.847866   -1.065259   -2.019307C      -3.297720   -2.473733   -0.938078C      -1.056407   -2.449383   -2.191589C      -0.065331   -3.208373   -2.840417H      -0.249077   -4.270925   -2.958607C      -4.349430   -3.253017   -0.420163H      -4.345577   -4.312266   -0.654869C      -4.280877   -0.519737    0.161592C      -2.269519   -3.175242   -1.742939C      -5.305488   -1.328082    0.655998H      -6.075225   -0.887328    1.282564C      -5.343823   -2.695015    0.364851Cu     -1.991326    1.903728    0.488516S       2.046420   -0.007187    1.281981P      -0.724908    1.315781    2.275183P       4.168937    1.241073   -0.604825O       2.403715   -4.376540    2.016109C      -1.288327   -1.245274    3.151588H      -2.196246   -0.781199    3.527211C      -1.098840   -2.619795    3.314320C      -0.329353   -0.454595    2.513683C       3.268844   -1.102526    0.642294C       0.845805   -1.066713    2.019065C       3.293951   -2.478590    0.938151C       1.052122   -2.451236    2.190893C       0.059888   -3.208869    2.839444H       0.241956   -4.271734    2.957358C       4.344989   -3.259240    0.420867H       4.339506   -4.318509    0.655437C       4.280435   -0.525843   -0.160775C       2.264009   -3.178966    1.741767C       5.304254   -1.335532   -0.654642H       6.074863   -0.895797   -1.280828C       5.340653   -2.702501   -0.363420H      -6.144583   -3.311707    0.760020H       1.849799   -3.217279   -3.816443H       6.140940   -3.320188   -0.757996H      -1.855389   -3.215103    3.815280H       1.380504    1.558841   -3.497498H      -0.499298    1.939794   -2.582862H      -5.124577    1.848736   -0.236975H      -4.899327    1.268467    1.806693H       0.502185    1.938340    2.583944H      -1.378667    1.560420    3.497862H       5.126688    1.841064    0.239763H       4.903117    1.262347   -1.804523N       1.717722    3.533342    0.519702C       1.637153    4.547059    1.071065C       1.532559    5.819118    1.758584H       2.038310    6.591892    1.172548H       0.478815    6.085921    1.879495H       2.004707    5.744351    2.742157N      -1.712265    3.535275   -0.520651C      -1.630158    4.548749   -1.072230C      -1.522972    5.820532   -1.759843H      -2.006956    5.750770   -2.738002H      -2.015420    6.596879   -1.167241Complex DCu      1.994588    1.900536   -0.488172S      -2.046726   -0.004111   -1.281784P       0.726731    1.314868   -2.274687P      -4.166622    1.246923    0.606091O      -2.411747   -4.372215   -2.018640C       1.285767   -1.246694   -3.152304H       2.194219   -0.783928   -3.528201C       1.094208   -2.620935   -3.315282C       0.328166   -0.454808   -2.513914C      -3.270445   -1.097736   -0.642031C      -0.847866   -1.065259   -2.019307C      -3.297720   -2.473733   -0.938078C      -1.056407   -2.449383   -2.191589C      -0.065331   -3.208373   -2.840417H      -0.249077   -4.270925   -2.958607C      -4.349430   -3.253017   -0.420163H      -4.345577   -4.312266   -0.654869C      -4.280877   -0.519737    0.161592C      -2.269519   -3.175242   -1.742939C      -5.305488   -1.328082    0.655998H      -6.075225   -0.887328    1.282564C      -5.343823   -2.695015    0.364851Cu     -1.991326    1.903728    0.488516S       2.046420   -0.007187    1.281981P      -0.724908    1.315781    2.275183P       4.168937    1.241073   -0.604825O       2.403715   -4.376540    2.016109C      -1.288327   -1.245274    3.151588H      -2.196246   -0.781199    3.527211C      -1.098840   -2.619795    3.314320C      -0.329353   -0.454595    2.513683C       3.268844   -1.102526    0.642294C       0.845805   -1.066713    2.019065C       3.293951   -2.478590    0.938151C       1.052122   -2.451236    2.190893C       0.059888   -3.208869    2.839444H       0.241956   -4.271734    2.957358C       4.344989   -3.259240    0.420867H       4.339506   -4.318509    0.655437C       4.280435   -0.525843   -0.160775C       2.264009   -3.178966    1.741767C       5.304254   -1.335532   -0.654642H       6.074863   -0.895797   -1.280828C       5.340653   -2.702501   -0.363420H      -6.144583   -3.311707    0.760020H       1.849799   -3.217279   -3.816443H       6.140940   -3.320188   -0.757996H      -1.855389   -3.215103    3.815280H       1.380504    1.558841   -3.497498H      -0.499298    1.939794   -2.582862H      -5.124577    1.848736   -0.236975H      -4.899327    1.268467    1.806693H       0.502185    1.938340    2.583944H      -1.378667    1.560420    3.497862H       5.126688    1.841064    0.239763H       4.903117    1.262347   -1.804523N       1.717722    3.533342    0.519702C       1.637153    4.547059    1.071065C       1.532559    5.819118    1.758584H       2.038310    6.591892    1.172548H       0.478815    6.085921    1.879495H       2.004707    5.744351    2.742157N      -1.712265    3.535275   -0.520651C      -1.630158    4.548749   -1.072230C      -1.522972    5.820532   -1.759843H      -2.006956    5.750770   -2.738002H      -2.015420    6.596879   -1.167241H      -0.468472    6.078704   -1.892361Acetonitrile moleculeN      -0.000510    3.144690   -0.000930C      -0.005460    4.310230   -0.000330C      -0.012270    5.758870    0.001400H      -0.508430    6.130170  -0.901440H      -0.545550    6.125450    0.885690H       1.016460    6.135120    0.029110Ligand L1* S0 geometryP        2.796961000     -0.392263000      0.921719000S        0.000008000      0.837237000      0.265163000P       -2.796954000     -0.392255000      0.921704000C        2.507662000     -4.033345000     -0.948892000H        2.281725000     -5.046413000     -0.626451000C        3.708093000      3.105943000     -1.166136000O        0.000015000      4.678139000     -2.006895000C        2.772505000     -3.767910000     -2.293845000H        2.755015000     -4.573601000     -3.023072000C        4.557598000     -0.364636000      1.496386000C        2.679352000      1.194016000     -0.048940000C        1.398678000      1.744704000     -0.333929000C        2.517770000     -2.995457000     -0.017456000H        2.289635000     -3.205708000      1.024855000C        3.810571000      1.895401000     -0.469637000H        4.793217000      1.490779000     -0.249015000C       -4.557585000     -0.364596000      1.496398000C       -3.810552000      1.895392000     -0.469689000H       -4.793199000      1.490768000     -0.249068000C        1.287549000      2.956094000     -1.042766000C       -3.708066000      3.105923000     -1.166198000C       -2.679338000      1.194010000     -0.048975000C        2.811936000     -1.679021000     -0.409988000C       -1.398660000      1.744699000     -0.333940000C       -1.287520000      2.956085000     -1.042787000C       -2.507812000     -4.033381000     -0.948836000H       -2.281949000     -5.046459000     -0.626376000C        2.458384000      3.623500000     -1.450566000H        2.333328000      4.556071000     -1.989621000C       -2.811972000     -1.679029000     -0.409985000C        4.817334000      0.287714000      2.713965000H        3.999819000      0.757520000      3.256060000C        0.000016000      3.605311000     -1.401597000C       -2.517902000     -2.995482000     -0.017419000H       -2.289822000     -3.205725000      1.024906000C       -2.458351000      3.623479000     -1.450616000H       -2.333292000      4.556042000     -1.989685000C       -4.817290000      0.287711000      2.714003000H       -3.999756000      0.757466000      3.256115000C       -2.772577000     -3.767951000     -2.293810000H       -2.755096000     -4.573659000     -3.023018000C        5.621442000     -0.990958000      0.829377000H        5.441504000     -1.511344000     -0.106389000C       -5.621459000     -0.990852000      0.829368000H       -5.441540000     -1.511211000     -0.106415000C        6.108628000      0.334622000      3.237893000H        6.291698000      0.847903000      4.178280000C        3.050784000     -2.461774000     -2.699397000H        3.251552000     -2.247229000     -3.745988000C       -6.108581000      0.334639000      3.237945000H       -6.291624000      0.847887000      4.178355000C        3.073628000     -1.424602000     -1.764736000H        3.291298000     -0.412912000     -2.093482000C       -7.160265000     -0.289174000      2.562826000H       -8.165522000     -0.262568000      2.974983000C       -3.073574000     -1.424616000     -1.764745000H       -3.291164000     -0.412920000     -2.093526000C        7.160287000     -0.289259000      2.562794000H        8.165547000     -0.262667000      2.974944000C        6.912480000     -0.954624000      1.361366000H        7.725410000     -1.446887000      0.833548000C       -6.912491000     -0.954493000      1.361364000H       -7.725443000     -1.446710000      0.833537000C       -3.050751000     -2.461808000     -2.699394000H       -3.251451000     -2.247257000     -3.745996000H        4.606502000      3.629037000     -1.481304000H       -4.606469000      3.629010000     -1.481394000Ligand L1* T1 geometryP        2.821675000     -0.395974000      0.978372000S       -0.000003000      1.016699000      0.522729000P       -2.821680000     -0.395974000      0.978376000C        2.310309000     -4.014732000     -0.962827000H        2.039850000     -5.020819000     -0.654005000C        3.705228000      3.099210000     -1.185780000O       -0.000005000      4.767280000     -1.933862000C        2.566121000     -3.737571000     -2.308001000H        2.496162000     -4.527831000     -3.050479000C        4.643086000     -0.408642000      1.469110000C        2.700871000      1.223819000     -0.007983000C        1.440205000      1.808051000     -0.243531000C        2.391199000     -2.995597000     -0.011939000H        2.175483000     -3.214147000      1.030963000C        3.834958000      1.884278000     -0.483657000H        4.818448000      1.462567000     -0.312045000C       -4.643095000     -0.408651000      1.469102000C       -3.834965000      1.884278000     -0.483650000H       -4.818456000      1.462568000     -0.312037000C        1.276720000      3.023532000     -0.983444000C       -3.705237000      3.099211000     -1.185773000C       -2.700878000      1.223819000     -0.007978000C        2.741437000     -1.691504000     -0.392371000C       -1.440212000      1.808051000     -0.243528000C       -1.276728000      3.023532000     -0.983442000C       -2.310282000     -4.014731000     -0.962817000H       -2.039821000     -5.020816000     -0.653992000C        2.463685000      3.653141000     -1.434195000H        2.359970000      4.581480000     -1.982032000C       -2.741426000     -1.691505000     -0.392365000C        4.989060000      0.250247000      2.659277000H        4.221259000      0.754629000      3.240967000C       -0.000004000      3.631940000     -1.291365000C       -2.391184000     -2.995595000     -0.011930000H       -2.175476000     -3.214144000      1.030973000C       -2.463695000      3.653141000     -1.434190000H       -2.359980000      4.581481000     -1.982027000C       -4.989080000      0.250241000      2.659264000H       -4.221286000      0.754631000      3.240957000C       -2.566085000     -3.737572000     -2.307993000H       -2.496117000     -4.527833000     -3.050470000C        5.640807000     -1.073914000      0.744481000H        5.389795000     -1.594678000     -0.174298000C       -5.640807000     -1.073933000      0.744470000H       -5.389787000     -1.594700000     -0.174305000C        6.310992000      0.259623000      3.106690000H        6.565346000      0.777036000      4.027761000C        2.906001000     -2.439610000     -2.696195000H        3.103110000     -2.217176000     -3.741558000C       -6.311015000      0.259611000      3.106669000H       -6.565378000      0.777027000      4.027736000C        2.996334000     -1.421140000     -1.743552000H        3.264025000     -0.416659000     -2.056760000C       -7.301321000     -0.404310000      2.377239000H       -8.329466000     -0.404788000      2.728708000C       -2.996313000     -1.421143000     -1.743549000H       -3.264006000     -0.416663000     -2.056760000C        7.301307000     -0.404289000      2.377263000H        8.329449000     -0.404762000      2.728738000C        6.962908000     -1.072718000      1.198895000H        7.727617000     -1.593432000      0.628607000C       -6.962911000     -1.072742000      1.198876000H       -7.727613000     -1.593464000      0.628585000C       -2.905968000     -2.439613000     -2.696190000H       -3.103069000     -2.217181000     -3.741556000H        4.598408000      3.602098000     -1.547111000H       -4.598417000      3.602098000     -1.547103000Complex 3* S0 geometryI        0.937889000     -0.611747000      1.904156000I        2.045228000     -2.138817000     -2.120078000Cu       0.028701000      1.485930000      0.517495000Cu       2.681560000     -1.110036000      0.123764000P        1.245911000      3.355465000      0.149820000S        3.638145000      1.514962000     -0.891958000P        4.881385000     -0.943168000      0.457372000C        3.165911000      3.748331000      3.697851000H        2.972468000      3.423457000      4.723436000C        2.208191000      4.951371000     -3.543666000O        5.327620000      2.634170000     -4.834091000C        4.270673000      4.525314000      3.408769000H        4.954695000      4.822047000      4.208856000C        0.129388000      4.799468000      0.227158000C        2.088006000      3.645653000     -1.469612000C        3.122428000      2.808388000     -1.947774000C        2.323743000      3.357082000      2.695784000H        1.464443000      2.723079000      2.932679000C        1.674288000      4.689080000     -2.288782000H        0.882465000      5.345663000     -1.930155000C        5.771188000     -2.482490000      0.798287000C        6.647193000     -0.971882000     -1.740695000H        7.118283000     -1.816854000     -1.234051000C        3.705498000      3.072326000     -3.187987000C        7.125899000     -0.571729000     -2.987797000C        5.582672000     -0.326516000     -1.112734000C        2.514659000      3.772290000      1.390198000C        4.948220000      0.760542000     -1.759251000C        5.416017000      1.171390000     -3.006676000C        5.540841000      0.939019000      3.990605000H        5.114944000      0.869680000      4.995082000C        3.220136000      4.140431000     -3.967500000H        3.713112000      4.286092000     -4.930950000C        5.519278000      0.197955000      1.709112000C       -1.091789000      4.727237000     -0.423244000H       -1.391386000      3.799888000     -0.915436000C        4.850129000      2.308028000     -3.744165000C        5.001585000      0.159797000      2.975561000H        4.158166000     -0.498595000      3.203634000C        6.485732000      0.486614000     -3.589025000H        6.801492000      0.852067000     -4.568942000C        5.265279000     -3.664387000      0.295457000H        4.358185000     -3.654414000     -0.316054000C        6.564311000      1.786677000      3.731581000H        6.979629000      2.404910000      4.533224000C        0.473386000      5.984660000      0.861529000H        1.424812000      6.080598000      1.386003000C        6.924217000     -2.529862000      1.577470000H        7.342170000     -1.611470000      1.994188000C       -1.944485000      5.827012000     -0.452021000H       -2.895931000      5.748311000     -0.984090000C        4.496712000      4.908262000      2.136563000H        5.367431000      5.519624000      1.882959000C        5.903606000     -4.869366000      0.556978000H        5.487834000     -5.791583000      0.141682000C        3.604534000      4.549146000      1.118747000H        3.798603000      4.903006000      0.103075000C        7.029891000     -4.903645000      1.323971000H        7.527408000     -5.853944000      1.536347000C        6.555006000      1.074226000      1.469642000H        6.992802000      1.141504000      0.470533000C       -1.592334000      6.972880000      0.178873000H       -2.265077000      7.835880000      0.165309000C       -0.405155000      7.053380000      0.846539000H       -0.128884000      7.976598000      1.363969000C        7.541267000     -3.727767000      1.832036000H        8.444718000     -3.747275000      2.448183000C        7.071209000      1.872474000      2.474703000H        7.891548000      2.558286000      2.246907000I       -0.937958000      0.611696000     -1.904169000I       -2.045228000      2.138809000      2.120006000Cu      -0.028657000     -1.485900000     -0.517485000Cu      -2.681561000      1.110028000     -0.123835000P       -1.245866000     -3.355435000     -0.149810000S       -3.638169000     -1.514975000      0.892026000P       -4.881409000      0.943155000     -0.457304000C       -3.165935000     -3.748344000     -3.697783000H       -2.972513000     -3.423477000     -4.723379000C       -2.208147000     -4.951341000      3.543676000O       -5.327644000     -2.634184000      4.834159000C       -4.270628000     -4.525284000     -3.408760000H       -4.954653000     -4.822014000     -4.208845000C       -0.129412000     -4.799481000     -0.227090000C       -2.087961000     -3.645623000      1.469622000C       -3.122429000     -2.808396000      1.947703000C       -2.323743000     -3.357090000     -2.695855000H       -1.464448000     -2.723099000     -2.932795000C       -1.674243000     -4.689050000      2.288791000H       -0.882397000     -5.345614000      1.930187000C       -5.771143000      2.482520000     -0.798278000C       -6.647217000      0.971869000      1.740763000H       -7.118317000      1.816849000      1.234135000C       -3.705522000     -3.072339000      3.188055000C       -7.125854000      0.571759000      2.987807000C       -5.582672000      0.326508000      1.112662000C       -2.514614000     -3.772260000     -1.390189000C       -4.948244000     -0.760555000      1.759319000C       -5.416041000     -1.171403000      3.006744000C       -5.540922000     -0.938950000     -3.990544000H       -5.115087000     -0.869605000     -4.995050000C       -3.220160000     -4.140444000      3.967568000H       -3.713115000     -4.286131000      4.931023000C       -5.519233000     -0.197925000     -1.709102000C        1.091834000     -4.727207000      0.423254000H        1.391437000     -3.799859000      0.915440000C       -4.850084000     -2.307998000      3.744174000C       -5.001541000     -0.159767000     -2.975552000H       -4.158128000      0.498617000     -3.203659000C       -6.485756000     -0.486627000      3.589093000H       -6.801535000     -0.852053000      4.569012000C       -5.265280000      3.664379000     -0.295529000H       -4.358191000      3.654440000      0.315994000C       -6.564335000     -1.786690000     -3.731513000H       -6.979641000     -2.404914000     -4.533167000C       -0.473341000     -5.984630000     -0.861519000H       -1.424760000     -6.080578000     -1.386003000C       -6.924217000      2.529854000     -1.577541000H       -7.342178000      1.611468000     -1.994254000C        1.944461000     -5.827025000      0.452089000H        2.895901000     -5.748329000      0.984175000C       -4.496667000     -4.908232000     -2.136554000H       -5.367382000     -5.519597000     -1.882944000C       -5.903606000      4.869358000     -0.557050000H       -5.487819000      5.791572000     -0.141765000C       -3.604489000     -4.549116000     -1.118738000H       -3.798566000     -4.902973000     -0.103066000C       -7.029891000      4.903637000     -1.324043000H       -7.527417000      5.853930000     -1.536420000C       -6.555030000     -1.074239000     -1.469574000H       -6.992801000     -1.141483000     -0.470455000C        1.592379000     -6.972850000     -0.178863000H        2.265128000     -7.835844000     -0.165300000C        0.405200000     -7.053350000     -0.846530000H        0.128949000     -7.976549000     -1.364005000C       -7.541223000      3.727797000     -1.832027000H       -8.444682000      3.747304000     -2.448169000C       -7.071233000     -1.872487000     -2.474635000H       -7.891555000     -2.558319000     -2.246831000H        1.837974000      5.779956000     -4.149557000H        7.960680000     -1.085018000     -3.467430000H       -7.960617000      1.085076000      3.467445000H       -1.837903000     -5.779924000      4.149556000Complex 3* T1 geometryI       -1.133551000     -0.747292000     -1.865137000I       -1.684925000     -2.464489000      2.238969000Cu      -0.277408000      1.570708000     -0.653535000Cu      -2.859370000     -1.412511000      0.092493000P       -1.648711000      3.532938000     -0.404083000S       -3.975635000      1.545189000      0.855571000P       -5.195702000     -1.266057000     -0.192927000C       -3.647603000      3.354484000     -4.017579000H       -3.482775000      2.865378000     -4.973263000C       -2.864807000      5.424245000      3.112018000O       -5.843262000      3.066396000      4.628564000C       -4.761120000      4.176470000     -3.830366000H       -5.465305000      4.331965000     -4.642795000C       -0.537060000      5.040096000     -0.593677000C       -2.573889000      3.914081000      1.215161000C       -3.579637000      3.076313000      1.755025000C       -2.744898000      3.149307000     -2.972097000H       -1.886861000      2.499053000     -3.117399000C       -2.236976000      5.076721000      1.912308000H       -1.473471000      5.732478000      1.512439000C       -6.026354000     -2.949266000     -0.163356000C       -7.069843000     -0.886741000      1.918509000H       -7.517708000     -1.797470000      1.535826000C       -4.240228000      3.431205000      2.945248000C       -7.608907000     -0.283468000      3.059503000C       -5.961818000     -0.345675000      1.265107000C       -2.947434000      3.776688000     -1.736156000C       -5.388431000      0.845963000      1.767540000C       -5.907228000      1.447925000      2.925158000C       -5.727951000     -0.059680000     -4.107134000H       -5.198634000     -0.205534000     -5.044407000C       -3.861030000      4.610614000      3.614527000H       -4.384489000      4.853043000      4.531761000C       -5.893266000     -0.431750000     -1.717857000C        0.718582000      5.015646000      0.029638000H        1.031963000      4.137521000      0.587234000C       -5.355171000      2.668182000      3.568968000C       -5.216463000     -0.612204000     -2.931673000H       -4.288657000     -1.176236000     -2.958022000C       -7.022711000      0.864598000      3.558150000H       -7.397827000      1.350372000      4.451268000C       -5.436810000     -3.960933000      0.608561000H       -4.527873000     -3.757824000      1.168213000C       -6.909820000      0.684838000     -4.074651000H       -7.305259000      1.117661000     -4.989267000C       -0.922433000      6.170484000     -1.324961000H       -1.890231000      6.203537000     -1.813951000C       -7.189114000     -3.223781000     -0.895015000H       -7.651280000     -2.449955000     -1.499724000C        1.569427000      6.118337000     -0.059827000H        2.537719000      6.090118000      0.430626000C       -4.971156000      4.794866000     -2.595424000H       -5.837497000      5.432707000     -2.444087000C       -6.013946000     -5.231068000      0.657039000H       -5.551634000     -6.008457000      1.258711000C       -4.066332000      4.599114000     -1.549931000H       -4.233901000      5.088527000     -0.595134000C       -7.174119000     -5.502162000     -0.072885000H       -7.618850000     -6.492753000     -0.039837000C       -7.076489000      0.317218000     -1.687064000H       -7.607855000      0.464434000     -0.751549000C        1.182445000      7.243780000     -0.791768000H        1.848535000      8.098579000     -0.869047000C       -0.060541000      7.265618000     -1.427293000H       -0.365892000      8.136466000     -2.000580000C       -7.758469000     -4.498935000     -0.849186000H       -8.658913000     -4.705358000     -1.420849000C       -7.579847000      0.876148000     -2.864326000H       -8.497389000      1.457032000     -2.833664000I        0.984962000      0.715812000      1.765617000I        2.012008000      1.914669000     -2.307286000Cu       0.320660000     -1.657165000      0.459333000Cu       2.891376000      0.707033000     -0.115431000P        1.747300000     -3.494534000     -0.129775000S        4.346304000     -1.473427000     -0.566073000P        5.032879000      1.313044000      0.784122000C        3.501960000     -3.978638000      3.588755000H        3.285842000     -3.647764000      4.600668000C        3.261207000     -4.535280000     -3.864768000O        6.672541000     -2.302882000     -4.325214000C        4.617488000     -4.779683000      3.333611000H        5.272376000     -5.076156000      4.148226000C        0.633680000     -5.002339000     -0.335440000C        2.805029000     -3.518480000     -1.704243000C        3.899768000     -2.663776000     -1.907473000C        2.663206000     -3.592327000      2.540852000H        1.799470000     -2.964247000      2.741390000C        2.490115000     -4.458018000     -2.701117000H        1.655126000     -5.132601000     -2.564666000C        5.432858000      3.122375000      0.977423000C        7.241823000      1.404605000     -1.013040000H        7.545502000      2.296832000     -0.478544000C        4.736852000     -2.767225000     -3.043215000C        7.964302000      0.950097000     -2.123978000C        6.098694000      0.683706000     -0.601792000C        2.934295000     -4.010636000      1.231672000C        5.711446000     -0.467675000     -1.292353000C        6.385989000     -0.908391000     -2.449539000C        5.144497000      0.001205000      4.701792000H        4.524873000      0.112940000      5.586762000C        4.363627000     -3.717407000     -4.026517000H        4.994877000     -3.777591000     -4.904905000C        5.555247000      0.470399000      2.361973000C       -0.563944000     -4.852990000     -1.049732000H       -0.830954000     -3.886777000     -1.466581000C        5.974895000     -2.013537000     -3.297871000C        4.757435000      0.608741000      3.507011000H        3.836650000      1.183347000      3.468261000C        7.542986000     -0.160635000     -2.832552000H        8.070535000     -0.505366000     -3.713871000C        5.030093000      4.008724000     -0.032735000H        4.469128000      3.644804000     -0.888916000C        6.319510000     -0.754215000      4.755344000H        6.615949000     -1.231352000      5.685088000C        0.957228000     -6.251739000      0.207040000H        1.879184000     -6.386133000      0.762536000C        6.136613000      3.601776000      2.090171000H        6.452549000      2.921878000      2.874240000C       -1.417494000     -5.942466000     -1.232002000H       -2.338080000     -5.815807000     -1.795230000C        4.892994000     -5.195168000      2.028285000H        5.761486000     -5.815233000      1.824275000C        5.352579000      5.362785000      0.062542000H        5.049278000      6.042784000     -0.728092000C        4.054730000     -4.813305000      0.978724000H        4.278831000     -5.133530000     -0.034506000C        6.056927000      5.841010000      1.171829000H        6.302099000      6.896575000      1.245955000C        6.732945000     -0.286612000      2.416012000H        7.353839000     -0.394161000      1.532221000C       -1.091990000     -7.186798000     -0.686054000H       -1.759308000     -8.033352000     -0.821730000C        0.093641000     -7.337255000      0.035456000H        0.353826000     -8.301342000      0.463708000C        6.444268000      4.960866000      2.184425000H        6.993463000      5.327286000      3.046860000C        7.110413000     -0.897895000      3.613418000H        8.024244000     -1.483648000      3.651328000H       -2.576820000      6.333133000      3.630754000H       -8.472931000     -0.721249000      3.549211000H        8.852357000      1.490049000     -2.442096000H        2.999753000     -5.257164000     -4.634004000 
